# iPSC-Derived Regulatory Dendritic Cells Inhibit Allograft Rejection by Generating Alloantigen-Specific Regulatory T Cells

**DOI:** 10.1016/j.stemcr.2017.03.020

**Published:** 2017-04-20

**Authors:** Songjie Cai, Jiangang Hou, Masayuki Fujino, Qi Zhang, Naotsugu Ichimaru, Shiro Takahara, Ryoko Araki, Lina Lu, Ji-Mei Chen, Jian Zhuang, Ping Zhu, Xiao-Kang Li

**Affiliations:** 1 Division of Transplantation Immunology, National Research Institute for Child Health and Development, 2-10-1 Okura, Setagaya-ku, Tokyo 157-8535, Japan; 2 Department of Advanced Technology for Transplantation, Osaka University Graduate School of Medicine, Osaka 565-0871, Japan; 3 Huashan Hospital, Fudan University, Shanghai 200032, China; 4 AIDS Research Center, National Institute of Infectious Diseases, Tokyo 162-8640, Japan; 5 Department of Basic Medical Sciences for Radiation Damages, National Institute of Radiological Sciences, Chiba 263-8555, Japan; 6 Department of Immunology, Lerner Research Institute, Cleveland Clinic, Cleveland, OH 44195, USA; 7 Department of Cardiac Surgery, Guangdong Cardiovascular Institute, Guangdong General Hospital, Guangdong Academy of Medical Sciences, Guangzhou 510100, China

**Keywords:** iPSC, regulatory dendritic cells, regulatory T cells, antigen-specific tolerance, murine cardiac allotransplant, TGF-β1

## Abstract

Regulatory dendritic cell (DCregs)-based immunotherapy is a potential therapeutic tool for transplant rejection. We generated DCregs from murine induced pluripotent stem cells (iPSCs), which could remain in a “stable immature stage” even under strong stimulation. Harnessing this characteristic, we hypothesized that iPS-DCregs worked as a negative vaccine to generate regulatory T cells (Tregs), and induced donor-specific allograft acceptance. We immunized naive CBA (H-2K^k^) mice with B6 (H-2K^b^) iPS-DCregs and found that Tregs (CD4^+^CD25^+^FOXP3^+^) significantly increased in CBA splenocytes. Moreover, immunized CBA recipients permanently accepted B6 cardiac grafts in a donor-specific pattern. We demonstrated mechanistically that donor-type iPS-DCregs triggered transforming growth factor β1 secretion, under which the donor-antigen peptides directed naive CD4^+^ T cells to differentiate into donor-specific FOXP3^+^ Tregs instead of into effector T cells in vivo. These findings highlight the potential of iPS-DCregs as a key cell therapy resource in clinical transplantation.

## Highlights

• iPS-DCregs keep in stable immature stage that makes them a powerful cellular vaccine
• Donor-type iPS-DCregs lead to permanent acceptance of allogeneic cardiac grafts
• iPS-DCregs reduce CTL and downregulate proinflammatory cytokine
• iPS-DCregs enhance Tregs transmigration capability in a TGF-β1-dependent manner

## Graphical Abstract

**Figure:**
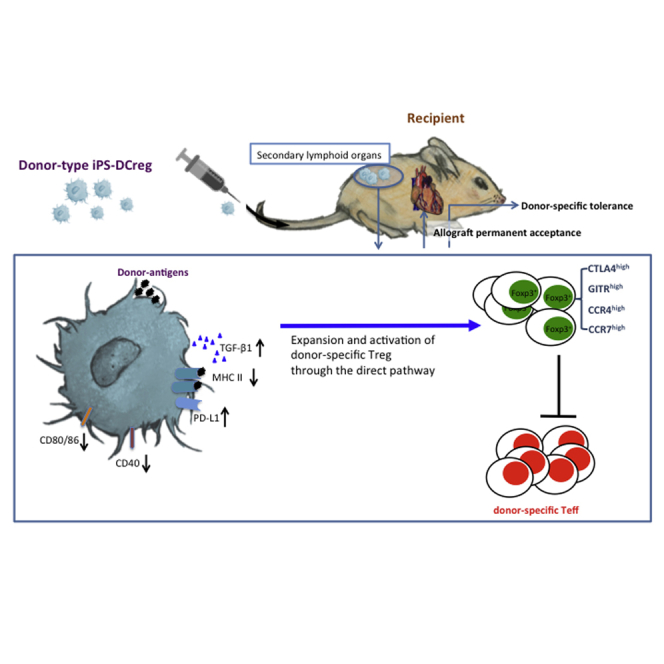

## Teaser

In this article, Li and colleagues generated DCregs from murine iPSCs and demonstrated mechanistically that donor-type iPS-DCregs triggered TGF-β1 secretion, under which the donor-antigen peptides directed naive CD4^+^ T cells to differentiate into donor-specific Tregs instead of into Teffs in vivo. These findings highlight the potential of iPS-DCregs as a key cell therapy resource in clinical transplantation.

## Introduction

The main form of therapy for allograft rejection is immunosuppressive (IS) drugs. Unfortunately, non-specific immunosuppression often causes numerous adverse side effects, such as opportunistic infection and cancer (Dantal et al., 1998), and also fails to induce antigen-specific tolerance. Thus, reducing the use of IS drugs and inducing donor-specific tolerance are the main objectives in transplantation.

Regulatory immune cell therapy, including regulatory T cells (Tregs) (Bradley, 2014, McMurchy et al., 2011), regulatory dendritic cells (DCregs) (Ezzelarab and Thomson, 2011, Moreau et al., 2012), and immature DCs (iDCs) (Roncarolo et al., 2001), is an emerging strategy for the prevention of allograft rejection by promoting antigen-specific tolerance and the elimination of IS drug use (Raich-Regue et al., 2014, Wood et al., 2012). Because DCregs play essential roles in maintaining immune homeostasis (Morelli and Thomson, 2007), they are usually the target of rejection treatment. However, the lack of stable therapeutic DCregs has been the biggest problem in clinical application. Induced pluripotent stem cells (iPSCs), created by Yamanaka and colleagues in 2006, can propagate indefinitely and differentiate into various cells just like embryonic stem cells (ESCs) (Takahashi et al., 2007, Takahashi and Yamanaka, 2006). Notably, unlike ESCs, iPSCs can be generated from adult cells, which overcomes ethical issues and patient-matching limitations. In our previous study (Zhang et al., 2014), we established a novel approach for generating a sufficient quantity of high-quality functional DCregs from iPSCs (iPS-DCregs), which could be kept in a “stable immature stage” even under strong stimulation. Harnessing this characteristic, we hypothesized that donor-type iPS-DCregs expressing donor antigen worked as an immune suppressive vaccine to generate alloantigen-specific Tregs, and induced permanent acceptance of mouse cardiac allografts.

## Results

### iPS-DCregs Are Maintained in a “Stable Immature Stage” Even under IFN-γ Stimulation

The morphology of iPS-DCregs is similar to that of bone marrow DCregs (BM-DCregs), which are smaller and have shorter dendrites. They express low levels of co-stimulatory molecules (CD40, CD80, and CD86) and major histocompatibility complex (MHC) class II antigens, and a high percentage of CD11b^+^CD11c^+^ compared with conventional DCs (DCcons) (Hackstein and Thomson, 2004, Morelli and Thomson, 2003, Morelli and Thomson, 2007) (Figure S1B). In contrast to other DC types (BM-DCcons, iPS-DCcons, and BM-DCregs), even under interferon-γ (IFN-γ) stimulation, iPS-DCregs always maintain a high antigen uptake ability, in fluorescein isothiocyanate uptake tests with both ovalbumin (OVA) and dextran, which indicates that iPS-DCregs can be kept in a “stable immature stage” (Figure S2).

### iPS-DCregs Modulate T Cell Proliferation in Direct and Indirect Pathways

We detected that mRNA expression of suppressive cytokines transforming growth factor β1 (TGF-β1), Arg-1, PD-L1, and HO-1 in iPS-DCregs was significantly higher than in BM-DCcons (Figures 6A and S1C). According to the stable immature and suppressive characteristics of the iPS-DCregs, we hypothesized that they could play a role as an immune-suppressive vaccine in allo-rejection.

**Figure 1:**
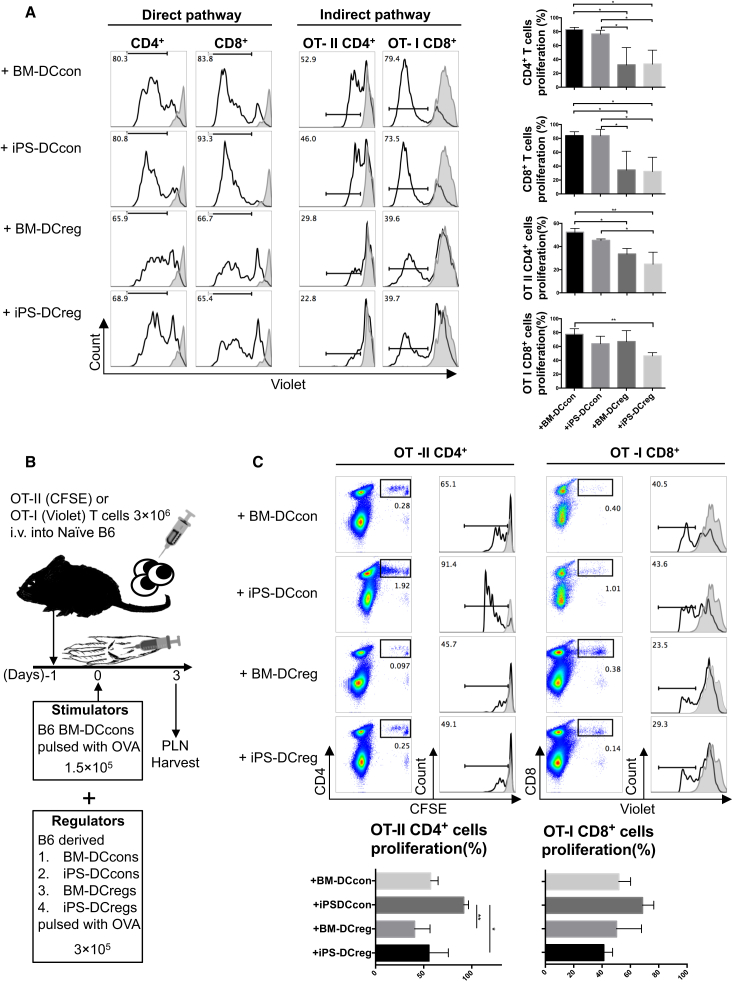
iPS-DCregs Suppress T Cell Proliferative Responses In Vitro and In Vivo (A) Addition of iPS-DCregs suppresses T cell proliferation in allo-MLR (left panel) and OVA-specific MLR (right panel) (n = 3–5 in each group, mean ± SD, pooled from three independent experiments). The details of these two reaction systems are shown in Figure S5. Proliferation of T cells was determined by Violet dilution gated on the CD4^+^ and/or CD8^+^ population. Gray lines are T cells without BM-DCcons stimulation. Statistical analysis was determined by one-way ANOVA and Tukey's test. ^∗^p < 0.05, ^∗∗^p < 0.01. (B) Schematic of PLNA protocol. (C) Addition of iPS-DCregs suppresses T cell proliferative responses in PLNA (n = 3 in each group, mean ± SD, pooled from three independent experiments). Proliferation of T cells was determined by carboxyfluorescein diacetate succinimidyl ester (CFSE) or Violet dilution gated on CD4^+^ (OT-II) or CD8^+^ (OT-I) population. Gray lines indicate PBS injection into the footpad, used as a negative control. Statistical analysis was determined by one-way ANOVA and Tukey's test. ^∗^p < 0.05, ^∗∗^p < 0.01.

To identify this role, we first set up an allogeneic mixed lymphocyte reaction (MLR) to examine the direct antigen-presenting regulatory function of iPS-DCregs (Figure S5A). naive T cells isolated from CBA mice were stimulated with B6 BM-DCcons resulting in an alloreactive proliferation. In this allogeneic MLR system, the addition of B6 BM-DCregs and iPS-DCregs significantly inhibited the proliferated response in the population of CD4^+^ and CD8^+^ T cells (Figure 1A, left).

We next established OVA-specific MLR to investigate the indirect antigen-presenting regulatory ability of iPS-DCregs (Figure S5B). T cells isolated from B6-background T cell receptor (TCR) transgenic mice OT-II (CD4^+^) and OT-I (CD8^+^) were co-cultured with OVA-pulsed B6 BM-DCcons at a 20:1 ratio for 3 days, which led to fierce OVA-reaction T cell proliferation. The addition of B6 BM-DCregs and iPS-DCregs markedly inhibited the proliferated response (Figure 1A, right).

In addition, we tested in vivo regulatory function using a proliferation lymph node assay (PLNA). Wild-type B6 mice were adoptively transferred with 3 × 10^6^ OT-II or OT-I T cells the day before. OVA-pulsed B6 BM-DCcons (1.5 × 10^5^) combined with iPS-DCregs or other controls (3 × 10^5^) were injected subcutaneously into the recipients' footpads (Figure 1B). The OVA-specific reactive T cells isolated from the PLNs were intensely proliferated in the BM-DCcons and iPS-DCcons groups, but were significantly inhibited in the BM-DCregs and iPS-DCregs groups (Figure 1C).

### Immunization with iPS-DCregs Generates Tregs In Vivo, Leading to Teff Suppression

**Figure 2:**
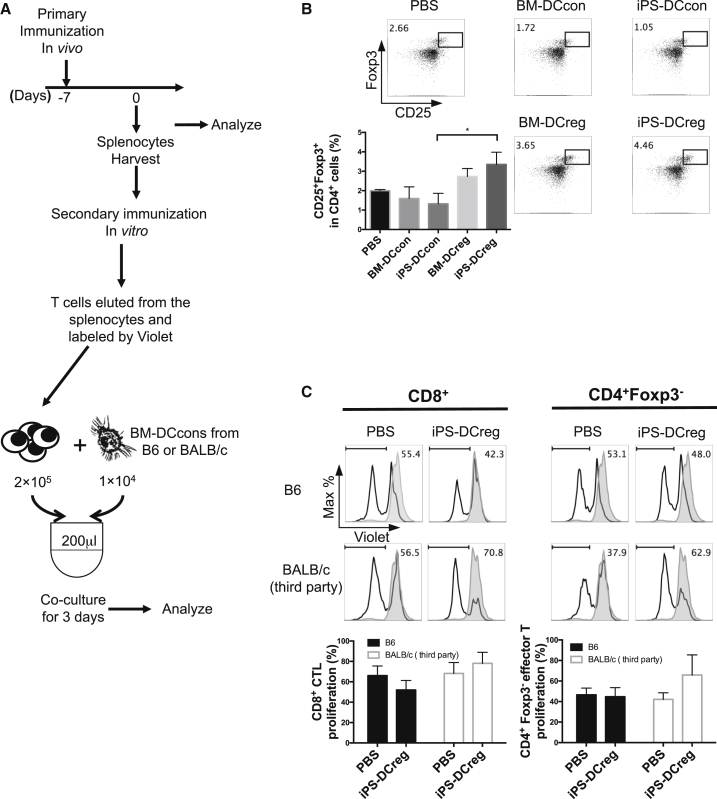
Administration of iPS-DCregs Generates Tregs In Vivo (A) CBA mice were immunized with four types of B6 DCs (BM-DCcons, iPS-DCcons, BM-DCregs, iPS-DCregs) on day −7 and were euthanized on day 0. SPCs were harvested for FCM and one-way MLR (secondary immunization). (B) The percentage of CD25^+^ FOXP3^+^ cells in the spleen from iPS-DCregs-immunized CBA was significantly increased compared with other groups (n = 4 in the PBS control group, n = 3 each in other groups, mean ± SD, pooled from three independent experiments). Statistical analysis was determined by one-way ANOVA and Tukey's test. ^∗^p < 0.05. (C) T cells from B6 iPS-DCregs immunized with CBA were co-cultured with B6 BM-DCcons for 3 days. Proliferation of Teffs was determined by Violet dilution gated on CD8^+^ or CD4^+^ FOXP3^−^ population (n = 3 in each group, mean ± SD, pooled from three independent experiments). Statistical analysis was determined by one-way ANOVA and Tukey's test. No statistically significant differences were observed between these groups.

Next, we immunized CBA mice with B6-derived DCs and isolated their spleens 7 days after immunization. The spleen cells (SPCs) were assessed by flow cytometry (FCM) and set up for secondary immunization in vitro (Tiao et al., 2005) (Figure 2A).

We identified that the CD25^+^FOXP3^+^ population was significantly increased in the iPS-DCregs-immunized group (Figure 2B). We then eluted T cells from these SPCs by nylon column and co-cultured them with B6 BM-DCcons or BALB/c (H-2K^d^) BM-DCcons (third party) for MLR, which served as the secondary immunization. After 3 days of co-culture, stimulation of the BALB/c BM-DCcons aggravated the proliferation of CD8^+^ and CD4^+^FOXP3^−^ effector T cells (Teffs) in the immunized group of T B6 iPS-DCregs compared with the negative control (CBA treated with PBS). However, the results of co-culturing with B6 BM-DCcons showed the suppression of Teff proliferation, especially in CD8^+^ T cells (Figure 2C). This test showed that donor-type iPS-DCregs immunization directly generated Tregs in vivo, and led to donor-specific Teff suppression.

### Donor-type iPS-DCregs Immunization Leads to Permanent Acceptance of Allogeneic Cardiac Grafts

Some studies have shown that recipient-type DCregs loaded with donor-antigen peptide work more efficiently than donor-type DCregs (Garrovillo et al., 1999, Ali et al., 2000). In the preliminary experiment, we tested donor-type BM-DCregs and recipient-type BM-DCregs (with or without donor-antigen pulsing). Three types of DCregs (B6 BM-DCregs, CBA BM-DCregs, and CBA BM-DCregs pulsed with H-2K^b^ antigen peptide for 48 hr) were injected intravenously (1 × 10^6^) into CBA recipients at 7 days prior to receiving a B6 heart allograft. We found that donor-type BM-DCregs prolonged allograft survival (PBS control n = 13, median survival time (MST) 8 days; B6 BM-DCregs n = 6, MST 25.5 days). However, neither recipient-type BM-DCregs-loaded nor recipient-type BM-DCregs-loaded donor antigen could protect allografts from acute rejection (CBA BM-DCregs n = 3, MST 7 days; CBA BM-DCregs pulsed with H-2K^b^ antigen peptide n = 3, MST 7 days) (Figure S3A).

**Figure 3:**
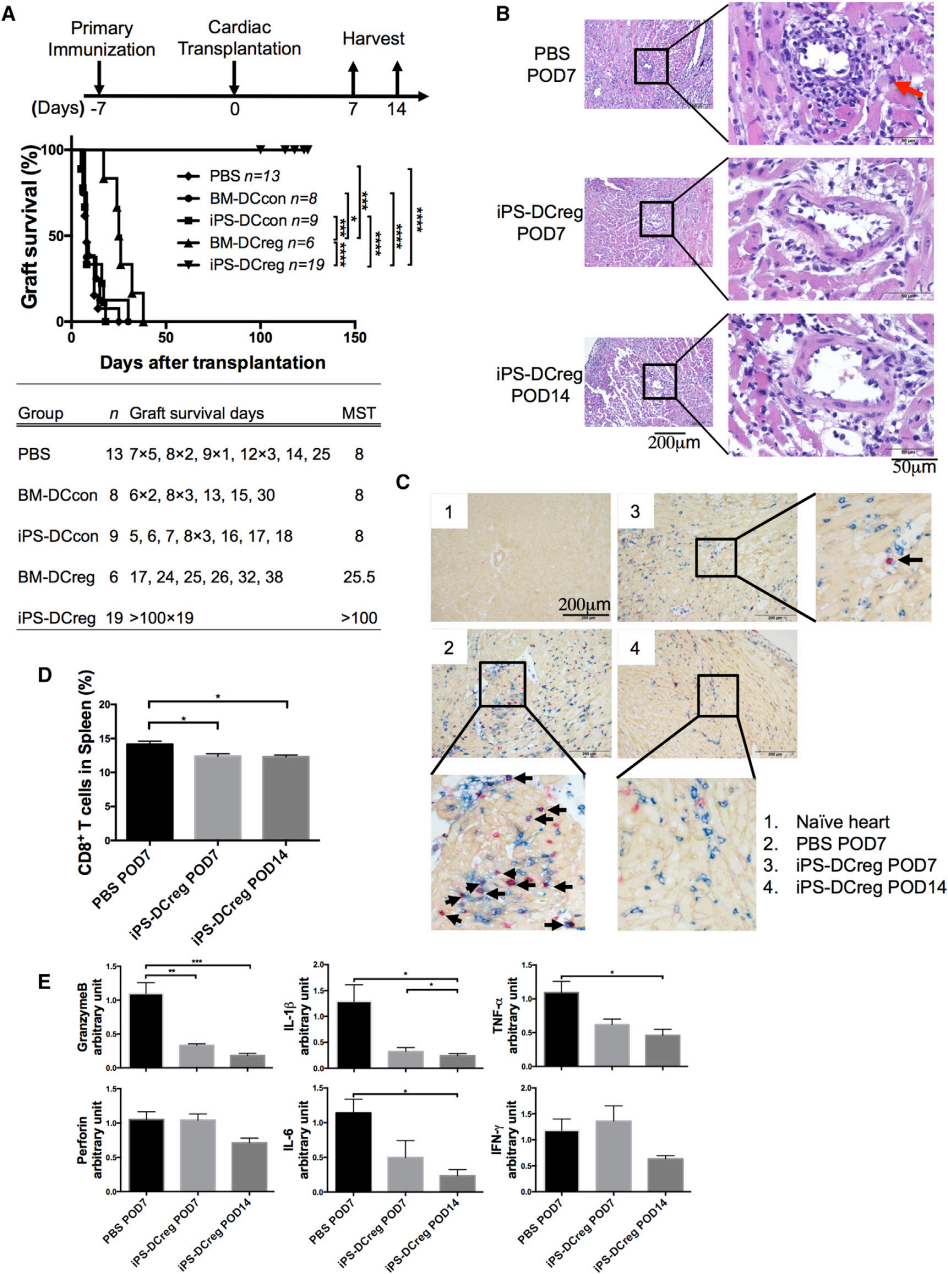
iPS-DCreg Immunization Induces Permanent Acceptance of Allogeneic Cardiac Grafts and Decreases CD8^+^ T Cells in Grafts and Spleen (A) 1 × 10^6^ B6 DCs were injected intravenously into CBA mice 7 days before heterotopic cardiac transplantation. A statistical evaluation of graft survival was performed using Kaplan-Meier curves and compared using log-rank tests. ^∗^p < 0.05, ^∗∗∗^p < 0.001, ^∗∗∗∗^p < 0.0001. (B) Cardiac allografts of POD7 and POD14 were stained with H&E. The red arrow indicates the increased lymphocytic infiltration around the coronary artery in PBS treated allograft. (C) Allografts were stained with anti-CD8 (blue), collagen IV (yellowish-brown), and BrdU (red) by triple immunostaining. The black arrows indicate the CD8 and BrdU double positive lymphocytes in the grafts. (D) Spleen cells (SPCs) were harvested on POD7 and POD14 for CD8^+^ T cell assessment by FCM analysis (n = 4 in each group, mean ± SD, pooled from four independent experiments). ^∗^p < 0.05. (E) The mRNA expression of GranzymeB and Perforin, IFN-γ, TNF-α, IL-1β, and IL-6 in allografts was measured by qRT-PCR (n = 9 in the PBS-POD7 group, n = 6 in the iPS-DCregs POD7 group, n = 5 in the iPS-DCregs POD14 group; mean ± SD, pooled from five independent experiments). Statistical analysis was determined by one-way ANOVA and Tukey's test. ^∗^p < 0.05, ^∗∗^p < 0.01, ^∗∗∗^p < 0.001.

Herein, we addressed our main aim to assess the potential application of donor-type iPS-DCregs in the prevention of allograft rejection. We intravenously injected 1 × 10^6^ B6 iPS-DCregs or other B6-derived DCs (BM-DCcons, iPS-DCcons, and BM-DCregs) into CBA recipients 7 days prior to receiving a B6 heart allograft in the absence of IS drug therapy. The MST of pretreatment with BM-DCcons (n = 8, MST 8 days) and iPS-DCcons (n = 9, MST 8 days) was almost the same as that of the PBS control group. Conversely, administration of BM-DCregs significantly prolonged the survival of cardiac allografts as previously described. Notably, immunization of iPS-DCregs resulted in permanent acceptance of the allografts (n = 19, MST >100 days) (Figure 3A). We noticed that administration of the same number of BM-DCregs and iPS-DCregs induced the protective effects of different allografts. We thought that this might be caused by the difference in purity (CD11b^+^CD11c^+^%) between BM-DCregs (mean ± SD: 54.2% ± 3.9%) and iPS-DCregs (81.4% ± 2.2%) (Figure S1B). Next, we reduced the dose of iPS-DCregs to address this hypothesis. The MST of the half-dose (5 × 10^5^) group was 26 days (n = 7), which prolonged allograft survival but did not lead to permanent acceptance (Figure S3B).

Histology showed serious lymphocyte infiltration around the coronary arteries in PBS-treated grafts on postoperative day 7 (POD7). In contrast, lymphocyte infiltration in the iPS-DCregs-immunized grafts was markedly reduced (Figure 3B).

### iPS-DCregs Reduced CTLs and Downregulated Proinflammatory Cytokine

The infiltration of CD8^+^bromodeoxyuridine^+^ (BrdU^+^) T cells in allografts was significantly decreased in the iPS-DCregs pretreatment group compared with the PBS group on POD7, and was further reduced on POD14 (Figure 3C). We checked the splenocytes from recipients and found that CD8^+^ T cells (%) were also significantly reduced in the iPS-DCregs pretreatment group, which was consistent with the allograft assessment (Figure 3D).

As cytotoxic T lymphocyte (CTL)-induced cell death is initiated by the Perforin/Granzyme B pathway (Hayashida et al., 2000), we detected the mRNA expression of Perforin/Granzyme B in allografts. Logically, the levels of both Perforin and Granzyme B were lower in the grafts from iPS-DCregs-treated recipients compared with the grafts from the non-treatment group. Also, other proinflammatory cytokines, such as tumor necrosis factor α (TNF-α), interleukin-1β (IL-1β), IL-6, inducible nitric oxide synthase, and HO-1, were downregulated in the grafts from iPS-DCregs-treated recipients compared with those from non-treatment recipients, except IFN-γ (Figure 3E).

### Tregs Play a Key Role in Acceptance of iPS-DCregs-Induced Allografts

**Figure 4:**
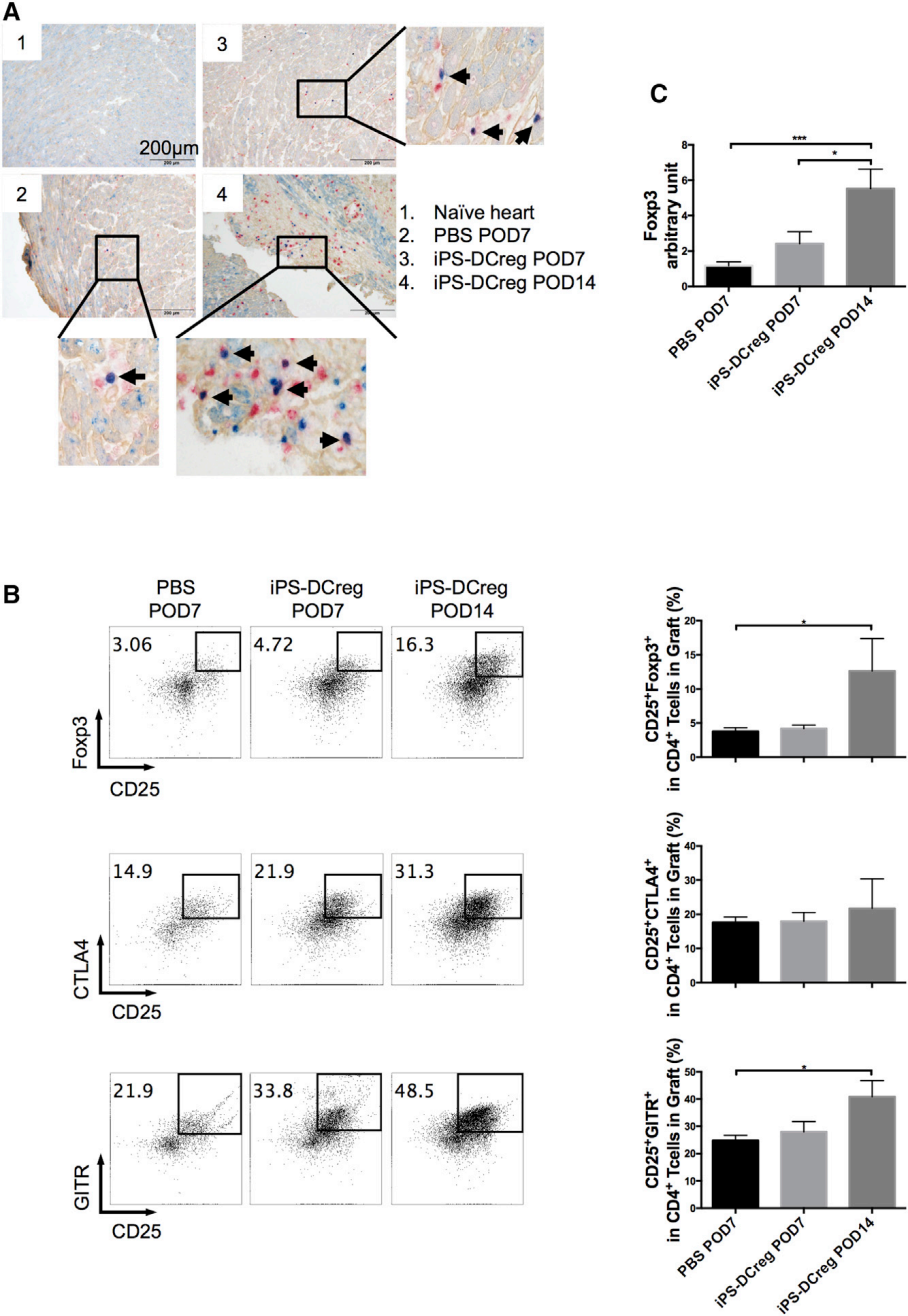
iPS-DCreg Immunization Increases Activated Tregs in Allografts (A) Allografts were harvested on POD7 and POD14, and were stained with anti-FOXP3 (blue), collagen IV (yellowish-brown), and BrdU (red). The FOXP3^+^BrdU^+^ cells are shown in purple (indicated by the black arrows). Naive B6 hearts served as the control. (B) The infiltrating lymphocytes in the grafts were separated and triple stained for CD4/CD25/FOXP3 or CD4/CD25/CTLA-4 or CD4/CD25/GITR (n = 5 in PBS-POD7 group, n = 3 in iPS-DCregs POD7 and iPS-DCregs POD14 group; mean ± SD, pooled from three independent experiments). Statistical analysis was determined by one-way ANOVA and Tukey's test. ^∗^p < 0.05. (C) The mRNA expression of FOXP3 in allografts harvested on POD7 and POD14 was detected by qRT-PCR (n = 9 in PBS-POD7 group, n = 6 in iPS-DCregs POD7 group, n = 5 in iPS-DCregs POD14 group; mean ± SD, pooled from five independent experiments). Statistical analysis was determined by one-way ANOVA and Tukey's test. ^∗^p < 0.05, ^∗∗∗^p < 0.001.

Tregs markedly increased in allografts from the iPS-DCregs-immunized group in both immunohistochemistry and FCM assessments (Figures 4A and 4B [upper]). We continuously detected the activity markers of Tregs, CTLA-4, and GITR. CTLA-4 is a critical regulator of T cell responses as a co-stimulation (CD28/CD80-86) blocker and through other pathways (Hou et al., 2015, Krummey and Ford, 2014, Soskic et al., 2014). GITR (glucocorticoid-induced TNF receptor-related protein) appears to be a marker of activated Tregs and is widely used in functional studies on Tregs (Hilchey et al., 2007, Ronchetti et al., 2015). We found that in addition to the increased number of Tregs, the expression of CTLA-4 and GITR was significantly increased in the allografts from the iPS-DCregs-immunized group (Figure 4B, middle and lower). The spleen acts like a control tower in this process. The increase of activated Tregs in the spleen is totally consistent with the allograft (Figure S4).

**Figure 5:**
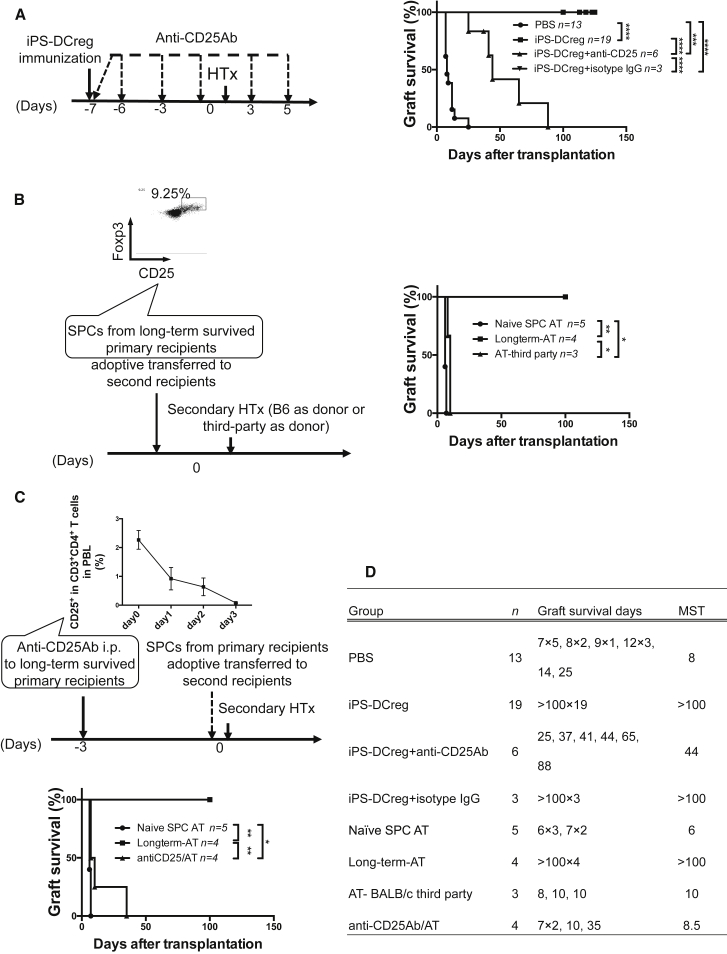
Tregs Generated by iPS-DCreg Immunization Are Donor Specific and Play a Key Role in Tolerance Induction and Maintenance (A) CBA recipient mice were treated with B6 iPS-DCregs and anti-CD25 mAb. A statistical evaluation of graft survival was performed using Kaplan-Meier curves and compared using log-rank tests. ^∗∗∗^p < 0.001, ^∗∗∗∗^p < 0.0001. (B) SPCs harvested from iPS-DCregs-treated recipients (primary recipients) on POD100, which included 9.25% Tregs in the CD4^+^ population, were adoptively transferred to naive CBA (secondary recipients) at 5 × 10^7^, and B6 hearts or BALB/c hearts (third party) were transplanted into the second recipients. A statistical evaluation of graft survival was performed using Kaplan-Meier curves and compared using log-rank tests. ^∗^p < 0.05, ^∗∗^p < 0.01. (C) Anti-CD25 mAb was injected into the primary recipients on POD97. The depletion of CD25^+^ cells was monitored in peripheral blood (PBL) by FCM. SPCs were then isolated from the primary recipients on POD100 and adoptively transferred to naive CBA (secondary recipients) at 5 × 10^7^ just before the second transplantation. A statistical evaluation of graft survival was performed using Kaplan-Meier curves and compared using log-rank tests. ^∗^p < 0.05, ^∗∗^p < 0.01. (D) Graft survival data in this figure are presented in detail.

We demonstrated that Tregs played a key role in allograft acceptance induced by iPS-DCregs, and the protective effects were donor specific in the following three tests. First, recipients were treated with iPS-DCregs at 1 × 10^6^ (full dose) 7 days prior to transplantation, then anti-CD25 monoclonal antibody (mAb) (clone: PC61) (1 mg/mouse) was injected into the peritoneum to deplete Tregs (Couper et al., 2009, Hirai et al., 2016, Liu et al., 2012, Miller et al., 2015, Setiady et al., 2010). Tregs depletion broke the permanent acceptance of allografts induced by iPS-DCregs (n = 6, MST 44 days) (Figures 5A and 5D). Second, adoptive transfer (AT) of splenocytes from long-term surviving recipients (>POD100) into naive CBA (secondary recipients) led to B6-derived allograft acceptance (n = 4, MST >100 days) while third-party (BALB/c)-derived allografts were rejected (n = 3, MST 10 days). This indicated that the protective effect was donor specific (Figures 5B and 5D). Lastly, we injected anti-CD25 mAb into long-term surviving recipients. Three days later, we isolated their SPCs and adoptively transferred them to naive CBA (secondary recipients). This treatment reversed the AT-induced allograft acceptance (n = 4, MST 8.5 days) (Figures 5C and 5D), which indicated that donor-specific Tregs played an essential role in the maintenance phase of donor-specific tolerance.

### TGF-β1 Blockage Interrupts the Protective Effect Induced by iPS-DCregs

TGF-β1 is one of the primary cytokines in immunosuppression. We therefore examined the role of TGF-β1 in allograft acceptance induced by iPS-DCregs.

**Figure 6:**
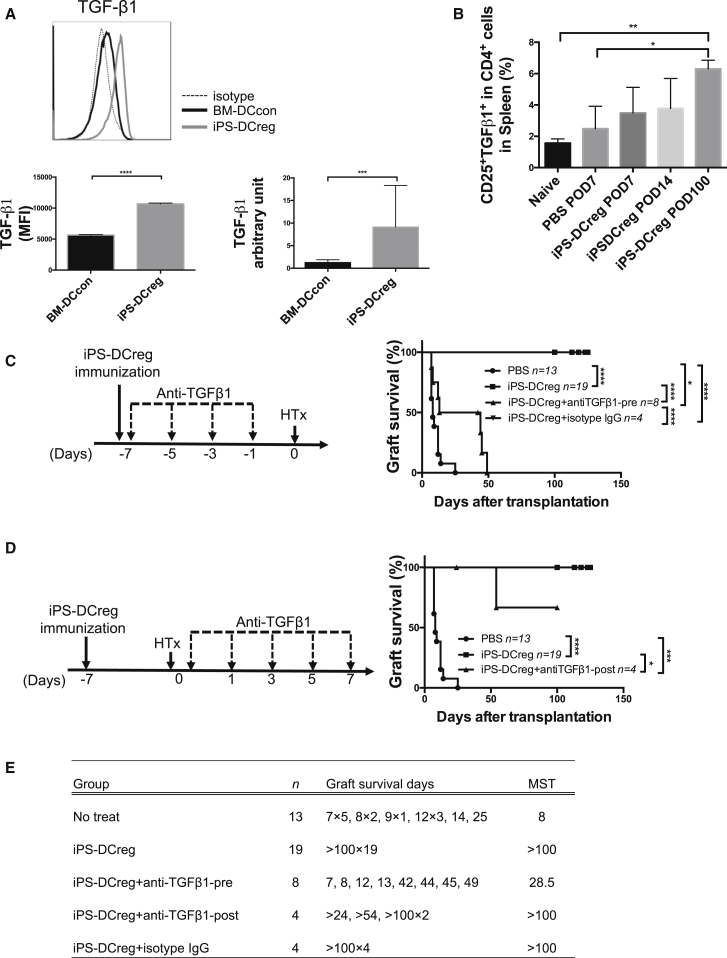
TGF-β1 Is More Essential in the Primary Vaccination than in the Secondary Immunization (A) The bar graph shows the TGF-β1 expression in the CD11b^+^CD11c^+^ population and the mean fluorescence intensity (MFI) is calculated (n = 3 in each group, mean ± SD, pooled from three independent experiments). The mRNA expression of TGF-β1 was measured (n = 5 in each group, mean ± SD, pooled from five independent experiments). Statistical analysis was determined by Student's t test. ^∗∗∗^p < 0.001, ^∗∗∗∗^p < 0.0001. (B) SPCs were harvested on POD7, POD14, and POD100, and triple stained for CD4/CD25/TGF-β1 for FCM. The quantitation is the percentage of CD25^+^TGF-β1^+^ cells in the CD4^+^ cell population (n = 4 in POD100 group, n = 3 each in other groups; mean ± SD, pooled from three independent experiments). Statistical analysis was determined by one-way ANOVA and Tukey's test. ^∗^p < 0.05, ^∗∗^p < 0.01. (C) Anti-TGF-β1mAb was injected into the iPS-DCregs immunized CBA (recipient) before heart transplantation. A statistical evaluation of graft survival was performed using Kaplan-Meier curves and compared using log rank tests. ^∗^p < 0.05, ^∗∗∗∗^p < 0.0001. (D) Anti-TGF-β1 mAb was injected into the iPS-DCregs immunized CBA (recipient) after heart transplantation. A statistical evaluation of graft survival was performed using Kaplan-Meier curves and compared using log rank tests. ^∗^p < 0.05, ^∗∗∗^p < 0.001, ^∗∗∗∗^p < 0.0001. (E) Graft survival data in this figure is presented in detail.

First, as shown previously, the mRNA expression of TGF-β1 of iPS-DCregs was significantly higher compared with BM-DCcons (Figure 6A, right; Zhang et al., 2014). Second, the TGF-β1 molecule expression in the CD11b^+^CD11c^+^ population of iPS-DCregs was significantly higher compared with BM-DCcons (Figure 6A, left). Third, membrane-bound TGF-β1 in Tregs was significantly increased in the SPCs isolated from the iPS-DCregs-treated group (Figure 6B). This evidence reminded us that TGF-β1 must play a critical function in the protective effect induced by iPS-DCregs. We then demonstrated this through the following tests with anti-TGF-β1 mAb.

First, recipients were treated with iPS-DCregs at 1 × 10^6^ on day −7 and anti-TGF-β1 mAb (400 μg/mouse intraperitoneally) on days −7, −5, −3, and 1 to block TGF-β1 before transplantation. This treatment prevented the permanent acceptance of allografts (n = 8, MST 28.5 days) (Figures 6C and 6E).

Second, recipients were treated with iPS-DCregs at 1 × 10^6^ on day −7 and then with anti-TGF-β1 mAb (400 μg/mouse intraperitoneally) on days 0, 1, 3, 5, and 7 to block TGF-β1 after transplantation. However, the blockage of TGF-β1 after transplantation did not impede the allograft acceptance induced by iPS-DCregs (n = 4, MST >100 days) (Figures 6D and 6E).

**Figure 7:**
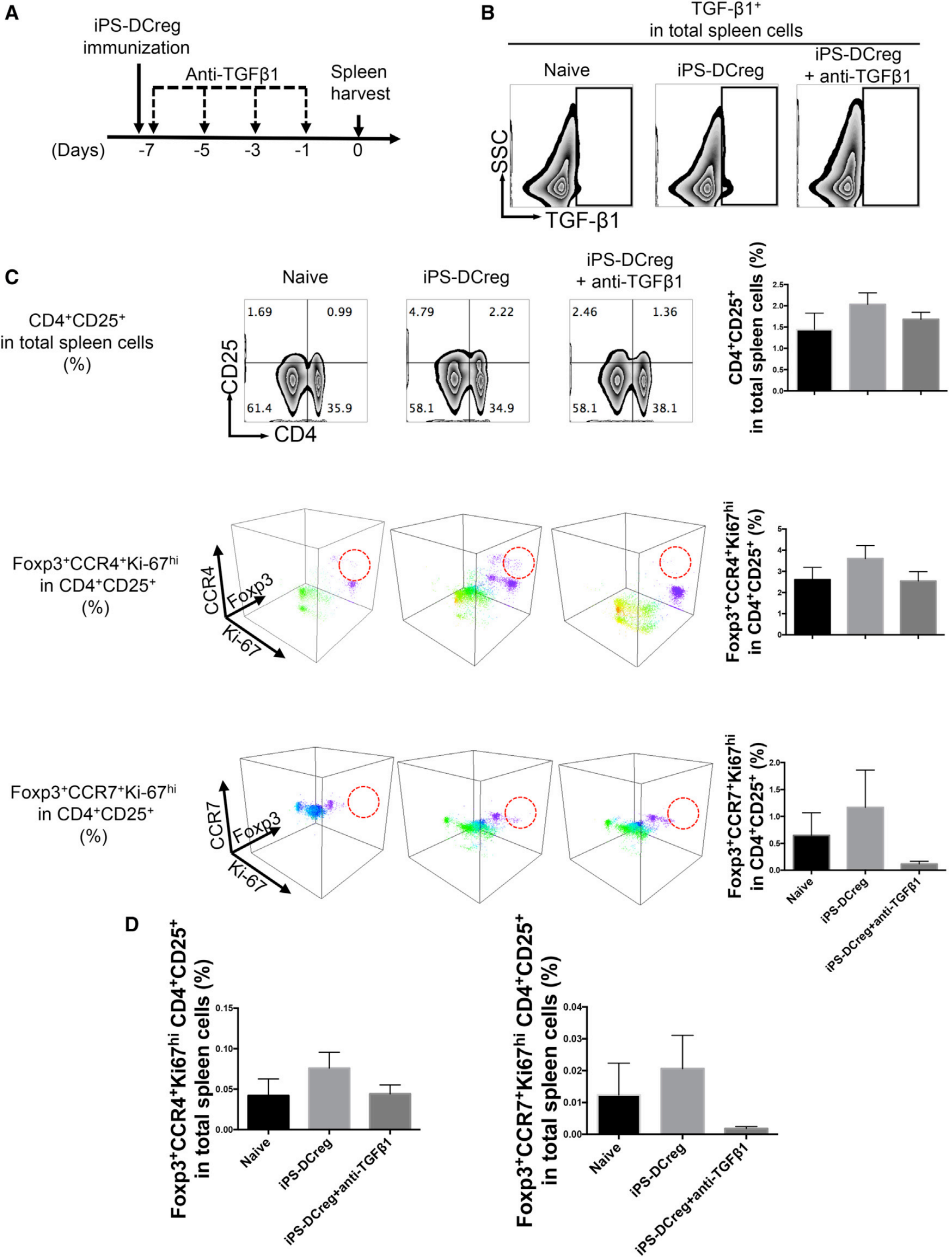
FOXP3^+^CCR4^+^Ki-67^hi^ and FOXP3^+^CCR7^+^Ki-67^hi^ Tregs Are Increased by iPS-DCreg Immunization in a TGF-β1-Dependent Pattern (A) Anti-TGF-β1 mAb was injected into the iPS-DCregs-treated CBA (the same protocol as shown in Figure 6C), and SPCs were harvested. (B) TGF-β1 blockade was identified by FCM. (C) SPCs were multiply stained with CCR4/Ki-67/CD4/CD25/FOXP3 or CCR4/Ki-67/CD4/CD25/FOXP3 for FCM. The expression of CCR4/Ki-67/FOXP3 and CCR4/Ki-67/FOXP3 in the CD4^+^ FOXP3^+^ population is presented in a 3D visualization (n = 3 each in other groups, mean ± SD, pooled from three independent experiments). Statistical analysis was determined by one-way ANOVA and Tukey's test. No statistically significant difference was observed between these groups. (D) The quantitation is the percentage of FOXP3^+^CCR4^+^(or CCR7^+^) Ki-67^hi^CD4^+^CD25^+^ cells in total SPCs (n = 3 each in other groups, mean ± SD, pooled from three independent experiments). Statistical analysis was determined by one-way ANOVA and Tukey's test. No statistically significant difference was observed between these groups.

Finally, CBA mice were treated with B6 iPS-DCregs at 1 × 10^6^ on day −7 and anti-TGF-β1 mAb (400 μg/mouse, intraperitoneally) on days −7, −5, −3, and 1 to block TGF-β1. SPCs were harvested on day 0 for FCM (Figure 7A). iPS-DCregs immunization enhanced TGF-β1 expression in SPCs, while anti-TGF-β1 mAb clearly blocked TGF-β1 (Figure 7B). We then scored how the TGF-β1 blockade changed the proportion and character of Tregs induced by iPS-DCregs. Firstly, we demonstrated that the number of CD4^+^CD25^+^ cells in SPCs was increased by iPS-DCregs immunization while anti-TGF-β1 blocked this effect (Figure 7C, upper). Furthermore, we detected the expression of FOXP3, Ki-67, CCR4, and CCR7 in CD4^+^CD25^+^ cells, which were widely used to gauge the activity and transmigration ability of Tregs (Sugiyama et al., 2013, Zhang et al., 2009a). The percentage of FOXP3^+^CCR4^+^Ki-67^hi^ and FOXP3^+^CCR7^+^Ki-67^hi^ in CD4^+^CD25^+^ cells was increased by iPS-DCregs immunization but was decreased by TGF-β1 blockage (Figure 7C-middle and lower). We then calculated the percentage of CD4^+^CD25^+^ FOXP3^+^CCR4^+^Ki-67^hi^ and CD4^+^CD25^+^FOXP3^+^CCR7^+^Ki-67^hi^ in the total spleen. Also, iPS-DCregs immunization increased the percentage of these two populations in SPCs (Figure 7D). These results indicated that iPS-DCregs immunization not only increased the number of Tregs but also enhanced the activity and transmigration capability of Tregs, which worked in a TGF-β1-dependent manner.

## Discussion

Donor-specific tolerance that does not compromise the overall immune response is the ultimate goal in the transplantation field. DCregs-based therapies could potentially promote donor-specific tolerance to prevent allograft rejection and graft-versus-host disease (Bonham et al., 2002, DePaz et al., 2003, Garrovillo et al., 2001, Lan et al., 2006, Lutz et al., 2000, Min et al., 2000, Mirenda et al., 2004, Morelli and Thomson, 2007, Sato et al., 2003b, Turnquist et al., 2007, Zhang et al., 2008). Both donor-type DCregs (Bonham et al., 2002, DePaz et al., 2003, Fu et al., 1996, Lan et al., 2006, Lu et al., 1997, Lutz et al., 2000, Min et al., 2000, Taner et al., 2005, Zhang et al., 2008) and recipient-type DCregs (loaded with donor antigen) (Ali et al., 2000, Beriou et al., 2005, Garrovillo et al., 1999, Oluwole et al., 2001, Peche et al., 2005, Sato et al., 2003b) were reported to be able to prolong allograft survival through different pathways (direct, indirect, semi-direct). However, most of these studies are based on murine/rat bone marrow stem cells and human blood mononuclear cell-derived DCs, which require a large number of progenitor cells. Also, the quantity and quality of cultured DCs were inconsistent because of different ages, health conditions, and other variables among the sample. Recently, Kudo et al. (2014) differentiated donor-type macrophage-like IS cells from mouse ESCs, and these cells were found to prolong allograft survival. However, the ethical issues and patient-matched limitations of ESCs prevented them from being used in the clinical setting. In our previous study, we successfully differentiated DCregs from iPSCs (Zhang et al., 2014). The present study addressed the hypothesis of whether the administration of donor-type iPS-DCregs was capable of generating donor-specific tolerance.

Several conclusions can be drawn from the current study. First, iPS-DCregs not only indicate a high purity of CD11b^+^CD11c^+^ cells, but also retain a stable “immature” phenotype, even in the presence of strong maturational stimulus, IFN-γ. Many groups treated recipients with at least 2 × 10^6^ bone marrow-derived DCregs, immature DCs, or other suppressive cells (such as myeloid-deprived suppressor cells) to achieve prolonged allograft survival (Arakawa et al., 2014, Fu et al., 1996, Rastellini et al., 1995, Tiao et al., 2005). In this study, 1 × 10^6^ iPS-DCregs induced permanent allograft acceptance while BM-DCregs did not. The co-stimulator (CD40, CD80, CD86) and MHC-II molecule expressions in the CD11b^+^CD11c^+^ population were not significantly different between BM-DCregs and iPS-DCregs. However, iPS-DCregs have a significantly higher percentage of CD11c^+^ in the CD11b^+^ population compared with BM-DCregs. We thought that the high CD11b^+^CD11c^+^ purity of iPS-DCregs was the reason why the same dose of BM-DCregs and iPS-DCregs led to a different outcome. The number of administered CD11b^+^CD11c^+^ cells is known to directly affect the tolerance-inducing reaction (Morelli and Thomson, 2007). Recipients treated with a half-dose of iPS-DCregs indicated a graft survival similar to that with a full-dose of BM-DCregs, which was identified in our hypothesis. Our findings suggest that the addition of iPS-DCregs into MLR culture and PLN significantly suppressed the T cell proliferative response. These characteristics engineered iPS-DCregs as important “regulatory cellular vaccines” in the allogeneic transplantation model (Morelli and Thomson, 2007, Sato et al., 2003a, Sato et al., 2003b, Thomson et al., 2009).

Second, donor-type iPS-DCregs activated donor-specific Tregs. Donor-type DCregs expressing donor MHC molecules traveled to the recipient's secondary lymphoid tissues and interacted with T cells through the “direct pathway” of allorecognition. The direct pathway of allorecognition is considered to be the most powerful mechanism to instigate early acute graft rejection (Morelli and Thomson, 2007). Figuratively speaking, pretreatment of donor-type iPS-DCregs acted as an immune suppressive vaccine, which led to a primary immune response. According to the “two-signal” hypothesis of T cell activation (Mueller et al., 1989), recipient naive T cells interacted with allogeneic MHC molecules on DCregs through a direct pathway, followed by the delivery of potent signal 1 plus poor signal 2 by DCregs to naive T cells. This resulted in the generation of donor-specific Tregs (Bakdash et al., 2013) and anergy of donor-specific Teffs (Sato et al., 2003a). On the other hand, Tiao et al. (2005) pretreated recipients with recipient-type immature BM-DCs pulsing with donor antigens, which prolonged the allograft MST by 40 days. However, in our study, recipient-type BM-DCregs with or without pulsing with donor antigens could not protect allografts from acute rejection (MST 7 days). In Tiao et al.’s (2005) study, 2 × 10^6^ immature BM-DCs were intravenously injected into recipients, while we only used 1 × 10^6^ BM-DCregs. The different DC dose may be the reason why recipient-type DCregs treatment did not work in our preliminary study.

Third, Tregs generated by iPS-DCregs were vital in allograft tolerance (Joffre et al., 2008, Kitazawa et al., 2007, Sakaguchi, 2004, Zhang et al., 2009b), especially in the maintenance phase (1–3 months post operation). Interestingly, donor-type iPS-DCregs served as the “primary vaccination,” which “prepared” the inhibited immune situation for the allografts. Based on this analogy, the alloantigen loaded by allografts served as the “secondary vaccination.” We hypothesized that after transplantation, the alloantigen interacted with the Tregs and precursor Tregs induced by the primary vaccination and then further activated alloantigen-specific Tregs. The number of Tregs and the expression of CTLA-4 in Tregs were significantly higher on POD100 compared with POD7 (Figure S4), which indicated that donor-specific Tregs expanded and activated unceasingly (Schubert et al., 2014, Wing et al., 2008). This may have caused the alloantigen loaded on the allograft to work as a stimulator to promote the clonal expansion of donor-specific Tregs, which we referred to as the secondary vaccination.

Fourth, IFN-γ, a key inflammatory cytokine produced by Teffs, was higher in the iPS-DCregs-treated group than in the non-treated group on POD7, but became lower on POD14. Some groups reported that IFN-γ knockout (KO) and IFN-γ receptor (IFN-γR) KO recipients rejected allografts much more quickly compared with wild-type, because IFN-γ plays a crucial role in Teff apoptosis through several pathways (Morita et al., 2015, Ring et al., 1999). Thus, we speculate that IFN-γ is essential in DCregs-induced tolerance via Teff apoptosis.

Fifth, TGF-β1 was certainly required in the “primary vaccination” but was not essential for the early stage of “secondary vaccination.” We have revealed here that the blockage of TGF-β1 during the period between iPS-DCregs treatment and allotransplantation prevented iPS-DCregs-induced allotolerance. However, the blockage of TGF-β1 post transplantation could not break the tolerance. Early studies demonstrated that TGF-β1 could generate Tregs through several pathways, for example, the inhibition of IL-2, the upregulation of cyclin-dependent kinase (CDK) inhibitors (p15, p21, and p27), and the downregulation of cell-cycle-promoting factors (c-myc, cyclin D2, CDK2, and cyclin E) (Wan and Flavell, 2007). We identified that the blockage of TGF-β1 not only decreased the number of Tregs induced by iPS-DCregs (CD4^+^CD25^+^%), but also downregulated the activity and transmigration ability of Tregs (CD4^+^CD25^+^FOXP3^+^CCR4^+^Ki-67^hi^% and CD4^+^CD25^+^FOXP3^+^CCR7^+^Ki-67^hi^%). The protective effects of Tregs on allograft survival were abrogated if they failed to migrate to the graft due to CCR4 and CCR7 deficiency. Logistically, the protection was enhanced when Tregs were delivered locally into the grafts (Sugiyama et al., 2013, Zhang et al., 2009a). We believe that this is the reason why the blockage of TGF-β1 during the period between iPS-DCregs immunization and allotransplantation could prevent iPS-DCregs-induced allotolerance. Although our data showed that donor-type iPS-DCregs treatment induced donor-specific Tregs by upregulating TGF-β1, it remains possible that these Tregs and TGF-β1 may participate in non-specific immune suppression. Nonetheless, although our examination indicated that Tregs and TGF-β1 are two key factors in allotolerance induced by iPS-DCregs, there could be other mechanisms involved in this therapy. In addition, further research is needed before this therapy can be adapted for clinical application, including dose, timing, dosage, and/or combination with low-dose IS drugs.

In summary, we have successfully generated alloantigen-specific Tregs with therapeutic activity toward allorejection by infusion of donor-type iPS-DCregs. Although there are still many barriers to be overcome, we believe that iPS-DCregs offer a potentially efficient and reliable approach for use in transplantation and/or autoimmune diseases.

## Experimental Procedures

### Study Design

Our primary research objective was to establish a method to generate donor antigen-specific Tregs in vivo in recipient mice by donor-type iPS-DCregs immunization and to search its core mechanism. The overall study design was a series of controlled laboratory experiments as indicated in the sections below.

In vivo experimental groups included 3–10 mice per group, with two exceptions. First, because an AT study requires many recipients' spleens on POD100 (endpoint), the graft survival data of the iPS-DCregs-treated group included 19 mice. Second, because most assessments needed to use the PBS group as a negative control, the grafts survival data of the PBS group included 13 mice. Mice were randomly assigned to each group, but the researchers were not blinded to the group identity.

### Animals

Male CBA/N (CBA; H-2k^k^), C57BL/6 (B6; H-2k^b^), and BALB/c (H-2k^d^) mice were purchased from the Shizuoka Laboratory Animal Center. C57BL/6-Tg (TCR-OT-I) Cbn (OT-I, H-2k^b^) and C57BL/6-Tg (TCR-OT-II) Cbn (OT-II, H-2k^b^) transgenic mice were kindly supplied by Dr. N. Ishii (Graduate School of Medicine, Tohoku University) and Dr. S. Nakae (The Institute of Medical Science, The University of Tokyo), respectively. All mice were bred and maintained under standard conditions and fed rodent food and water according to the guidelines of the Animal Use and Care Committee of the National Research Institute for Child Health and Development, Tokyo, Japan. All animal experiments were approved by this committee and performed according to its recommendations.

### Tregs and TGF-β1 Blockage

Recipients were treated with anti-CD25 mAb (cat. #BE0012, clone PC61.5.3, BioXCell) (Hirata et al., 2007, Couper et al., 2009) or anti-TGF-β1 mAb (cat. #BE0057, clone 1D11.16.8, BioXCell) (Kasagi et al., 2014) by intraperitoneal administration. The schematics of the protocol are shown in the associated results and figures.

### Additional Methods

Information regarding DC culture, heterotopic cardiac transplantation, MLR, PLNA, graft infiltration lymphocyte isolation, flow cytometry, histopathology, immunohistochemistry, and qRT-PCR is provided in Supplemental Experimental Procedures.

### Statistical Analyses

The data were analyzed using GraphPad Prism, version 6.0 (GraphPad Software). One-way ANOVA and Tukey's test were used to compare the means of more than two groups. Student's t test was used to compare the means of two groups. A statistical evaluation of graft survival was performed using Kaplan-Meier curves and compared using log-rank tests. All in vitro experimental data were representative of at least three independent experiments. p Values of less than 0.05 were considered statistically significant.

## Author Contributions

S.C., X.-K.L., P.Z., J.-M.C., and J.Z. conceived and designed the experiments. S.C., J.H., Q.Z., and R.A. performed the experiments. S.C., J.H., Q.Z., M.F., N.I., S.T., L.L., P.Z., J.-M.C., and J.Z. analyzed the data. S.C., R.A., and L.L. contributed reagents/materials/analysis tools. S.C., M.F., and X.-K.L. wrote the paper.

## Supplemental Information

Document S1. Supplemental Experimental Procedures, Figures S1–S5, and Table S1

Document S2. Article plus Supplemental Information
