# Supplementary material for: iPSC-Derived Regulatory Dendritic Cells Inhibit Allograft Rejection by Generating Alloantigen-Specific Regulatory T Cells
Source: Stem Cell Reports. 2017 Apr 20;8(5):1174–89. doi: 10.1016/j.stemcr.2017.03.020 (PMC5425686; doi:10.1016/j.stemcr.2017.03.020)
Supplement: Document S1. Supplemental Experimental Procedures, Figures S1–S5, and Table S1 [file mmc1.pdf]

Stem Cell Reports, Volume *8*

Supplemental Information

iPSC-Derived Regulatory Dendritic Cells Inhibit Allograft Rejection by Generating Alloantigen-Specific Regulatory T Cells

Songjie Cai, Jiangang Hou, Masayuki Fujino, Qi Zhang, Naotsugu Ichimaru, Shiro Takahara, Ryoko Araki, Lina Lu, Ji-Mei Chen, Jian Zhuang, Ping Zhu, and Xiao-Kang Li

Supplementary Information

Supplemental Experimental Procedures:

DC culture, related to main-text Result 1

We induced the differentiation of iPS cells and the B6 embryonic fibroblast-derived iPS cell line (2A-4F-100, H-2^b)(Araki et al., 2013) in DCregs mainly according to the protocol based on the method by Senju et al. (Senju et al., 2009). Steps 1 and 2 were described previously (Zhang et al., 2014), while step 3 was modified in this study as follows (**fig. S1, A**). For step 3, the cells at the end of step 2 were cultured in a 24-well hydrocells plate (CellSeed Inc., Tokyo, Japan) as $5x10^5$ per well in 1ml RPMI-1640 medium (Life Technologies, Gaithersburg, MD) supplemented with antibiotics, 10% (vol./vol.) fetal calf serum (Gibco, Carlsbad, CA) and 50mper well in 1ml RPMI-1640 medium (Life Tec the complete medium). iPS-DCcons were in the presence of GM-CSF (20ng/ml), IL-4 (10ng/ml). iPS-DCregs were in the presence of GM-CSF (20ng/ml), TGF-β1 (20ng/ml), IL-10 (20ng/ml) (PeproTech, London, UK). On day 5 of step 3, the cells were stimulated with IFN-γ (10ng/ml) for 48 hours and harvested on day 7. The iPS-DCcons used in this

study contained >87.4% $CD11b^+CD11c^+$ cells, while the iPS-DCregs used in this study contained >81.4% $CD11b^+CD11c^+$ cells.

Bone marrow cells (BMCs) were collected from the femoral and tibial of B6 or BALB/c. For the generation of BM-DCcons, BMCs were cultured in a 24-well TC plate (Greiner bio-one, Tokyo, Japan) as 1×10^6 per well with the complete medium in the presence of GM-CSF (10ng/ml), IL-4 (10ng/ml). IFN-γ (10ng/ml) was added at day 5 with two additional days for maturation. For the generation of BM-DCregs, BMCs were cultured in a 24-well TC plate as 1×10^6 per well with the complete medium in the presence of GM-CSF (20ng/ml), TGF-β1 (20ng/ml) and IL-10 (10ng/ml). IFN-γ (10n g/ml) was added on day 5 with two additional days for maturation.

Heterotopic cardiac transplantation, related to figure 3, 5, 6

All transplant procedures were performed under anesthesia with isoflurane. Fully vascularized heterotopic hearts from B6 or BALB/c were transplanted into CBA recipients using a microsurgical technique (Cai et al., 2016). The beats of grafts were manipulated

daily for the first two weeks and twice weekly for three months after transplantation. Graft survival was considered complete at the time of cessation of a palpable heart beating, and confirmed visually by laparotomy.

Isolation of lymphocytes from grafts, related to figure 4

Grafts were collected and cut into 1-2mm pieces on ice. Then the tissue was disrupted mechanically in 10ml digestion solution, which include 0.5mg/ml collagenase IV (Sigma), 50U/ml DNaseI (Invitrogen) in Phosphate buffered saline (PBS) (Gibco), and incubated at 37 °C for 20 minutes. After that, 10ml iced RPMI 1640 with 5% fetal calf serum was added. The suspension was filtered through a nylon mesh (100 μm) to remove aggregates. The resulted cell suspension was centrifuged at 200 × g for 10 minutes to pellet the cells. The pellet was suspended in 5ml PBS, loaded onto 5ml Lympholyte-M (Cedarlane, Canada) and centrifuged at 1500 × g for 25 minutes at room temperature. Cells were isolated from the Lympholyte-M interface and washed twice in PBS at 300× g for 5 minutes and prepared for FCM assay.

Mixed leukocyte reactions (MLR), related to figure 1, 2

One-way MLR culture was performed in duplicate in 96-well, round-bottom plates (Greiner bio-one). Nylon wool-eluted spleen T cells (2×10^5 /well) were labeled with CellTrace Violet (Life Technologies) and used as responders. Cultures were maintained in the complete medium for 3-5 days in 5% CO_2 in air. The reaction system and other details are shown in the associated figure legends.

Popliteal lymph node (PLN) assay, related to figure 1

Splenic T cells were isolated from OT-I and OT-II mice using a nylon-wool column and labeled with Violet (OT-I) or CFSE (Life Technologies) (OT-II). Prepared OT-I or OT-II T cells were adoptively transferred to naïve B6 mice at 3×10^6 /500ml/mice at day -1. DCs were pulsed with OVA as 400 mg/ml for 24 hours and then were injected subcutaneously into the recipients' footpads at day 0. The negative control mice were

treated with PBS. The PLN were harvested at day 3. The reaction system and other details are shown in the associated figure legends.

Flow cytometry, related to all figures,

Expression of DCs surface antigens was analyzed by Attune acoustic focusing cytometer (Applied Biosystems, Carlsbad, CA). Cells were stained with CD11b-PE/Cy7 (Cat. 101216; negative control: PE/Cy7 Rat IgG2b, κ Isotype Ctrl, Cat. 400618; Biolegend, San Diego, CA), CD11c-APC (Cat. 117310; negative control: APC Armenian Hamster IgG Isotype Ctrl, Cat. 400912; Biolegend), and FITC-conjugated CD40 (Cat. 124608; negative control: FITC Rat IgG2a, κ Isotype Ctrl, Cat. 400506; Biolegend), CD80 (Cat. 104706; negative control: FITC Armenian Hamster IgG Isotype Ctrl, Cat. 400906; Biolegend), CD86 (Cat. 105110; negative control: FITC Rat IgG2b, κ Isotype Ctrl, Cat. 400606; Biolegend), IA-IE (Cat. 107606; negative control: FITC Rat IgG2b, κ Isotype Ctrl, Cat. 400605; Biolegend). Antigen uptake test of DCs was analyzed by Attune (Applied Biosystems). Cells were stained with CD11b-PE/Cy7, CD11c-APC, and FITC-conjugated

OVA (Cat. O23020, Lifetechnologies, Carlsbad, CA) or Dextran (Cat. D1844, Lifetechnologies). SPCs isolated from recipients after heart transplantation were stained with CD4-Pacific orange (Cat. MCD0430; negative control: Pacific orange Rat IgG2a Isotype Control, Cat. R2a30; Lifetechnologies), CD25-APC (Cat. 102012; negative control: APC Rat IgG1, λ Isotype Ctrl, Cat. 401904; Biolegend), CTLA4-PE (Cat. 106306; negative control: PE Armenian Hamster IgG Isotype Ctrl, Cat. 400908; Biolegend), GITR-PE (Cat. 126310; negative control: PE Rat IgG2b, κ Isotype Ctrl, Cat. 400608; Biolegend), TGF-β1-PE (Cat. 141404; negative control: PE Mouse IgG1, κ Isotype Ctrl, Cat. 400112; Biolegend), CD8α-PE/Cy7 (Cat. 100722; negative control: PE/Cy7 Rat IgG2a, κ Isotype Ctrl, Cat. 400522; Biolegend) and FOXP3-PE (Cat. 12-5773-82; negative control: PE Rat IgG2a K Isotype Ctrl, Cat. 12-4321-42; eBioscience, Santa Clara, CA). GILs were stained with CD4-FITC (Cat. 100510; negative control: FITC Rat IgG2a, κ Isotype Ctrl; Biolegend) or CD4-APC (Cat. 100516; negative control: APC Rat IgG2a, κ Isotype Ctrl, Cat. 400512, Biolegend), CD25-APC/Cy7 (Cat. 102026, Biolegend; negative control: APC/Cy7 Rat IgG1, λ Isotype Ctrl, Cat. 557663; BDBiosciences), CTLA4-APC (Cat.

106310; negative control: APC Armenian Hamster IgG Isotype Ctrl; Biolegend), GITR-PE, CD8α-PE/Cy7 and FOXP3-PE. For FOXP3 staining, Transcription Factor Fixation/Permeabilization Concentrate and Diluent (eBioscience) was used. SPCs were analyzed by Gallios flow cytometer (Beckman Coulter, Brea, CA) and GILs were analyzed by Attune. Cells from MLR were stained with CD4-APC and CD8α-PE/Cy7; and were detected by Gallios. SPCs isolated from CBA, which only accepted iPS-DCregs and anti-TGFβ1 mAb but without heart transplantation, were stained with: 1) TGF-β1-PE; 2) CD4-FITC, CD25-APC/Cy7, Ki67-PE/Cy7 (Cat. 652426; negative control: PE/Cy7 Rat IgG2a, κ Isotype Ctrl; Biolegend), CCR4-APC (Cat. 131212; negative control: APC Armenian Hamster IgG Isotype Ctrl; Biolegend) and FOXP3-PE; and 3) CD4-APC, CD25-APC/Cy7, Ki67-FITC (Cat. 652410; negative control: FITC Rat IgG2a, κ Isotype Ctrl; Biolegend), CCR7-PE/Cy7 (Cat. 120124; negative control: PE/Cy7 Rat IgG2a, κ Isotype Ctrl; Biolegend) and FOXP3-PE. This test was detected by Attune. All flow cytometric data were analyzed with FLOWJO V.10.0.8 (FLOWJO LLC, Ashland, OR).

Histopathology, related to figure 3

Grafts were harvested on POD7 and POD14 were fixed in 10% formalin solution (WAKO, Osaka, Japan) and then embedded in paraffin. Sections of 4μm were made for hematoxylin and eosin (HE) staining.

Immunohistochemistry, related to figure 3, 4

Grafts and spleens were harvested on POD7 and POD14. 5-bromo-2-deoxyuridine (BrdU) was injected to the recipients 1 hour before sampling. Cryo-blocks were immediately frozen through Hexane (WAKO) in -80 °C. Sections of 4 μm were cut for immunohistochemistry staining. Triple immunostaining was performed. The rat anti-mouse CD4 mAb (Cat. 100506, Biolegend), rat anti-mouse CD8α mAb (Cat. 100802, Biolegend) and anti-mouse FOXP3 biotin mAb (Cat. 13-5773-82, eBioscience) were used as the primary antibodies. After being rehydrated, sections were blocked with 4% block ace (Dainippon Sumitomo, Osaka, Japan) for 10 minutes. Primary antibodies were used as a 1:100 dilution, and sections were incubated in primary antibody working solution at room

temperature for 1 hour and then washed by PBS. Secondary antibody: ALP-conjugated donkey anti-rat IgG (Cat. 712-005-150 , Jackson Immuno Research, West Grove, PA) for rat anti-mouse CD4 mAb, rat anti-mouse CD8α mAb incubated samples and ALP-conjugated anti-biotin antibody (Cat. A6561, Sigma-Aldrich, St. Louis, MO) for anti-mouse FOXP3 biotin mAb incubated samples. Secondary antibodies were used as 1:100 dilution and heat-inactivated normal mouse serum were added to working solution as 1% final concentration. Sections were incubated in secondary antibody working solution at room temperature for 1 hour. Labeled proteins were visualized with Vector-Blue kit (VECTOR Laboratories, Burlingame, UK) according to the manufacturer's instructions. Thereafter, cryo-sections were incubated with rabbit-anti-mouse type IV collagen polyclonal Ab (Cat. LB1403, Cosmo Bio, Tokyo, Japan) diluted as 1:3000 for 1 hour, and then incubated with HRP-conjugated goat anti-rabbit Ig (Cat. 08674191, MP Biomedicals, Santa Ana, CA) as 1:100 and developed with DAB Peroxidase Substrate Kit (Vector Laboratories). Subsequently, the samples were incubated at 37 °C with pepsin-HCl solution for 12 minutes then treated with 4N.HCl for 30 minutes at room temperate and neutralized

with borax buffer (0.1 M; pH 8.5) for 4 minutes. BrdU was then detected with the rat anti-BrdU mAb (Cat. OBT0030CX, AbD Serotec, Raleigh, NC) followed by the ALP-conjugated donkey anti-rat Ig (Jackson Immuno Research). Sections were visualized with New Fuchsin Substrate System (Dako, Carpinteria, CA). Finally, sections were fixed in FCa solution (4% Paraformaldehyde /PBS +1%$CaCl_2$) for 10 minutes and then mounted in Aquatex (Merck, Whitehouse Station, NJ).

RNA Preparation and Quantitative Reverse Transcriptase-polymerase Chain Reaction (qRT-PCR), related to figure 3, 4, 6

Cardiac grafts were harvested on POD7 and POD14 and submerged in RNAlater® stabilization solution (Life Technologies, Carlsbad, CA) for freezing. Total RNA was extracted from frozen tissue samples using ISOGEN (NipponGene, Tokyo, Japan). DCs were directly frozen at -80 °C and total RNA was extracted by RNeasy Mini Kit (QIAGEN, Limburg, Nederland). Then, RNA was reverse transcribed to cDNA using PrimeScript® RT reagent Kit (Takara Bio, Shiga, Japan). Quantitative RT-PCR was performed using a

TaqMan system on the Applied Biosystems PRISM7700 instrument (Applied Biosystems). The normalized threshold cycle (Ct) value of each gene was obtained by subtracting the Ct value of 18S rRNA. The sequences used in our study are shown in **Table S1**.

Adoptive transfer study, related to figure 5

Splenocytes (5×10^7) isolated from the primary recipients bearing accepted B6 cardiac allografts after iPS-DCregs therapy on POD100 were adoptively transferred into naïve CBA mice (secondary recipients) on day -1. Then, the secondary recipients underwent transplantation of B6 or BALB/c (third party) cardiac grafts on day 0. The schematics of the protocol are shown in the associated figures.

Supplementary Figure:

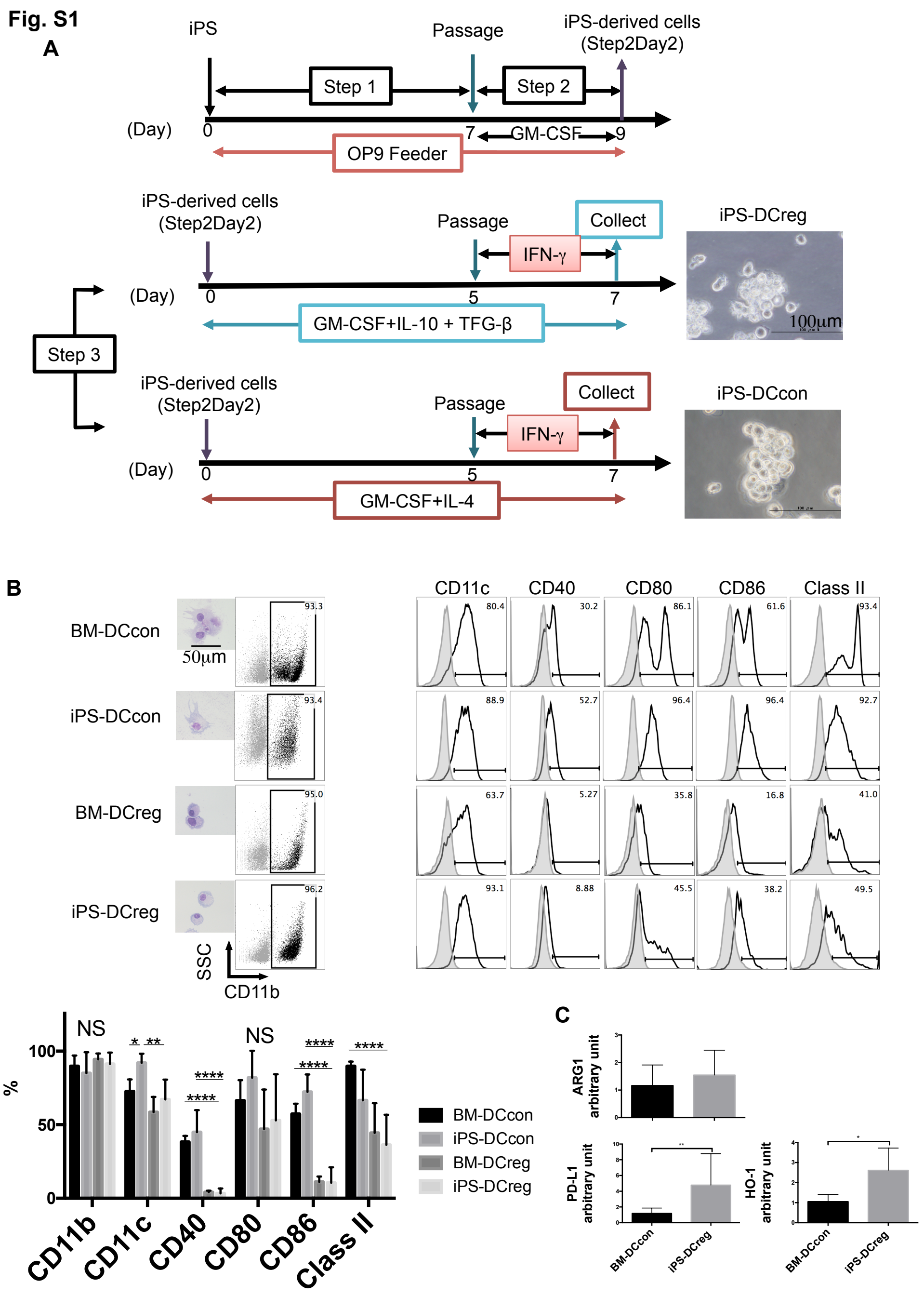

Fig. S1. *Generation protocol and phenotype of iPS-DCregs, related to main-text Experimental Procedures and Result 1.* (**A**) Generation protocol of iPS-DCs is shown in schema. (**B**) The morphology of DCs is shown as May-Grunwald and Giemsa staining (original magnification×400). The percentage of $CD11b^{+}$ is shown as dot plot. The expression of CD11c, co-stimulatory molecules and MHC-II antigen is displayed as histograms gated on the $CD11b^{+}$ cell population. Grey lines show isotype control. (*n*=9 in BM-DCcon, *n*=5 in iPS-DCcon, *n*=6 in BM-DCreg, *n*=8 in iPS-DCreg, mean ± SD). Statistical analysis was determined by one-way ANOVA and Tukey's test. *p<0.05, **p<0.01, ****p<0.0001. (**C**) The mRNA expression of ARG1, PD-L1, HO-1 was detected by qRT-PCR (*n*=5 in each group, mean ± SD). Statistical analysis was determined by Student's t test. *p<0.05, **p<0.01.

Fig. S2

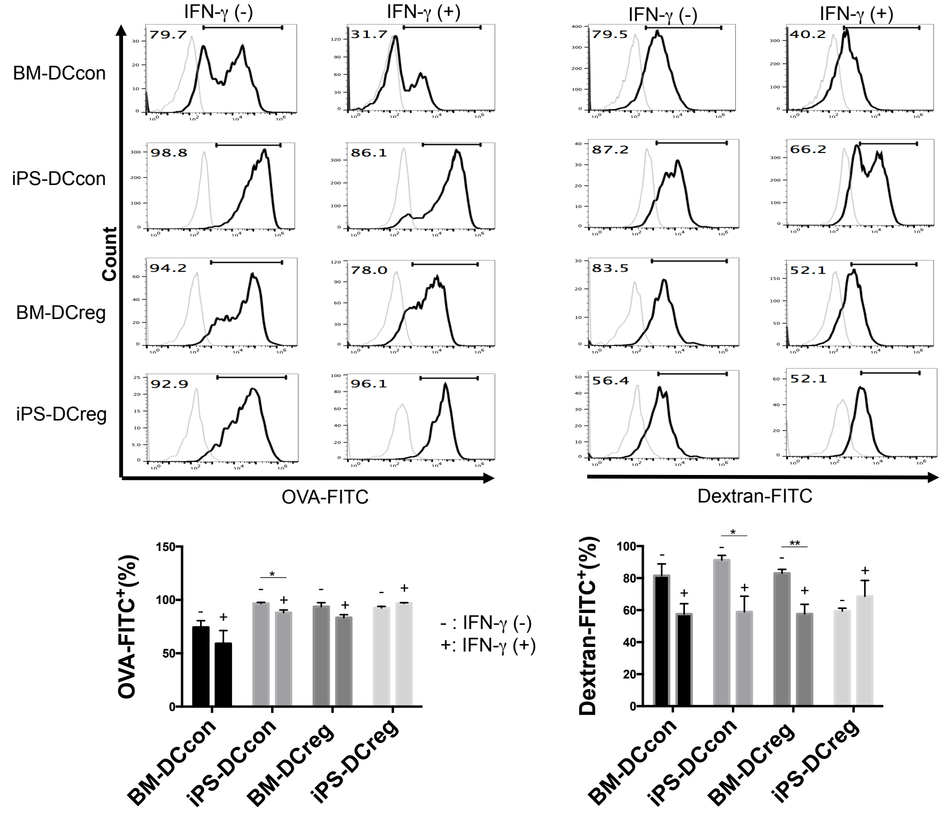

Fig. S2. *Antigen uptake ability test of iPS-DCregs before and after IFN-γ stimulation, related to main-text Result 1.* DCs (**fig. S1, A**) were harvested on day 5 (without IFN-γ stimulation) and day 7 (48-hr IFN-γ stimulation) for protein antigen (OVA-FITC) and carbohydrate antigen (Dextran-FITC) uptake analysis (*n*=4 in each group, mean ± SD).

Statistical analysis was determined by one-way ANOVA and Tukey's test. $^{*}p<0.05$, $^{**}p<0.01$.

Fig. S3

A

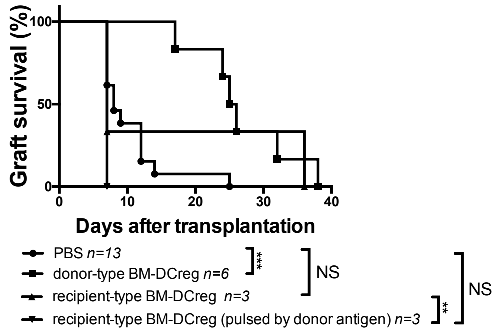

Group	*n*	Graft survival days	MST
PBS	13	7×5, 8×2, 9×1, 12×3, 14, 25	8
donor-type BM-DCreg	6	17, 24, 25, 26, 32, 38	25.5
recipient-type BM-DCreg	3	7×2, 36	7
recipient-type BM-DCreg (pulsed by donor antigen)	3	7×3	7

B

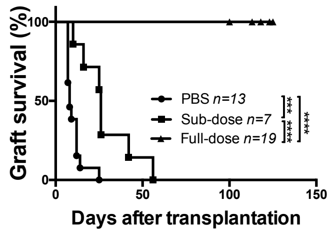

Group	*n*	Graft survival days	MST
PBS	13	7×5, 8×2, 9×1, 12×3, 14, 25	8
iPS-DCreg Sub-dose	7	7 10, 16, 25, 26×2, 42, 56	26
iPS-DCreg Full-dose	19	>100×19	>100

Fig. S3. *Different origins and different doses of DCregs lead to different allografts protection effect, related to main-text Result 4.* (**A**) 1×10^6 CBA (recipient-type) derived

BM-DCregs (with or without donor-antigen pulsed) or B6 (donor-type) derived BM-DCregs were i.v. into CBA (recipient) mice 7 days before heterotopic cardiac transplantation. A statistical evaluation of the graft survival was performed using the Kaplan-Meier curves and compared using the Log-Rank tests. ****$p<0.001$*. (**B**) 1×10^6 (full-dose) or 5×10^5 (sub-dose) B6 (donor-type) derived iPS-DCregs were i.v. into CBA (recipient) mice 7 days before heterotopic cardiac transplantation. A statistical evaluation of the graft survival was performed using the Kaplan-Meier curves and compared using the Log-Rank tests. ****$p<0.001$*, *****$p<0.0001$*.

Fig. S4

A

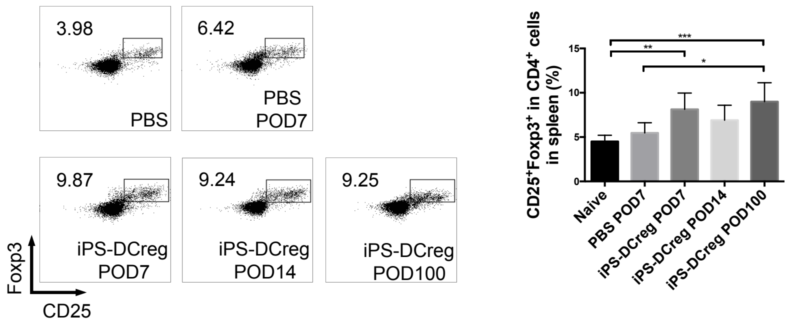
3.98
PBS
6.42
PBS
POD7
9.87
iPS-DCreg
POD7
9.24
iPS-DCreg
POD14
9.25
iPS-DCreg
POD100
Foxp3
CD25
CD25+Foxp3+ in CD4+ cells
in spleen (%)
Naive
PBS POD7
iPS-DCreg POD7
iPS-DCreg POD14
iPS-DCreg POD100

B

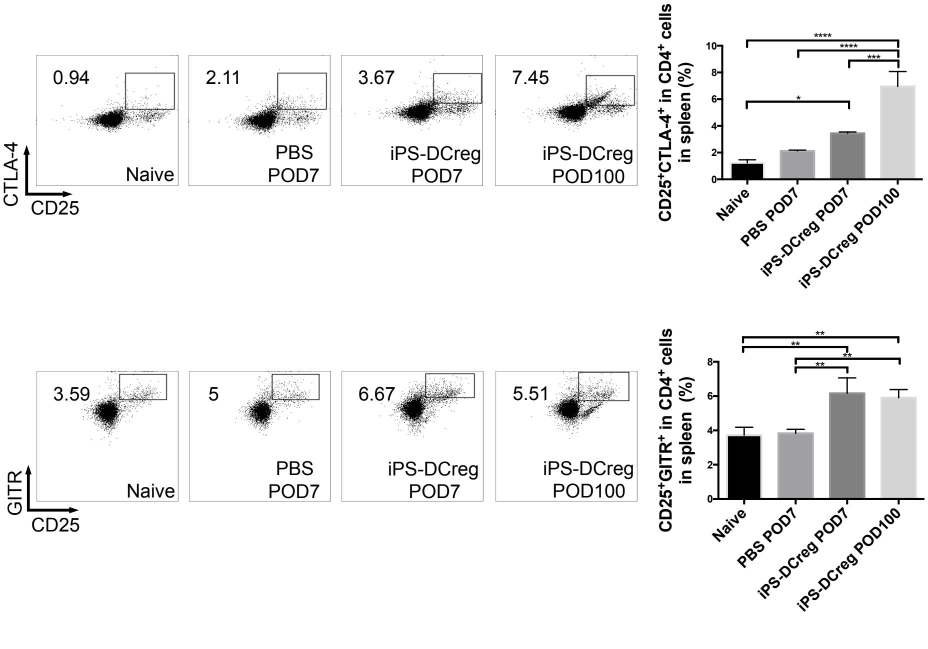
0.94
Naive
2.11
PBS
POD7
3.67
iPS-DCreg
POD7
7.45
iPS-DCreg
POD100
CTLA-4
CD25
CD25+CTLA-4+ in CD4+ cells
in spleen (%)
Naive
PBS POD7
iPS-DCreg POD7
iPS-DCreg POD100
3.59
Naive
5
PBS
POD7
6.67
iPS-DCreg
POD7
5.51
iPS-DCreg
POD100
GITR
CD25
CD25+GITR+ in CD4+ cells
in spleen (%)
Naive
PBS POD7
iPS-DCreg POD7
iPS-DCreg POD100

Fig. S4. *Activated Tregs expanded in spleen from iPS-DCregs immunized recipients after allo-transplantation, related to main-text Result 6.* (**A**) Spleens were harvested on POD7, 14 and 100, and were triple stained for CD4/CD25/FOXP3 (n=7 in naïve control, n=5 in PBS-POD7, n=6 in iPS-DCregs-POD7, n=4 in iPS-DCregs-POD14 and POD100, mean ± SD). Statistical analysis was determined by one-way ANOVA and Tukey's test. *$p<0.05$, **$p<0.01$, ***$p<0.001$. (**B**) Spleens were harvested on POD7 and 100, and were triple stained for CD4/CD25/CTLA-4 or CD4/CD25/GITR (n=3 in naïve control, PBS-POD7 and iPS-DCregs-POD7, n=4 in iPS-DCregs-POD100, mean ± SD). Statistical analysis was determined by one-way ANOVA and Tukey's test. *$p<0.05$, **$p<0.01$, ***$p<0.001$, ***$p<0.0001$.

Fig. S5

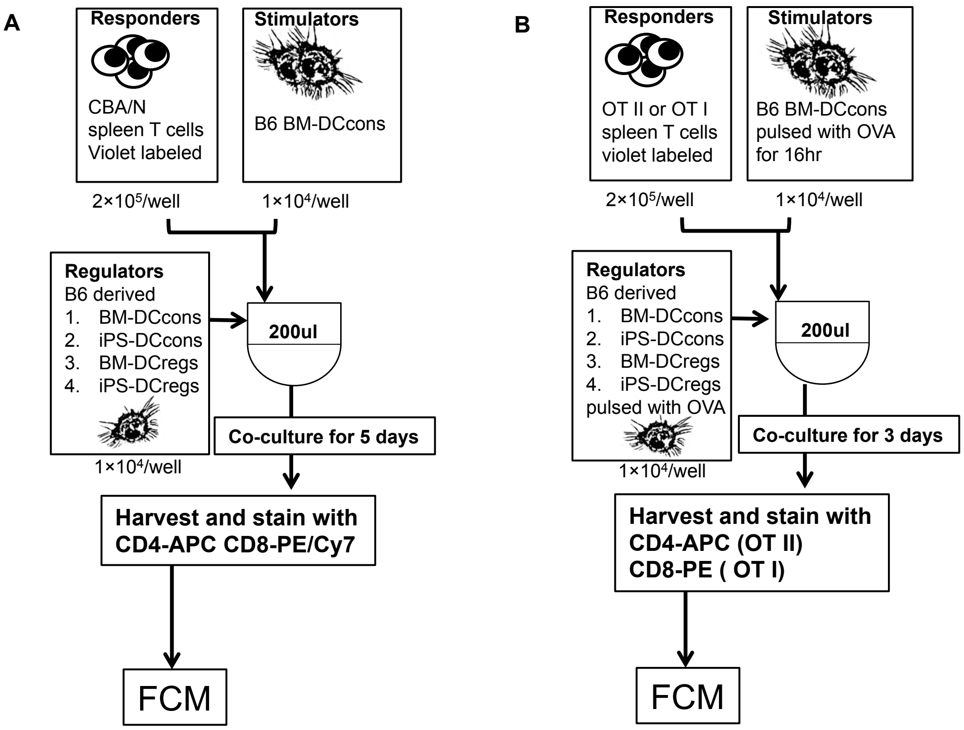

Fig. S5. *MLR reactive system used in this study, related to main-text figure 1, 2*. (**A**) Violet-labeled CBA T cells (responder) were cultured with B6 BM-DCcons (stimulator) at a ratio of 20:1 for 5 days. Four types of DCs were added at the beginning into the culture as a regulator (regulator: stimulator 1:1). Proliferation of T cells was determined by Violet dilution gated on $CD4^+$ and $CD8^+$ population. (**B**) Violet-labeled OT-II or OT-I T cells (responder) were cultured with OVA-pulsed B6 BM-DCcons (stimulator) at a ratio of 20:1

for 3 days. Four types of DCs were added into the culture as regulators (regulator: stimulator 1:1). Proliferation of T cells was determined by Violet dilution gated on $CD4^+$ (OT-II) or $CD8^+$ (OT-I) populations.

Table S1. Primer Sets Used in This Study, Related to Figure 3, 4, 6

Genes	Forward Primer	Reverse Primer	Probe
**IL-6**	5' CTgCAAgTgCATCATCgTTgT 3'	5' TgTCTATACCACTTCACAAgTCggA 3'	5' CAgAATTgCCATTgCACAACTCTTTTCTCA 3
**IL-1β**	5' TgAAAgACggCACACCCA 3'	5' gACAAACCgCTTTTCCATCTTC 3'	5' CAgCTggAgAgTgTggATCCCAAACA 3'
**Granzyme B**	5' AgAgTggggCTTgACTTCATgT 3'	5' TCCTgCTACTgCTgACCTTgTCT 3'	5' CCCgATgATCTCCCCTgCCTTTgT 3'
**Perforin**	5' AAgTCAAggTggAgTggAggTTT 3'	5' gCTgAgAAgACCTATCAggACCA 3'	5' CCAggCgAAAACTgTACATgCgACAC 3'
**HO-1**	5' CAgggTgACAgAAgAggCTAAgAC 3'	5' TTgTgTTCCTCTgTCAgCATCAC 3'	5' TCCTgCTCAACATTgAgCTgTTTgAggA 3'
**iNOS**	5' CAgTggAgAgATTTTgCATgACA 3'	5' CCCCAAgCAAgACTTggACTT 3'	5' CCACAAggCCACATCggATTTCACTT 3'
**TNF-α**	5' TgTCTACTgAACTTCggggTgAT 3'	5' AACTgATgAgAgggAggCCAT 3'	5' TCCCCAAAgggATgAgAAgTTCCCAA 3'
**Arg-1**	5' TgCTgCAgggCCTTTCTC 3'	5' gAgCTCCAAgCCAAAgTCCTT 3'	5' CCTCCTCgAggCTgTCCTTTTgAgAAAg 3'
**TGF-β1**	5' AggTCACCCgCgTgCTAA 3'	5' gCTTCCCgAATgTCTgACgTA 3'	5' TggACCgCAACAACgCCATCTATg 3'

**IFN-γ**	5' CAAggCgAAAAAgg ATgCA 3'	5' CggATgAgCTCATTgA ATgCT 3'	5' TgCCAAgTTTgAggTCAA CAACCCACA 3'
**FOXP3**	5' gCCCACCAgTACAg CTggA 3'	5' CAAgTCTCgTCTgAAg gCAgAgT 3'	5' CCACTCCAgCTCCCggC AACTTC 3'
**PD-L1**	5' AgCCAgggCAAAAC CACA 3'	5' AgCACCCAgTgAgTCC TgTTCT 3'	5' TgATCATCCCAgAACTg CCTgCAACA 3'
**18S**	5' ATgAgTCCACTTTA AATCCTTTAACgA 3'	5' CTTTAATATACgCTA TTggAgCTggAA 3'	5' ATCCATTggAgggCAAgT CTggTgC 3'

Supplementary Reference

Araki, R., Uda, M., Hoki, Y., Sunayama, M., Nakamura, M., Ando, S., Sugiura, M., Ideno, H., Shimada, A., Nifuji, A., *et al.* (2013). Negligible immunogenicity of terminally differentiated cells derived from induced pluripotent or embryonic stem cells. Nature *494*, 100-104.

Cai, S., Ichimaru, N., Zhao, M., Fujino, M., Ito, H., Ota, U., Nakajima, M., Tanaka, T., Nonomura, N., Li, X.K., *et al.* (2016). Prolonged Mouse Cardiac Graft Cold Storage via Attenuating Ischemia-Reperfusion Injury Using a New Antioxidant-Based Preservation Solution. Transplantation *100*, 1032-1040.

Senju, S., Haruta, M., Matsunaga, Y., Fukushima, S., Ikeda, T., Takahashi, K., Okita, K., Yamanaka, S., and Nishimura, Y. (2009). Characterization of dendritic cells and macrophages generated by directed differentiation from mouse induced pluripotent stem cells. Stem cells *27*, 1021-1031.

Zhang, Q., Fujino, M., Iwasaki, S., Hirano, H., Cai, S., Kitajima, Y., Xu, J., and Li, X.K. (2014). Generation and characterization of regulatory dendritic cells derived from murine

induced pluripotent stem cells. Scientific reports *4*, 3979.
