# Supplementary material for: iPSC-Derived Regulatory Dendritic Cells Inhibit Allograft Rejection by Generating Alloantigen-Specific Regulatory T Cells
Source: Stem Cell Reports. 2017 Apr 20;8(5):1174–89. doi: 10.1016/j.stemcr.2017.03.020 (PMC5425686; doi:10.1016/j.stemcr.2017.03.020)
Supplement: Document S2. Article plus Supplemental Information [file mmc2.pdf]

Stem Cell Reports
Article

OPEN ACCESS

iPSC-Derived Regulatory Dendritic Cells Inhibit Allograft Rejection by Generating Alloantigen-Specific Regulatory T Cells

Songjie Cai,[1,2,8] Jiangang Hou,[1,3,8] Masayuki Fujino,[1,4] Qi Zhang,[3] Naotsugu Ichimaru,[2] Shiro Takahara,[2] Ryoko Araki,[5] Lina Lu,[6] Ji-Mei Chen,[7] Jian Zhuang,[7] Ping Zhu,[7,*] and Xiao-Kang Li[1,*]
[1]Division of Transplantation Immunology, National Research Institute for Child Health and Development, 2-10-1 Okura, Setagaya-ku, Tokyo 157-8535, Japan
[2]Department of Advanced Technology for Transplantation, Osaka University Graduate School of Medicine, Osaka 565-0871, Japan
[3]Huashan Hospital, Fudan University, Shanghai 200032, China
[4]AIDS Research Center, National Institute of Infectious Diseases, Tokyo 162-8640, Japan
[5]Department of Basic Medical Sciences for Radiation Damages, National Institute of Radiological Sciences, Chiba 263-8555, Japan
[6]Department of Immunology, Lerner Research Institute, Cleveland Clinic, Cleveland, OH 44195, USA
[7]Department of Cardiac Surgery, Guangdong Cardiovascular Institute, Guangdong General Hospital, Guangdong Academy of Medical Sciences, Guangzhou 510100, China
[8]Co-first author
*Correspondence: tanganqier@163.com (P.Z.), ri-k@ncchd.go.jp (X.-K.L.)
http://dx.doi.org/10.1016/j.stemcr.2017.03.020

SUMMARY

Regulatory dendritic cell (DCregs)-based immunotherapy is a potential therapeutic tool for transplant rejection. We generated DCregs from murine induced pluripotent stem cells (iPSCs), which could remain in a "stable immature stage" even under strong stimulation. Harnessing this characteristic, we hypothesized that iPS-DCregs worked as a negative vaccine to generate regulatory T cells (Tregs), and induced donor-specific allograft acceptance. We immunized naive CBA (H-2K^{k}) mice with B6 (H-2K^{b}) iPS-DCregs and found that Tregs (CD4^{+}CD25^{+}FOXP3^{+}) significantly increased in CBA splenocytes. Moreover, immunized CBA recipients permanently accepted B6 cardiac grafts in a donor-specific pattern. We demonstrated mechanistically that donor-type iPS-DCregs triggered transforming growth factor β1 secretion, under which the donor-antigen peptides directed naive CD4^{+} T cells to differentiate into donor-specific FOXP3^{+} Tregs instead of into effector T cells in vivo. These findings highlight the potential of iPS-DCregs as a key cell therapy resource in clinical transplantation.

INTRODUCTION

The main form of therapy for allograft rejection is immunosuppressive (IS) drugs. Unfortunately, non-specific immunosuppression often causes numerous adverse side effects, such as opportunistic infection and cancer (Dantal et al., 1998), and also fails to induce antigen-specific tolerance. Thus, reducing the use of IS drugs and inducing donor-specific tolerance are the main objectives in transplantation.

Regulatory immune cell therapy, including regulatory T cells (Tregs) (Bradley, 2014; McMurchy et al., 2011), regulatory dendritic cells (DCregs) (Ezzelarab and Thomson, 2011; Moreau et al., 2012), and immature DCs (iDCs) (Roncarolo et al., 2001), is an emerging strategy for the prevention of allograft rejection by promoting antigen-specific tolerance and the elimination of IS drug use (Raich-Regue et al., 2014; Wood et al., 2012). Because DCregs play essential roles in maintaining immune homeostasis (Morelli and Thomson, 2007), they are usually the target of rejection treatment. However, the lack of stable therapeutic DCregs has been the biggest problem in clinical application. Induced pluripotent stem cells (iPSCs), created by Yamanaka and colleagues in 2006, can propagate indefinitely and differentiate into various cells just like embryonic stem cells (ESCs) (Takahashi et al., 2007; Takahashi and Yamanaka, 2006). Notably, unlike ESCs, iPSCs can be generated from adult cells, which overcomes ethical issues and patient-matching limitations. In our previous study (Zhang et al., 2014), we established a novel approach for generating a sufficient quantity of high-quality functional DCregs from iPSCs (iPS-DCregs), which could be kept in a "stable immature stage" even under strong stimulation. Harnessing this characteristic, we hypothesized that donor-type iPS-DCregs expressing donor antigen worked as an immune suppressive vaccine to generate alloantigen-specific Tregs, and induced permanent acceptance of mouse cardiac allografts.

RESULTS

iPS-DCregs Are Maintained in a "Stable Immature Stage" Even under IFN-γ Stimulation

The morphology of iPS-DCregs is similar to that of bone marrow DCregs (BM-DCregs), which are smaller and have shorter dendrites. They express low levels of co-stimulatory molecules (CD40, CD80, and CD86) and major histocompatibility complex (MHC) class II antigens, and a high percentage of CD11b^{+}CD11c^{+} compared with conventional DCs (DCcons) (Hackstein and Thomson,

 | © 2017 The Author(s).
This is an open access article under the CC BY license (http://creativecommons.org/licenses/by/4.0/).

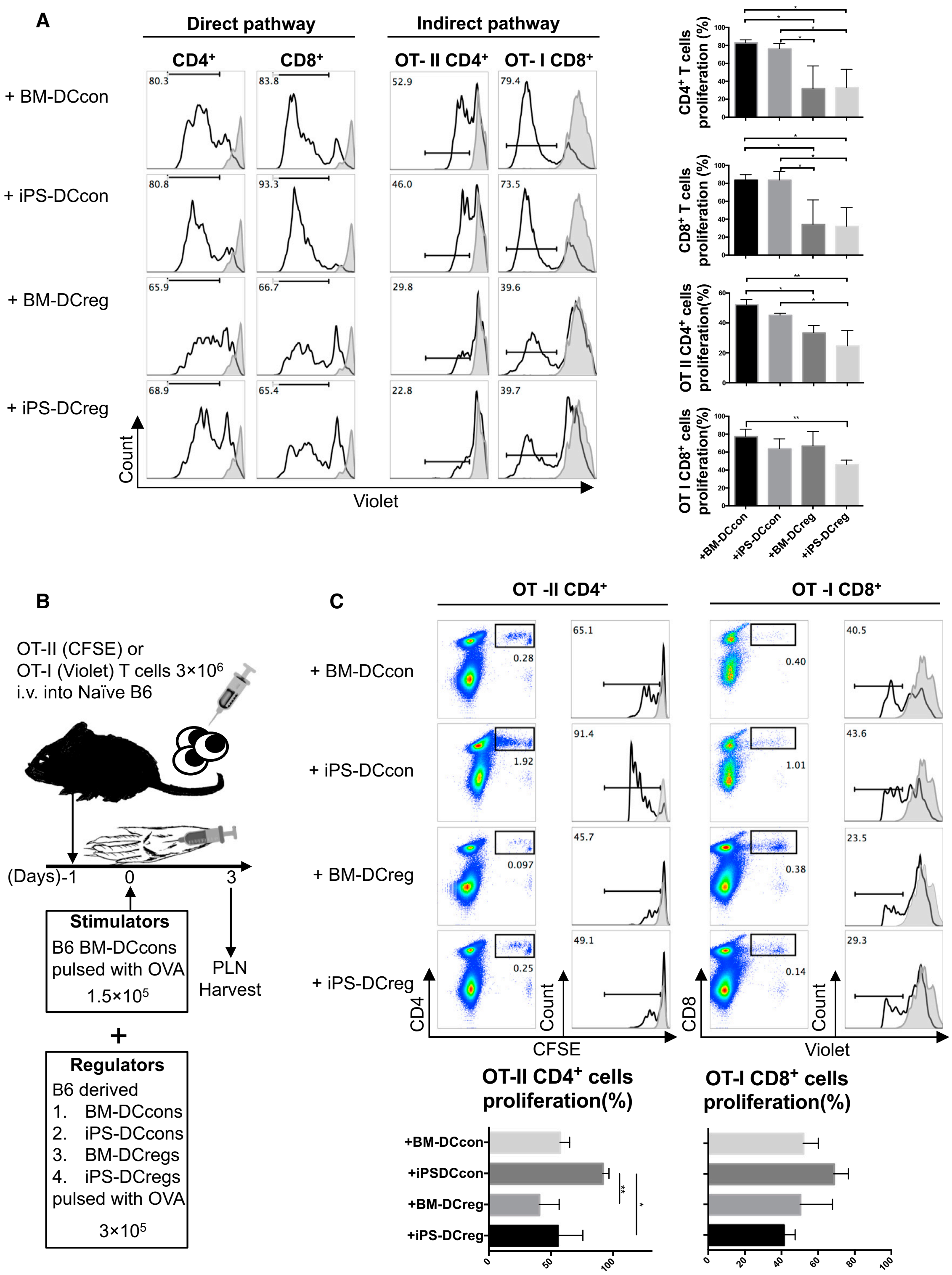

Figure 1. iPS-DCregs Suppress T Cell Proliferative Responses In Vitro and In Vivo

(A) Addition of iPS-DCregs suppresses T cell proliferation in allo-MLR (left panel) and OVA-specific MLR (right panel) (n = 3–5 in each group, mean ± SD, pooled from three independent experiments). The details of these two reaction systems are shown in Figure S5.

(legend continued on next page)

2004; Morelli and Thomson, 2003, 2007) (Figure S1B). In contrast to other DC types (BM-DCcons, iPS-DCcons, and BM-DCregs), even under interferon-γ (IFN-γ) stimulation, iPS-DCregs always maintain a high antigen uptake ability, in fluorescein isothiocyanate uptake tests with both ovalbumin (OVA) and dextran, which indicates that iPS-DCregs can be kept in a "stable immature stage" (Figure S2).

iPS-DCregs Modulate T Cell Proliferation in Direct and Indirect Pathways

We detected that mRNA expression of suppressive cytokines transforming growth factor β1 (TGF-β1), Arg-1, PD-L1, and HO-1 in iPS-DCregs was significantly higher than in BM-DCcons (Figures 6A and S1C). According to the stable immature and suppressive characteristics of the iPS-DCregs, we hypothesized that they could play a role as an immune-suppressive vaccine in allo-rejection.

To identify this role, we first set up an allogeneic mixed lymphocyte reaction (MLR) to examine the direct antigen-presenting regulatory function of iPS-DCregs (Figure S5A). naive T cells isolated from CBA mice were stimulated with B6 BM-DCcons resulting in an alloreactive proliferation. In this allogeneic MLR system, the addition of B6 BM-DCregs and iPS-DCregs significantly inhibited the proliferated response in the population of $CD4^+$ and $CD8^+$ T cells (Figure 1A, left).

We next established OVA-specific MLR to investigate the indirect antigen-presenting regulatory ability of iPS-DCregs (Figure S5B). T cells isolated from B6-background T cell receptor (TCR) transgenic mice OT-II ($CD4^+$) and OT-I ($CD8^+$) were co-cultured with OVA-pulsed B6 BM-DCcons at a 20:1 ratio for 3 days, which led to fierce OVA-reaction T cell proliferation. The addition of B6 BM-DCregs and iPS-DCregs markedly inhibited the proliferated response (Figure 1A, right).

In addition, we tested in vivo regulatory function using a proliferation lymph node assay (PLNA). Wild-type B6 mice were adoptively transferred with 3×10^6 OT-II or OT-I T cells the day before. OVA-pulsed B6 BM-DCcons (1.5×10^5) combined with iPS-DCregs or other controls (3×10^5) were injected subcutaneously into the recipients' footpads (Figure 1B). The OVA-specific reactive T cells isolated from the PLNs were intensely proliferated in the BM-DCcons and iPS-DCcons groups, but were significantly inhibited in the BM-DCregs and iPS-DCregs groups (Figure 1C).

Immunization with iPS-DCregs Generates Tregs In Vivo, Leading to Teff Suppression

Next, we immunized CBA mice with B6-derived DCs and isolated their spleens 7 days after immunization. The spleen cells (SPCs) were assessed by flow cytometry (FCM) and set up for secondary immunization in vitro (Tiao et al., 2005) (Figure 2A).

We identified that the $CD25^+FOXP3^+$ population was significantly increased in the iPS-DCregs-immunized group (Figure 2B). We then eluted T cells from these SPCs by nylon column and co-cultured them with B6 BM-DCcons or BALB/c (H-$2K^d$) BM-DCcons (third party) for MLR, which served as the secondary immunization. After 3 days of co-culture, stimulation of the BALB/c BM-DCcons aggravated the proliferation of $CD8^+$ and $CD4^+FOXP3^-$ effector T cells (Teffs) in the immunized group of T B6 iPS-DCregs compared with the negative control (CBA treated with PBS). However, the results of co-culturing with B6 BM-DCcons showed the suppression of Teff proliferation, especially in $CD8^+$ T cells (Figure 2C). This test showed that donor-type iPS-DCregs immunization directly generated Tregs in vivo, and led to donor-specific Teff suppression.

Donor-type iPS-DCregs Immunization Leads to Permanent Acceptance of Allogeneic Cardiac Grafts

Some studies have shown that recipient-type DCregs loaded with donor-antigen peptide work more efficiently than donor-type DCregs (Garrovillo et al., 1999; Ali et al., 2000). In the preliminary experiment, we tested donor-type BM-DCregs and recipient-type BM-DCregs (with or without donor-antigen pulsing). Three types of DCregs (B6 BM-DCregs, CBA BM-DCregs, and CBA BM-DCregs pulsed with H-$2K^b$ antigen peptide for 48 hr) were injected intravenously (1×10^6) into CBA recipients at 7 days prior to receiving a B6 heart allograft. We found that donor-type BM-DCregs prolonged allograft survival (PBS control n = 13, median survival time (MST) 8 days; B6 BM-DCregs n = 6, MST 25.5 days). However, neither recipient-type BM-DCregs-loaded nor recipient-type BM-DCregs-loaded donor antigen could protect allografts from acute rejection (CBA BM-DCregs n = 3, MST 7 days; CBA BM-DCregs pulsed with H-$2K^b$ antigen peptide n = 3, MST 7 days) (Figure S3A).

Herein, we addressed our main aim to assess the potential application of donor-type iPS-DCregs in the prevention of

Proliferation of T cells was determined by Violet dilution gated on the $CD4^+$ and/or $CD8^+$ population. Gray lines are T cells without BM-DCcons stimulation. Statistical analysis was determined by one-way ANOVA and Tukey's test. $^*p < 0.05$, $^{**}p < 0.01$.
(B) Schematic of PLNA protocol.
(C) Addition of iPS-DCregs suppresses T cell proliferative responses in PLNA (n = 3 in each group, mean ± SD, pooled from three independent experiments). Proliferation of T cells was determined by carboxyfluorescein diacetate succinimidyl ester (CFSE) or Violet dilution gated on $CD4^+$ (OT-II) or $CD8^+$ (OT-I) population. Gray lines indicate PBS injection into the footpad, used as a negative control. Statistical analysis was determined by one-way ANOVA and Tukey's test. $^*p < 0.05$, $^{**}p < 0.01$.

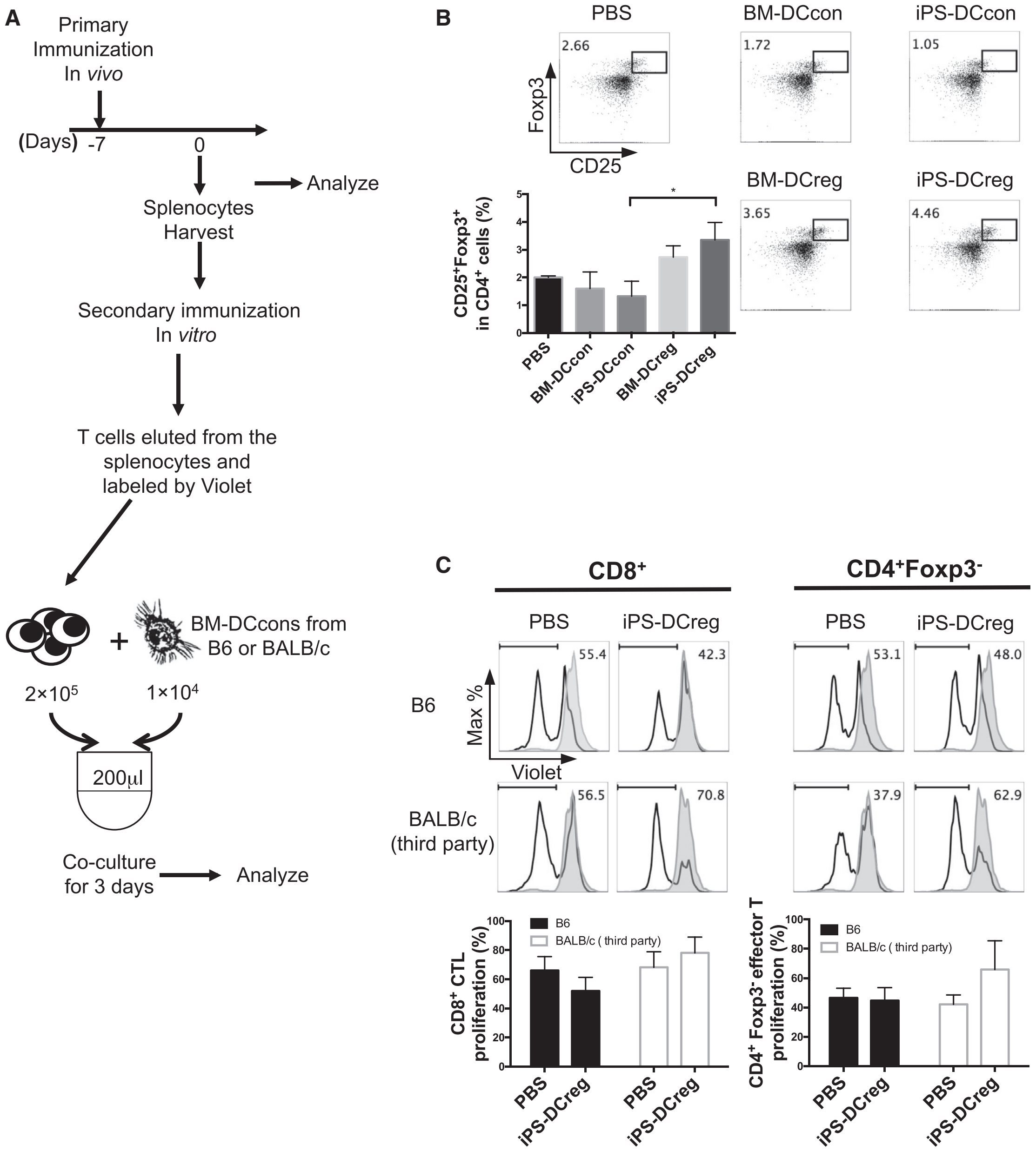

Figure 2. Administration of iPS-DCregs Generates Tregs In Vivo

(A) CBA mice were immunized with four types of B6 DCs (BM-DCcons, iPS-DCcons, BM-DCregs, iPS-DCregs) on day −7 and were euthanized on day 0. SPCs were harvested for FCM and one-way MLR (secondary immunization).

(B) The percentage of $CD25^+$ $FOXP3^+$ cells in the spleen from iPS-DCregs-immunized CBA was significantly increased compared with other groups (n = 4 in the PBS control group, n = 3 each in other groups, mean ± SD, pooled from three independent experiments). Statistical analysis was determined by one-way ANOVA and Tukey's test. *$p < 0.05$.

(C) T cells from B6 iPS-DCregs immunized with CBA were co-cultured with B6 BM-DCcons for 3 days. Proliferation of Teffs was determined by Violet dilution gated on $CD8^+$ or $CD4^+$ $FOXP3^-$ population (n = 3 in each group, mean ± SD, pooled from three independent experiments). Statistical analysis was determined by one-way ANOVA and Tukey's test. No statistically significant differences were observed between these groups.

allograft rejection. We intravenously injected 1×10^6 B6 iPS-DCregs or other B6-derived DCs (BM-DCcons, iPS-DCcons, and BM-DCregs) into CBA recipients 7 days prior to receiving a B6 heart allograft in the absence of IS drug therapy. The MST of pretreatment with BM-DCcons (n = 8, MST 8 days) and iPS-DCcons (n = 9, MST 8 days)

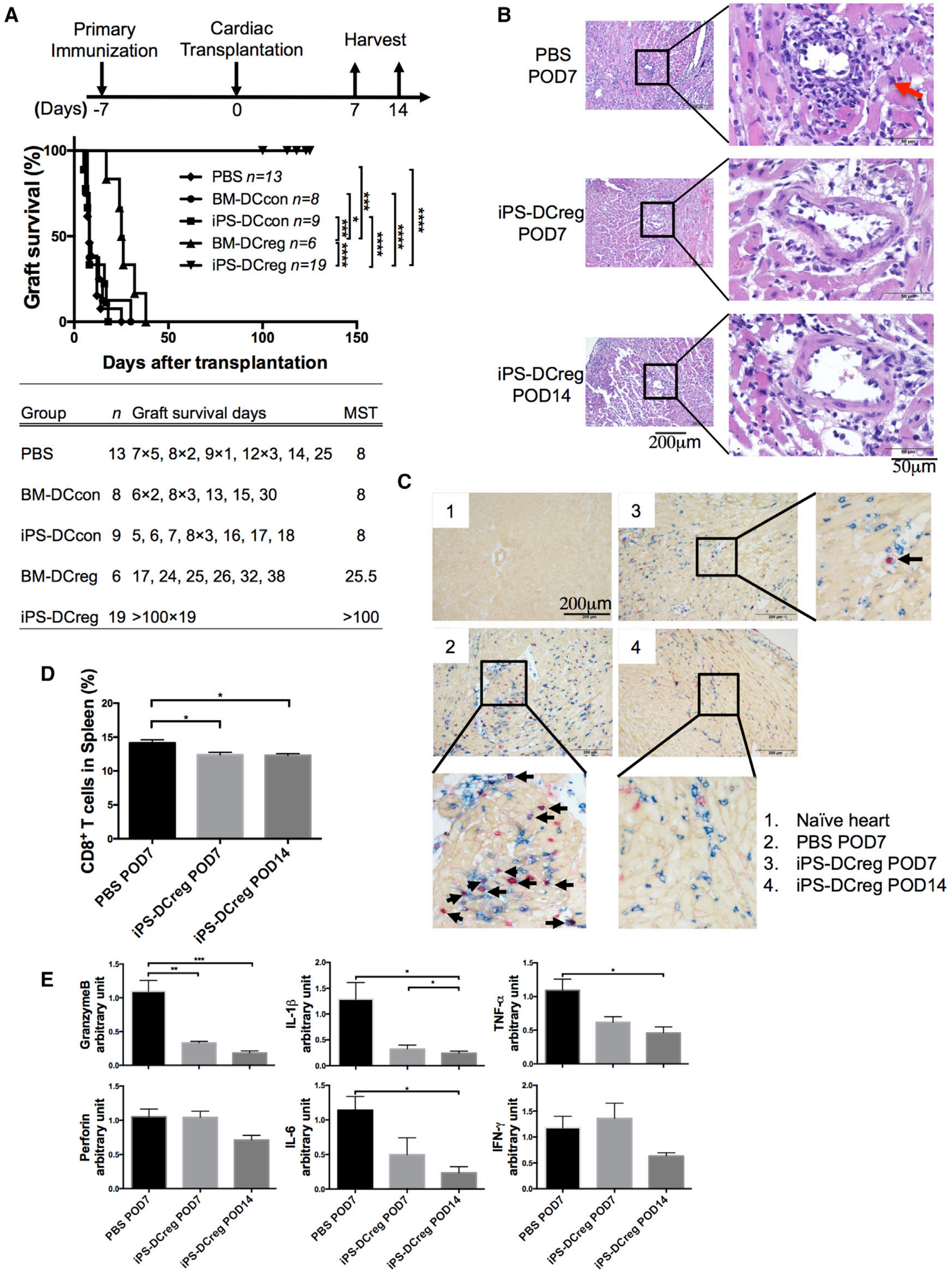

Group	n	Graft survival days	MST
PBS	13	7×5, 8×2, 9×1, 12×3, 14, 25	8
BM-DCcon	8	6×2, 8×3, 13, 15, 30	8
iPS-DCcon	9	5, 6, 7, 8×3, 16, 17, 18	8
BM-DCreg	6	17, 24, 25, 26, 32, 38	25.5
iPS-DCreg	19	>100×19	>100

Figure 3. iPS-DCreg Immunization Induces Permanent Acceptance of Allogeneic Cardiac Grafts and Decreases CD8+ T Cells in Grafts and Spleen

(A) 1×10^6 B6 DCs were injected intravenously into CBA mice 7 days before heterotopic cardiac transplantation. A statistical evaluation of graft survival was performed using Kaplan-Meier curves and compared using log-rank tests. *p < 0.05, ***p < 0.001, ****p < 0.0001.

(legend continued on next page)

was almost the same as that of the PBS control group. Conversely, administration of BM-DCregs significantly prolonged the survival of cardiac allografts as previously described. Notably, immunization of iPS-DCregs resulted in permanent acceptance of the allografts (n = 19, MST >100 days) (Figure 3A). We noticed that administration of the same number of BM-DCregs and iPS-DCregs induced the protective effects of different allografts. We thought that this might be caused by the difference in purity ($CD11b^+CD11c^+$%) between BM-DCregs (mean ± SD: 54.2% ± 3.9%) and iPS-DCregs (81.4% ± 2.2%) (Figure S1B). Next, we reduced the dose of iPS-DCregs to address this hypothesis. The MST of the half-dose (5×10^5) group was 26 days (n = 7), which prolonged allograft survival but did not lead to permanent acceptance (Figure S3B).

Histology showed serious lymphocyte infiltration around the coronary arteries in PBS-treated grafts on postoperative day 7 (POD7). In contrast, lymphocyte infiltration in the iPS-DCregs-immunized grafts was markedly reduced (Figure 3B).

iPS-DCregs Reduced CTLs and Downregulated Proinflammatory Cytokine

The infiltration of $CD8^+$bromodeoxyuridine$^+$ ($BrdU^+$) T cells in allografts was significantly decreased in the iPS-DCregs pretreatment group compared with the PBS group on POD7, and was further reduced on POD14 (Figure 3C). We checked the splenocytes from recipients and found that $CD8^+$ T cells (%) were also significantly reduced in the iPS-DCregs pretreatment group, which was consistent with the allograft assessment (Figure 3D).

As cytotoxic T lymphocyte (CTL)-induced cell death is initiated by the Perforin/Granzyme B pathway (Hayashida et al., 2000), we detected the mRNA expression of Perforin/Granzyme B in allografts. Logically, the levels of both Perforin and Granzyme B were lower in the grafts from iPS-DCregs-treated recipients compared with the grafts from the non-treatment group. Also, other proinflammatory cytokines, such as tumor necrosis factor α (TNF-α), interleukin-1β (IL-1β), IL-6, inducible nitric oxide synthase, and HO-1, were downregulated in the grafts from iPS-DCregs-treated recipients compared with those from non-treatment recipients, except IFN-γ (Figure 3E).

Tregs Play a Key Role in Acceptance of iPS-DCregs-Induced Allografts

Tregs markedly increased in allografts from the iPS-DCregs-immunized group in both immunohistochemistry and FCM assessments (Figures 4A and 4B [upper]). We continuously detected the activity markers of Tregs, CTLA-4, and GITR. CTLA-4 is a critical regulator of T cell responses as a co-stimulation (CD28/CD80-86) blocker and through other pathways (Hou et al., 2015; Krummey and Ford, 2014; Soskic et al., 2014). GITR (glucocorticoid-induced TNF receptor-related protein) appears to be a marker of activated Tregs and is widely used in functional studies on Tregs (Hilchey et al., 2007; Ronchetti et al., 2015). We found that in addition to the increased number of Tregs, the expression of CTLA-4 and GITR was significantly increased in the allografts from the iPS-DCregs-immunized group (Figure 4B, middle and lower). The spleen acts like a control tower in this process. The increase of activated Tregs in the spleen is totally consistent with the allograft (Figure S4).

We demonstrated that Tregs played a key role in allograft acceptance induced by iPS-DCregs, and the protective effects were donor specific in the following three tests. First, recipients were treated with iPS-DCregs at 1×10^6 (full dose) 7 days prior to transplantation, then anti-CD25 monoclonal antibody (mAb) (clone: PC61) (1 mg/mouse) was injected into the peritoneum to deplete Tregs (Couper et al., 2009; Hirai et al., 2016; Liu et al., 2012; Miller et al., 2015; Setiady et al., 2010). Tregs depletion broke the permanent acceptance of allografts induced by iPS-DCregs (n = 6, MST 44 days) (Figures 5A and 5D). Second, adoptive transfer (AT) of splenocytes from long-term surviving recipients (>POD100) into naive CBA (secondary recipients) led to B6-derived allograft acceptance (n = 4, MST >100 days) while third-party (BALB/c)-derived allografts were rejected (n = 3, MST 10 days). This indicated that the protective effect was donor specific (Figures 5B and 5D). Lastly, we injected anti-CD25 mAb into long-term surviving recipients. Three days later, we isolated their SPCs and adoptively transferred them to naive CBA (secondary recipients). This treatment reversed the AT-induced allograft acceptance (n = 4, MST 8.5 days) (Figures 5C and 5D), which indicated that donor-specific

(B) Cardiac allografts of POD7 and POD14 were stained with H&E. The red arrow indicates the increased lymphocytic infiltration around the coronary artery in PBS treated allograft.
(C) Allografts were stained with anti-CD8 (blue), collagen IV (yellowish-brown), and BrdU (red) by triple immunostaining. The black arrows indicate the CD8 and BrdU double positive lymphocytes in the grafts.
(D) Spleen cells (SPCs) were harvested on POD7 and POD14 for $CD8^+$ T cell assessment by FCM analysis (n = 4 in each group, mean ± SD, pooled from four independent experiments). *p < 0.05.
(E) The mRNA expression of GranzymeB and Perforin, IFN-γ, TNF-α, IL-1β, and IL-6 in allografts was measured by qRT-PCR (n = 9 in the PBS-POD7 group, n = 6 in the iPS-DCregs POD7 group, n = 5 in the iPS-DCregs POD14 group; mean ± SD, pooled from five independent experiments). Statistical analysis was determined by one-way ANOVA and Tukey's test. *p < 0.05, **p < 0.01, ***p < 0.001.

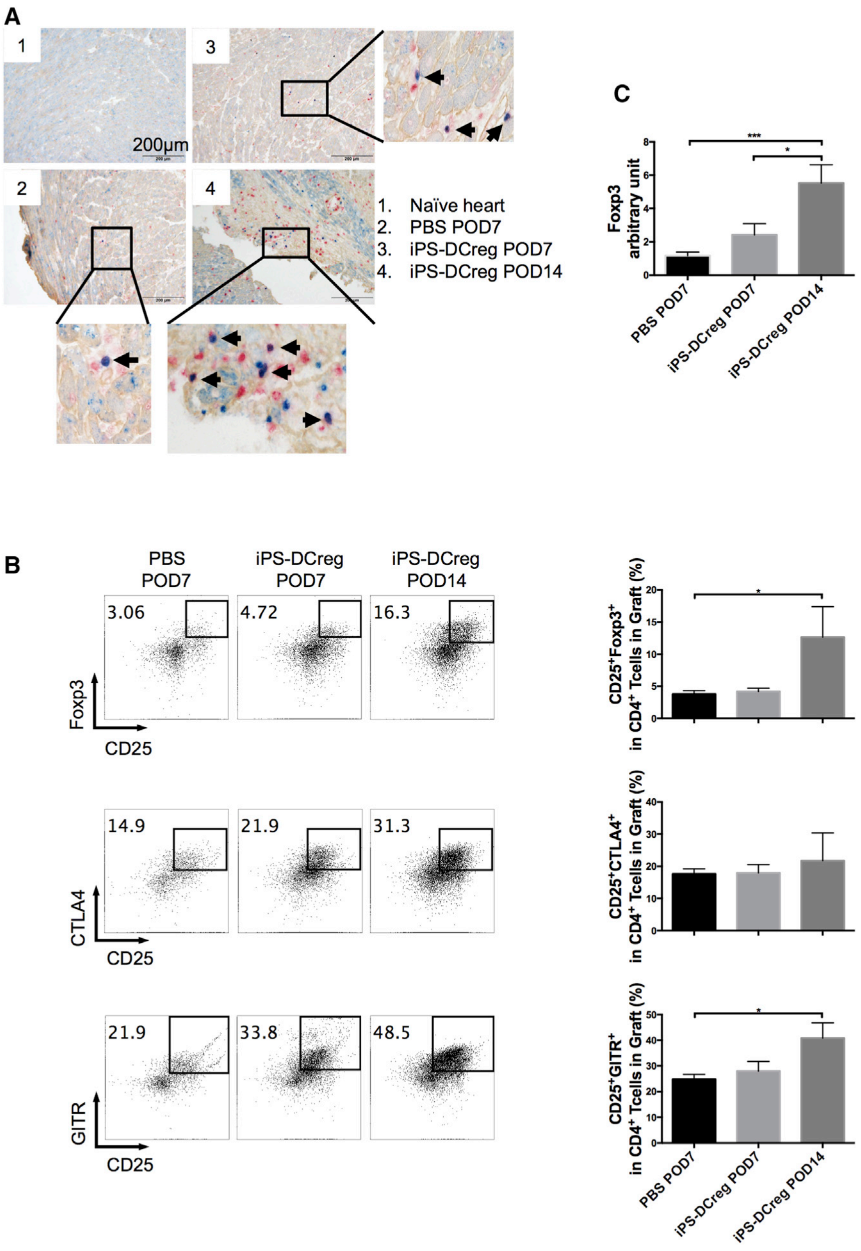

Figure 4. iPS-DCreg Immunization Increases Activated Tregs in Allografts

(A) Allografts were harvested on POD7 and POD14, and were stained with anti-FOXP3 (blue), collagen IV (yellowish-brown), and BrdU (red). The $FOXP3^{+}BrdU^{+}$ cells are shown in purple (indicated by the black arrows). Naive B6 hearts served as the control.

(B) The infiltrating lymphocytes in the grafts were separated and triple stained for CD4/CD25/FOXP3 or CD4/CD25/CTLA-4 or CD4/CD25/GITR (n = 5 in PBS-POD7 group, n = 3 in iPS-DCregs POD7 and iPS-DCregs POD14 group; mean ± SD, pooled from three independent experiments). Statistical analysis was determined by one-way ANOVA and Tukey's test. $^{*}p < 0.05$.

(C) The mRNA expression of FOXP3 in allografts harvested on POD7 and POD14 was detected by qRT-PCR (n = 9 in PBS-POD7 group, n = 6 in iPS-DCregs POD7 group, n = 5 in iPS-DCregs POD14 group; mean ± SD, pooled from five independent experiments). Statistical analysis was determined by one-way ANOVA and Tukey's test. $^{*}p < 0.05$, $^{***}p < 0.001$.

Tregs played an essential role in the maintenance phase of donor-specific tolerance.

TGF-β1 Blockage Interrupts the Protective Effect Induced by iPS-DCregs

TGF-β1 is one of the primary cytokines in immunosuppression. We therefore examined the role of TGF-β1 in allograft acceptance induced by iPS-DCregs.

First, as shown previously, the mRNA expression of TGF-β1 of iPS-DCregs was significantly higher compared with BM-DCcons (Figure 6A, right; Zhang et al., 2014). Second, the TGF-β1 molecule expression in the $CD11b^{+}$ $CD11c^{+}$ population of iPS-DCregs was significantly higher compared with BM-DCcons (Figure 6A, left). Third, membrane-bound TGF-β1 in Tregs was significantly increased in the SPCs isolated from the iPS-DCregs-treated group (Figure 6B). This evidence reminded us that TGF-β1 must play a critical function in the protective effect induced by iPS-DCregs. We then demonstrated this through the following tests with anti-TGF-β1 mAb.

First, recipients were treated with iPS-DCregs at 1×10^{6} on day −7 and anti-TGF-β1 mAb (400 µg/mouse intraperitoneally) on days −7, −5, −3, and 1 to block TGF-β1 before transplantation. This treatment prevented the permanent

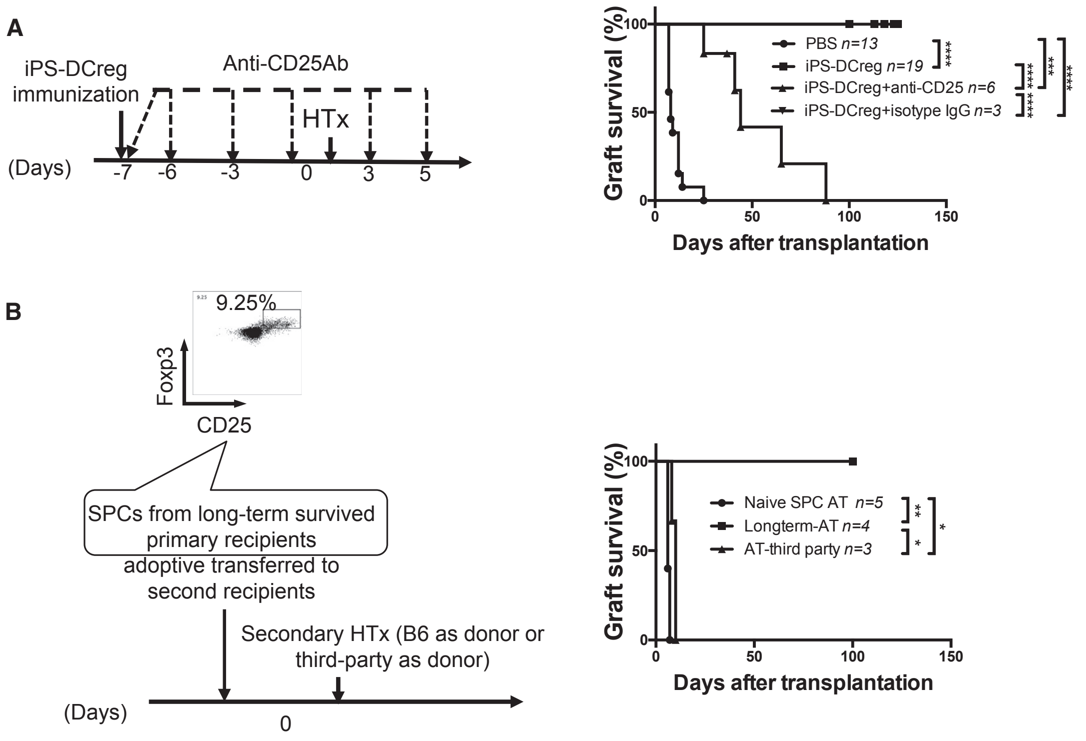

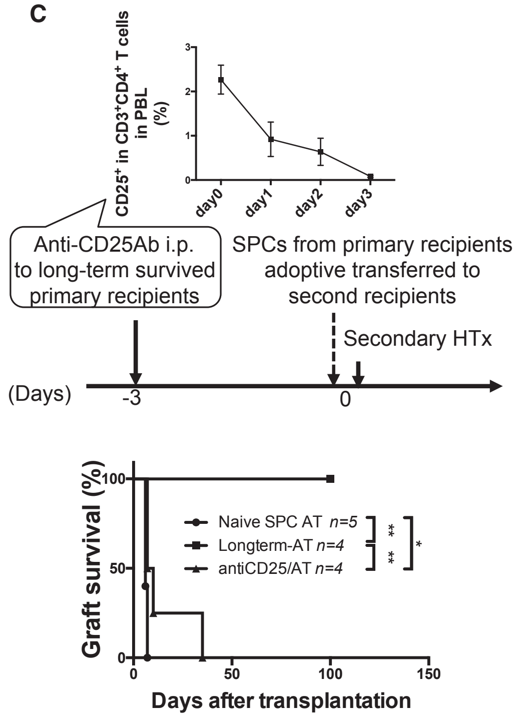

D

Group	n	Graft survival days	MST
PBS	13	7×5, 8×2, 9×1, 12×3, 14, 25	8
iPS-DCreg	19	>100×19	>100
iPS-DCreg+anti-CD25Ab	6	25, 37, 41, 44, 65, 88	44
iPS-DCreg+isotype IgG	3	>100×3	>100
Naïve SPC AT	5	6×3, 7×2	6
Long-term-AT	4	>100×4	>100
AT- BALB/c third party	3	8, 10, 10	10
anti-CD25Ab/AT	4	7×2, 10, 35	8.5

Figure 5. Tregs Generated by iPS-DCreg Immunization Are Donor Specific and Play a Key Role in Tolerance Induction and Maintenance

(A) CBA recipient mice were treated with B6 iPS-DCregs and anti-CD25 mAb. A statistical evaluation of graft survival was performed using Kaplan-Meier curves and compared using log-rank tests. $^{***}p < 0.001$, $^{****}p < 0.0001$.

(B) SPCs harvested from iPS-DCregs-treated recipients (primary recipients) on POD100, which included 9.25% Tregs in the $CD4^{+}$ population, were adoptively transferred to naive CBA (secondary recipients) at 5×10^{7}, and B6 hearts or BALB/c hearts (third party) were

(legend continued on next page)

acceptance of allografts (n = 8, MST 28.5 days) (Figures 6C and 6E).

Second, recipients were treated with iPS-DCregs at 1×10^6 on day −7 and then with anti-TGF-β1 mAb (400 μg/mouse intraperitoneally) on days 0, 1, 3, 5, and 7 to block TGF-β1 after transplantation. However, the blockage of TGF-β1 after transplantation did not impede the allograft acceptance induced by iPS-DCregs (n = 4, MST >100 days) (Figures 6D and 6E).

Finally, CBA mice were treated with B6 iPS-DCregs at 1×10^6 on day −7 and anti-TGF-β1 mAb (400 μg/mouse, intraperitoneally) on days −7, −5, −3, and 1 to block TGF-β1. SPCs were harvested on day 0 for FCM (Figure 7A). iPS-DCregs immunization enhanced TGF-β1 expression in SPCs, while anti-TGF-β1 mAb clearly blocked TGF-β1 (Figure 7B). We then scored how the TGF-β1 blockade changed the proportion and character of Tregs induced by iPS-DCregs. Firstly, we demonstrated that the number of $CD4^+CD25^+$ cells in SPCs was increased by iPS-DCregs immunization while anti-TGF-β1 blocked this effect (Figure 7C, upper). Furthermore, we detected the expression of FOXP3, Ki-67, CCR4, and CCR7 in $CD4^+CD25^+$ cells, which were widely used to gauge the activity and transmigration ability of Tregs (Sugiyama et al., 2013; Zhang et al., 2009a). The percentage of $FOXP3^+CCR4^+Ki\text{-}67^{hi}$ and $FOXP3^+CCR7^+Ki\text{-}67^{hi}$ in $CD4^+CD25^+$ cells was increased by iPS-DCregs immunization but was decreased by TGF-β1 blockage (Figure 7C-middle and lower). We then calculated the percentage of $CD4^+CD25^+$ $FOXP3^+CCR4^+$ $Ki\text{-}67^{hi}$ and $CD4^+CD25^+FOXP3^+CCR7^+Ki\text{-}67^{hi}$ in the total spleen. Also, iPS-DCregs immunization increased the percentage of these two populations in SPCs (Figure 7D). These results indicated that iPS-DCregs immunization not only increased the number of Tregs but also enhanced the activity and transmigration capability of Tregs, which worked in a TGF-β1-dependent manner.

DISCUSSION

Donor-specific tolerance that does not compromise the overall immune response is the ultimate goal in the transplantation field. DCregs-based therapies could potentially promote donor-specific tolerance to prevent allograft rejection and graft-versus-host disease (Bonham et al., 2002; DePaz et al., 2003; Garrovillo et al., 2001; Lan et al., 2006; Lutz et al., 2000; Min et al., 2000; Mirenda et al., 2004; Morelli and Thomson, 2007; Sato et al., 2003b; Turnquist et al., 2007; Zhang et al., 2008). Both donor-type DCregs (Bonham et al., 2002; DePaz et al., 2003; Fu et al., 1996; Lan et al., 2006; Lu et al., 1997; Lutz et al., 2000; Min et al., 2000; Taner et al., 2005; Zhang et al., 2008) and recipient-type DCregs (loaded with donor antigen) (Ali et al., 2000; Beriou et al., 2005; Garrovillo et al., 1999; Oluwole et al., 2001; Peche et al., 2005; Sato et al., 2003b) were reported to be able to prolong allograft survival through different pathways (direct, indirect, semi-direct). However, most of these studies are based on murine/rat bone marrow stem cells and human blood mononuclear cell-derived DCs, which require a large number of progenitor cells. Also, the quantity and quality of cultured DCs were inconsistent because of different ages, health conditions, and other variables among the sample. Recently, Kudo et al. (2014) differentiated donor-type macrophage-like IS cells from mouse ESCs, and these cells were found to prolong allograft survival. However, the ethical issues and patient-matched limitations of ESCs prevented them from being used in the clinical setting. In our previous study, we successfully differentiated DCregs from iPSCs (Zhang et al., 2014). The present study addressed the hypothesis of whether the administration of donor-type iPS-DCregs was capable of generating donor-specific tolerance.

Several conclusions can be drawn from the current study. First, iPS-DCregs not only indicate a high purity of $CD11b^+CD11c^+$ cells, but also retain a stable "immature" phenotype, even in the presence of strong maturational stimulus, IFN-γ. Many groups treated recipients with at least 2×10^6 bone marrow-derived DCregs, immature DCs, or other suppressive cells (such as myeloid-deprived suppressor cells) to achieve prolonged allograft survival (Arakawa et al., 2014; Fu et al., 1996; Rastellini et al., 1995; Tiao et al., 2005). In this study, 1×10^6 iPS-DCregs induced permanent allograft acceptance while BM-DCregs did not. The co-stimulator (CD40, CD80, CD86) and MHC-II molecule expressions in the $CD11b^+CD11c^+$ population were not significantly different between BM-DCregs and iPS-DCregs. However, iPS-DCregs have a significantly higher percentage of $CD11c^+$ in the $CD11b^+$ population compared with BM-DCregs. We thought that the high $CD11b^+CD11c^+$ purity of iPS-DCregs was the reason why

transplanted into the second recipients. A statistical evaluation of graft survival was performed using Kaplan-Meier curves and compared using log-rank tests. *$p < 0.05$, **$p < 0.01$.

(C) Anti-CD25 mAb was injected into the primary recipients on POD97. The depletion of $CD25^+$ cells was monitored in peripheral blood (PBL) by FCM. SPCs were then isolated from the primary recipients on POD100 and adoptively transferred to naive CBA (secondary recipients) at 5×10^7 just before the second transplantation. A statistical evaluation of graft survival was performed using Kaplan-Meier curves and compared using log-rank tests. *$p < 0.05$, **$p < 0.01$.

(D) Graft survival data in this figure are presented in detail.

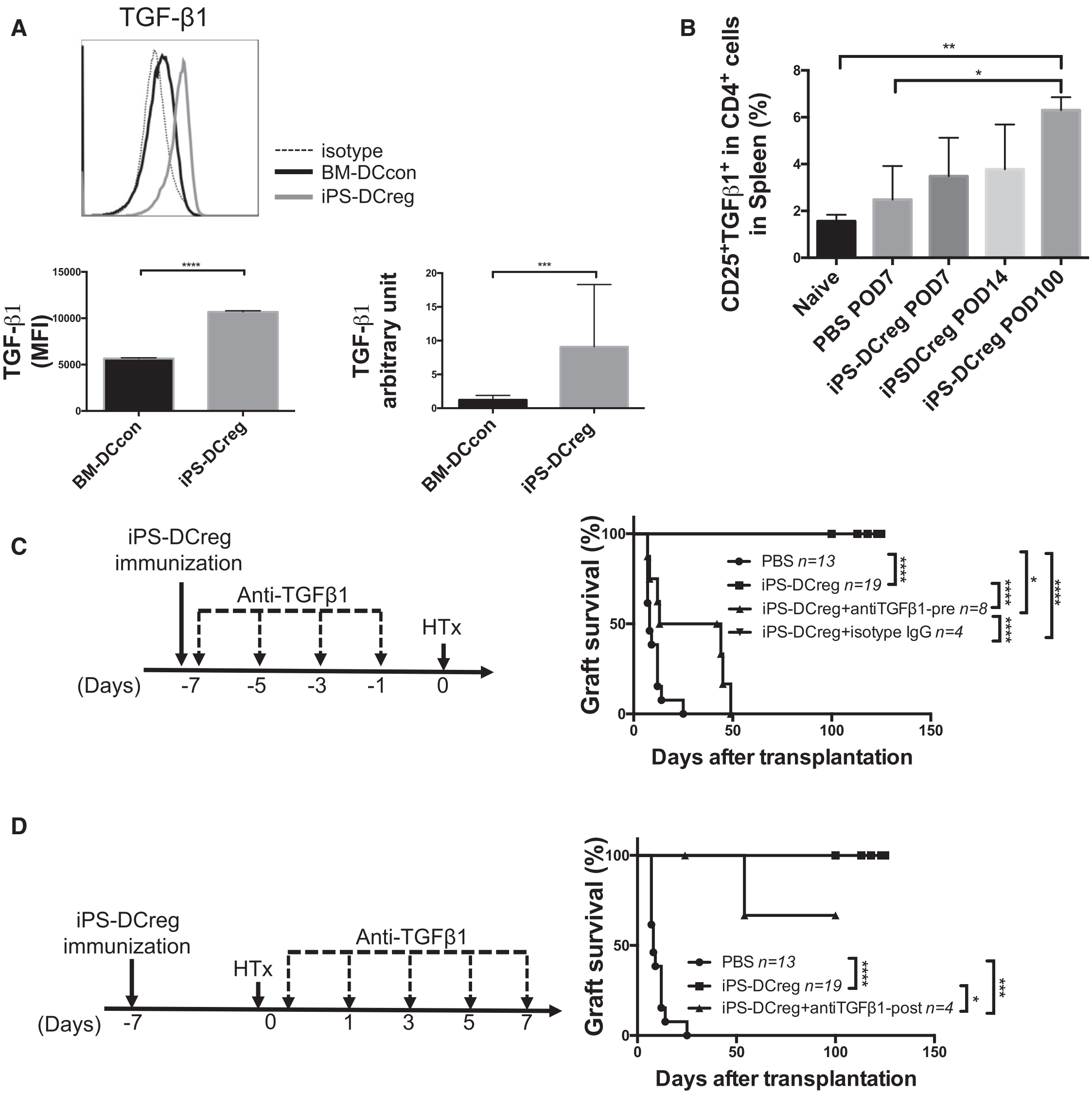

E

Group	*n*	Graft survival days	MST
No treat	13	7×5, 8×2, 9×1, 12×3, 14, 25	8
iPS-DCreg	19	>100×19	>100
iPS-DCreg+anti-TGFβ1-pre	8	7, 8, 12, 13, 42, 44, 45, 49	28.5
iPS-DCreg+anti-TGFβ1-post	4	>24, >54, >100×2	>100
iPS-DCreg+isotype IgG	4	>100×4	>100

Figure 6. TGF-β1 Is More Essential in the Primary Vaccination than in the Secondary Immunization

(A) The bar graph shows the TGF-β1 expression in the $CD11b^+CD11c^+$ population and the mean fluorescence intensity (MFI) is calculated (n = 3 in each group, mean ± SD, pooled from three independent experiments). The mRNA expression of TGF-β1 was measured (n = 5 in each group, mean ± SD, pooled from five independent experiments). Statistical analysis was determined by Student's t test. $***p < 0.001$, $****p < 0.0001$.

(legend continued on next page)

the same dose of BM-DCregs and iPS-DCregs led to a different outcome. The number of administered $CD11b^{+}$ $CD11c^{+}$ cells is known to directly affect the tolerance-inducing reaction (Morelli and Thomson, 2007). Recipients treated with a half-dose of iPS-DCregs indicated a graft survival similar to that with a full-dose of BM-DCregs, which was identified in our hypothesis. Our findings suggest that the addition of iPS-DCregs into MLR culture and PLN significantly suppressed the T cell proliferative response. These characteristics engineered iPS-DCregs as important “regulatory cellular vaccines” in the allogeneic transplantation model (Morelli and Thomson, 2007; Sato et al., 2003a, 2003b; Thomson et al., 2009).

Second, donor-type iPS-DCregs activated donor-specific Tregs. Donor-type DCregs expressing donor MHC molecules traveled to the recipient’s secondary lymphoid tissues and interacted with T cells through the “direct pathway” of allorecognition. The direct pathway of allorecognition is considered to be the most powerful mechanism to instigate early acute graft rejection (Morelli and Thomson, 2007). Figuratively speaking, pretreatment of donor-type iPS-DCregs acted as an immune suppressive vaccine, which led to a primary immune response. According to the “two-signal” hypothesis of T cell activation (Mueller et al., 1989), recipient naive T cells interacted with allogeneic MHC molecules on DCregs through a direct pathway, followed by the delivery of potent signal 1 plus poor signal 2 by DCregs to naive T cells. This resulted in the generation of donor-specific Tregs (Bakdash et al., 2013) and anergy of donor-specific Teffs (Sato et al., 2003a). On the other hand, Tiao et al. (2005) pretreated recipients with recipient-type immature BM-DCs pulsing with donor antigens, which prolonged the allograft MST by 40 days. However, in our study, recipient-type BM-DCregs with or without pulsing with donor antigens could not protect allografts from acute rejection (MST 7 days). In Tiao et al.’s (2005) study, 2×10^{6} immature BM-DCs were intravenously injected into recipients, while we only used 1×10^{6} BM-DCregs. The different DC dose may be the reason why recipient-type DCregs treatment did not work in our preliminary study.

Third, Tregs generated by iPS-DCregs were vital in allograft tolerance (Joffre et al., 2008; Kitazawa et al., 2007; Sakaguchi, 2004; Zhang et al., 2009b), especially in the maintenance phase (1–3 months post operation). Interestingly, donor-type iPS-DCregs served as the “primary vaccination,” which “prepared” the inhibited immune situation for the allografts. Based on this analogy, the alloantigen loaded by allografts served as the “secondary vaccination.” We hypothesized that after transplantation, the alloantigen interacted with the Tregs and precursor Tregs induced by the primary vaccination and then further activated alloantigen-specific Tregs. The number of Tregs and the expression of CTLA-4 in Tregs were significantly higher on POD100 compared with POD7 (Figure S4), which indicated that donor-specific Tregs expanded and activated unceasingly (Schubert et al., 2014; Wing et al., 2008). This may have caused the alloantigen loaded on the allograft to work as a stimulator to promote the clonal expansion of donor-specific Tregs, which we referred to as the secondary vaccination.

Fourth, IFN-γ, a key inflammatory cytokine produced by Teffs, was higher in the iPS-DCregs-treated group than in the non-treated group on POD7, but became lower on POD14. Some groups reported that IFN-γ knockout (KO) and IFN-γ receptor (IFN-γR) KO recipients rejected allografts much more quickly compared with wild-type, because IFN-γ plays a crucial role in Teff apoptosis through several pathways (Morita et al., 2015; Ring et al., 1999). Thus, we speculate that IFN-γ is essential in DCregs-induced tolerance via Teff apoptosis.

Fifth, TGF-β1 was certainly required in the “primary vaccination” but was not essential for the early stage of “secondary vaccination.” We have revealed here that the blockage of TGF-β1 during the period between iPS-DCregs treatment and allotransplantation prevented iPS-DCregs-induced allotolerance. However, the blockage of TGF-β1 post transplantation could not break the tolerance. Early studies demonstrated that TGF-β1 could generate Tregs through several pathways, for example, the inhibition of IL-2, the upregulation of cyclin-dependent kinase (CDK) inhibitors (p15, p21, and p27), and the downregulation of cell-cycle-promoting factors (c-myc, cyclin D2, CDK2, and cyclin E) (Wan and Flavell, 2007). We identified that the blockage of TGF-β1 not only decreased the number of Tregs induced by iPS-DCregs ($CD4^{+}CD25^{+}$%), but also downregulated the activity and transmigration ability of Tregs ($CD4^{+}CD25^{+}FOXP3^{+}CCR4^{+}Ki\text{-}67^{hi}$% and $CD4^{+}CD25^{+}FOXP3^{+}CCR7^{+}Ki\text{-}67^{hi}$%). The protective effects of Tregs on

(B) SPCs were harvested on POD7, POD14, and POD100, and triple stained for CD4/CD25/TGF-β1 for FCM. The quantitation is the percentage of $CD25^{+}TGF\text{-}\beta1^{+}$ cells in the $CD4^{+}$ cell population (n = 4 in POD100 group, n = 3 each in other groups; mean ± SD, pooled from three independent experiments). Statistical analysis was determined by one-way ANOVA and Tukey’s test. *$p < 0.05$, **$p < 0.01$.
(C) Anti-TGF-β1mAb was injected into the iPS-DCregs immunized CBA (recipient) before heart transplantation. A statistical evaluation of graft survival was performed using Kaplan-Meier curves and compared using log rank tests. *$p < 0.05$, ****$p < 0.0001$.
(D) Anti-TGF-β1 mAb was injected into the iPS-DCregs immunized CBA (recipient) after heart transplantation. A statistical evaluation of graft survival was performed using Kaplan-Meier curves and compared using log rank tests. *$p < 0.05$, ***$p < 0.001$, ****$p < 0.0001$.
(E) Graft survival data in this figure is presented in detail.

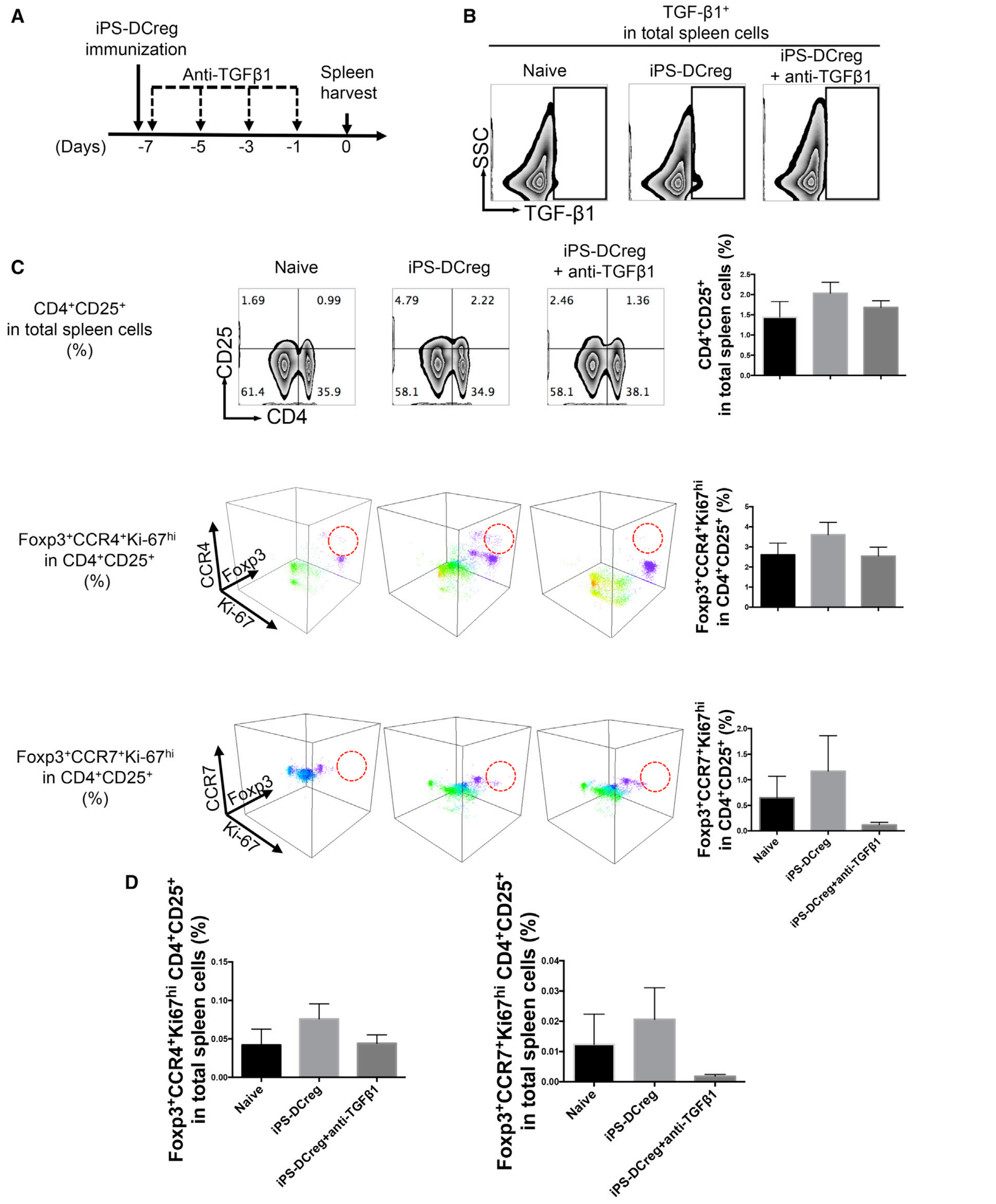

Figure 7. FOXP3^{+}CCR4^{+}Ki-67^{hi} and FOXP3^{+}CCR7^{+}Ki-67^{hi} Tregs Are Increased by iPS-DCreg Immunization in a TGF-β1-Dependent Pattern

(A) Anti-TGF-β1 mAb was injected into the iPS-DCregs-treated CBA (the same protocol as shown in Figure 6C), and SPCs were harvested.
(B) TGF-β1 blockade was identified by FCM.

(legend continued on next page)

allograft survival were abrogated if they failed to migrate to the graft due to CCR4 and CCR7 deficiency. Logistically, the protection was enhanced when Tregs were delivered locally into the grafts (Sugiyama et al., 2013; Zhang et al., 2009a). We believe that this is the reason why the blockage of TGF-β1 during the period between iPS-DCregs immunization and allotransplantation could prevent iPS-DCregs-induced allotolerance. Although our data showed that donor-type iPS-DCregs treatment induced donor-specific Tregs by upregulating TGF-β1, it remains possible that these Tregs and TGF-β1 may participate in non-specific immune suppression. Nonetheless, although our examination indicated that Tregs and TGF-β1 are two key factors in allotolerance induced by iPS-DCregs, there could be other mechanisms involved in this therapy. In addition, further research is needed before this therapy can be adapted for clinical application, including dose, timing, dosage, and/or combination with low-dose IS drugs.

In summary, we have successfully generated alloantigen-specific Tregs with therapeutic activity toward allorejection by infusion of donor-type iPS-DCregs. Although there are still many barriers to be overcome, we believe that iPS-DCregs offer a potentially efficient and reliable approach for use in transplantation and/or autoimmune diseases.

EXPERIMENTAL PROCEDURES

Study Design

Our primary research objective was to establish a method to generate donor antigen-specific Tregs in vivo in recipient mice by donor-type iPS-DCregs immunization and to search its core mechanism. The overall study design was a series of controlled laboratory experiments as indicated in the sections below.

In vivo experimental groups included 3–10 mice per group, with two exceptions. First, because an AT study requires many recipients' spleens on POD100 (endpoint), the graft survival data of the iPS-DCregs-treated group included 19 mice. Second, because most assessments needed to use the PBS group as a negative control, the grafts survival data of the PBS group included 13 mice. Mice were randomly assigned to each group, but the researchers were not blinded to the group identity.

Animals

Male CBA/N (CBA; $H\text{-}2k^k$), C57BL/6 (B6; $H\text{-}2k^b$), and BALB/c ($H\text{-}2k^d$) mice were purchased from the Shizuoka Laboratory Animal Center. C57BL/6-Tg (TCR-OT-I) Cbn (OT-I, $H\text{-}2k^b$) and C57BL/6-Tg (TCR-OT-II) Cbn (OT-II, $H\text{-}2k^b$) transgenic mice were kindly supplied by Dr. N. Ishii (Graduate School of Medicine, Tohoku University) and Dr. S. Nakae (The Institute of Medical Science, The University of Tokyo), respectively. All mice were bred and maintained under standard conditions and fed rodent food and water according to the guidelines of the Animal Use and Care Committee of the National Research Institute for Child Health and Development, Tokyo, Japan. All animal experiments were approved by this committee and performed according to its recommendations.

Tregs and TGF-β1 Blockage

Recipients were treated with anti-CD25 mAb (cat. #BE0012, clone PC61.5.3, BioXCell) (Hirata et al., 2007; Couper et al., 2009) or anti-TGF-β1 mAb (cat. #BE0057, clone 1D11.16.8, BioXCell) (Kasagi et al., 2014) by intraperitoneal administration. The schematics of the protocol are shown in the associated results and figures.

Additional Methods

Information regarding DC culture, heterotopic cardiac transplantation, MLR, PLNA, graft infiltration lymphocyte isolation, flow cytometry, histopathology, immunohistochemistry, and qRT-PCR is provided in Supplemental Experimental Procedures.

Statistical Analyses

The data were analyzed using GraphPad Prism, version 6.0 (GraphPad Software). One-way ANOVA and Tukey's test were used to compare the means of more than two groups. Student's t test was used to compare the means of two groups. A statistical evaluation of graft survival was performed using Kaplan-Meier curves and compared using log-rank tests. All in vitro experimental data were representative of at least three independent experiments. p Values of less than 0.05 were considered statistically significant.

SUPPLEMENTAL INFORMATION

Supplemental Information includes Supplemental Experimental Procedures, five figures, and one table and can be found with this article online at http://dx.doi.org/10.1016/j.stemcr.2017.03.020.

AUTHOR CONTRIBUTIONS

S.C., X.-K.L., P.Z., J.-M.C., and J.Z. conceived and designed the experiments. S.C., J.H., Q.Z., and R.A. performed the experiments. S.C., J.H., Q.Z., M.F., N.I., S.T., L.L., P.Z., J.-M.C., and J.Z. analyzed the data. S.C., R.A., and L.L. contributed reagents/materials/analysis tools. S.C., M.F., and X.-K.L. wrote the paper.

(C) SPCs were multiply stained with CCR4/Ki-67/CD4/CD25/FOXP3 or CCR4/Ki-67/CD4/CD25/FOXP3 for FCM. The expression of CCR4/Ki-67/FOXP3 and CCR4/Ki-67/FOXP3 in the $CD4^+$ $FOXP3^+$ population is presented in a 3D visualization (n = 3 each in other groups, mean ± SD, pooled from three independent experiments). Statistical analysis was determined by one-way ANOVA and Tukey's test. No statistically significant difference was observed between these groups.
(D) The quantitation is the percentage of $FOXP3^+CCR4^+$(or $CCR7^+$) $Ki\text{-}67^{hi}CD4^+CD25^+$ cells in total SPCs (n = 3 each in other groups, mean ± SD, pooled from three independent experiments). Statistical analysis was determined by one-way ANOVA and Tukey's test. No statistically significant difference was observed between these groups.

ACKNOWLEDGMENTS

The authors gratefully acknowledge Dr. H. Kimura for his critical comments and useful suggestions. The authors also thank S. Iwasaki and C. Huang for their technical assistance. The authors are deeply grateful to Prof. Naoto Ishii, who kindly supplied the OT-I transgenic mice. We would like to thank Dr. Julian Tang of the Department of Education for Clinical Research, National Center for Child Health and Development, for proofreading and editing this article. This study was supported by research grants from the National Center for Child Health and Development (26-6, 26-27 and 27-21), Ministry of Education, Culture, Sports, Science and Technology of Japan (Grants-in-Aid 15F15756 and 15K10043), Kawano Masanori Memorial Public Interest Incorporated Foundation for Promotion of Pediatrics, National Natural Science Foundation of China (grant nos. 81202302, 81370230, 81570279, and 81671550), Natural Science Foundation of Guangdong Province, China (grant no. 2014A030311041), and Science and Technology Program of Guangzhou, China (grant no. 201508020107).

Received: November 14, 2016
Revised: March 17, 2017
Accepted: March 27, 2017
Published: April 20, 2017

REFERENCES

Ali, A., Garrovillo, M., Jin, M.X., Hardy, M.A., and Oluwole, S.F. (2000). Major histocompatibility complex class I peptide-pulsed host dendritic cells induce antigen-specific acquired thymic tolerance to islet cells. Transplantation *69*, 221–226.

Arakawa, Y., Qin, J., Chou, H.S., Bhatt, S., Wang, L., Stuehr, D., Ghosh, A., Fung, J.J., Lu, L., and Qian, S. (2014). Cotransplantation with myeloid-derived suppressor cells protects cell transplants: a crucial role of inducible nitric oxide synthase. Transplantation *97*, 740–747.

Bakdash, G., Sittig, S.P., van Dijk, T., Figdor, C.G., and de Vries, I.J. (2013). The nature of activatory and tolerogenic dendritic cell-derived signal II. Front. Immunol. *4*, 53.

Beriou, G., Peche, H., Guillonneau, C., Merieau, E., and Cuturi, M.C. (2005). Donor-specific allograft tolerance by administration of recipient-derived immature dendritic cells and suboptimal immunosuppression. Transplantation *79*, 969–972.

Bonham, C.A., Peng, L., Liang, X., Chen, Z., Wang, L., Ma, L., Hackstein, H., Robbins, P.D., Thomson, A.W., Fung, J.J., et al. (2002). Marked prolongation of cardiac allograft survival by dendritic cells genetically engineered with NF-kappa B oligodeoxyribonucleotide decoys and adenoviral vectors encoding CTLA4-Ig. J. Immunol. *169*, 3382–3391.

Bradley, J.A. (2014). Transplant tolerance by Treg therapy. Am. J. Transplant. *14*, 5–6.

Couper, K.N., Lanthier, P.A., Perona-Wright, G., Kummer, L.W., Chen, W., Smiley, S.T., Mohrs, M., and Johnson, L.L. (2009). Anti-CD25 antibody-mediated depletion of effector T cell populations enhances susceptibility of mice to acute but not chronic Toxoplasma gondii infection. J. Immunol. *182*, 3985–3994.

Dantal, J., Hourmant, M., Cantarovich, D., Giral, M., Blancho, G., Dreno, B., and Soulillou, J.P. (1998). Effect of long-term immunosuppression in kidney-graft recipients on cancer incidence: randomised comparison of two cyclosporin regimens. Lancet *351*, 623–628.

DePaz, H.A., Oluwole, O.O., Adeyeri, A.O., Witkowski, P., Jin, M.X., Hardy, M.A., and Oluwole, S.F. (2003). Immature rat myeloid dendritic cells generated in low-dose granulocyte macrophage-colony stimulating factor prolong donor-specific rat cardiac allograft survival. Transplantation *75*, 521–528.

Ezzelarab, M., and Thomson, A.W. (2011). Tolerogenic dendritic cells and their role in transplantation. Semin. Immunol. *23*, 252–263.

Fu, F., Li, Y., Qian, S., Lu, L., Chambers, F., Starzl, T.E., Fung, J.J., and Thomson, A.W. (1996). Costimulatory molecule-deficient dendritic cell progenitors (MHC class II+, CD80dim, CD86-) prolong cardiac allograft survival in nonimmunosuppressed recipients. Transplantation *62*, 659–665.

Garrovillo, M., Ali, A., and Oluwole, S.F. (1999). Indirect allorecognition in acquired thymic tolerance: induction of donor-specific tolerance to rat cardiac allografts by allopeptide-pulsed host dendritic cells. Transplantation *68*, 1827–1834.

Garrovillo, M., Ali, A., Depaz, H.A., Gopinathan, R., Oluwole, O.O., Hardy, M.A., and Oluwole, S.F. (2001). Induction of transplant tolerance with immunodominant allopeptide-pulsed host lymphoid and myeloid dendritic cells. Am. J. Transplant. *1*, 129–137.

Hackstein, H., and Thomson, A.W. (2004). Dendritic cells: emerging pharmacological targets of immunosuppressive drugs. Nat. Rev. Immunol. *4*, 24–34.

Hayashida, M., Kawano, H., Nakano, T., Shiraki, K., and Suzuki, A. (2000). Cell death induction by CTL: perforin/granzyme B system dominantly acts for cell death induction in human hepatocellular carcinoma cells. Proc. Soc. Exp. Biol. Med. *225*, 143–150.

Hilchey, S.P., De, A., Rimsza, L.M., Bankert, R.B., and Bernstein, S.H. (2007). Follicular lymphoma intratumoral CD4+CD25+GITR+ regulatory T cells potently suppress CD3/CD28-costimulated autologous and allogeneic CD8+CD25- and CD4+CD25- T cells. J. Immunol. *178*, 4051–4061.

Hirai, T., Ishii, R., Miyairi, S., Ikemiyagi, M., Omoto, K., Ishii, Y., and Tanabe, K. (2016). Clonal deletion established via invariant NKT cell activation and costimulatory blockade requires in vivo expansion of regulatory T cells. Am. J. Transplant. *16*, 426–439.

Hirata, S., Matsuyoshi, H., Fukuma, D., Kurisaki, A., Uemura, Y., Nishimura, Y., and Senju, S. (2007). Involvement of regulatory T cells in the experimental autoimmune encephalomyelitis-preventive effect of dendritic cells expressing myelin oligodendrocyte glycoprotein plus TRAIL. J. Immunol. *178*, 918–925.

Hou, T.Z., Qureshi, O.S., Wang, C.J., Baker, J., Young, S.P., Walker, L.S., and Sansom, D.M. (2015). A transendocytosis model of CTLA-4 function predicts its suppressive behavior on regulatory T cells. J. Immunol. *194*, 2148–2159.

Joffre, O., Santolaria, T., Calise, D., Al Saati, T., Hudrisier, D., Romagnoli, P., and van Meerwijk, J.P. (2008). Prevention of acute and chronic allograft rejection with CD4+CD25+Foxp3+ regulatory T lymphocytes. Nat. Med. *14*, 88–92.

Kasagi, S., Zhang, P., Che, L., Abbatiello, B., Maruyama, T., Nakatsukasa, H., Zanvit, P., Jin, W., Konkel, J.E., and Chen, W. (2014). In vivo-generated antigen-specific regulatory T cells treat autoimmunity without compromising antibacterial immune response. Sci. Transl. Med. *6*, 241ra278.

Kitazawa, Y., Fujino, M., Wang, Q., Kimura, H., Azuma, M., Kubo, M., Abe, R., and Li, X.K. (2007). Involvement of the programmed death-1/programmed death-1 ligand pathway in CD4+CD25+ regulatory T-cell activity to suppress alloimmune responses. Transplantation *83*, 774–782.

Krummey, S.M., and Ford, M.L. (2014). Braking bad: novel mechanisms of CTLA-4 inhibition of T cell responses. Am. J. Transplant. *14*, 2685–2690.

Kudo, H., Wada, H., Sasaki, H., Tsuji, H., Otsuka, R., Baghdadi, M., Kojo, S., Chikaraishi, T., and Seino, K. (2014). Induction of macrophage-like immunosuppressive cells from mouse ES cells that contribute to prolong allogeneic graft survival. PLoS One *9*, e111826.

Lan, Y.Y., Wang, Z., Raimondi, G., Wu, W., Colvin, B.L., de Creus, A., and Thomson, A.W. (2006). "Alternatively activated" dendritic cells preferentially secrete IL-10, expand Foxp3+CD4+ T cells, and induce long-term organ allograft survival in combination with CTLA4-Ig. J. Immunol. *177*, 5868–5877.

Liu, Z., Hou, J., Chen, J., Tsumura, H., Ito, M., Ito, Y., Hu, X., and Li, X.K. (2012). Deletion of CD98 heavy chain in T cells results in cardiac allograft acceptance by increasing regulatory T cells. Transplantation *93*, 1116–1124.

Lu, L., Li, W., Fu, F., Chambers, F.G., Qian, S., Fung, J.J., and Thomson, A.W. (1997). Blockade of the CD40-CD40 ligand pathway potentiates the capacity of donor-derived dendritic cell progenitors to induce long-term cardiac allograft survival. Transplantation *64*, 1808–1815.

Lutz, M.B., Suri, R.M., Niimi, M., Ogilvie, A.L., Kukutsch, N.A., Rossner, S., Schuler, G., and Austyn, J.M. (2000). Immature dendritic cells generated with low doses of GM-CSF in the absence of IL-4 are maturation resistant and prolong allograft survival in vivo. Eur. J. Immunol. *30*, 1813–1822.

McMurchy, A.N., Bushell, A., Levings, M.K., and Wood, K.J. (2011). Moving to tolerance: clinical application of T regulatory cells. Semin. Immunol. *23*, 304–313.

Miller, M.L., Daniels, M.D., Wang, T., Chen, J., Young, J., Xu, J., Wang, Y., Yin, D., Vu, V., Husain, A.N., et al. (2015). Spontaneous restoration of transplantation tolerance after acute rejection. Nat. Commun. *6*, 7566.

Min, W.P., Gorczynski, R., Huang, X.Y., Kushida, M., Kim, P., Obataki, M., Lei, J., Suri, R.M., and Cattral, M.S. (2000). Dendritic cells genetically engineered to express Fas ligand induce donor-specific hyporesponsiveness and prolong allograft survival. J. Immunol. *164*, 161–167.

Mirenda, V., Berton, I., Read, J., Cook, T., Smith, J., Dorling, A., and Lechler, R.I. (2004). Modified dendritic cells coexpressing self and allogeneic major histocompatibility complex molecules: an efficient way to induce indirect pathway regulation. J. Am. Soc. Nephrol. *15*, 987–997.

Moreau, A., Varey, E., Beriou, G., Hill, M., Bouchet-Delbos, L., Segovia, M., and Cuturi, M.C. (2012). Tolerogenic dendritic cells and negative vaccination in transplantation: from rodents to clinical trials. Front. Immunol. *3*, 218.

Morelli, A.E., and Thomson, A.W. (2003). Dendritic cells: regulators of alloimmunity and opportunities for tolerance induction. Immunol. Rev. *196*, 125–146.

Morelli, A.E., and Thomson, A.W. (2007). Tolerogenic dendritic cells and the quest for transplant tolerance. Nat. Rev. Immunol. *7*, 610–621.

Morita, M., Joyce, D., Miller, C., Fung, J.J., Lu, L., and Qian, S. (2015). Rejection triggers liver transplant tolerance: involvement of mesenchyme-mediated immune control mechanisms in mice. Hepatology *62*, 915–931.

Mueller, D.L., Jenkins, M.K., and Schwartz, R.H. (1989). Clonal expansion versus functional clonal inactivation: a costimulatory signalling pathway determines the outcome of T cell antigen receptor occupancy. Annu. Rev. Immunol. *7*, 445–480.

Oluwole, O.O., Depaz, H.A., Gopinathan, R., Ali, A., Garrovillo, M., Jin, M.X., Hardy, M.A., and Oluwole, S.F. (2001). Indirect allorecognition in acquired thymic tolerance: induction of donor-specific permanent acceptance of rat islets by adoptive transfer of allopeptide-pulsed host myeloid and thymic dendritic cells. Diabetes *50*, 1546–1552.

Peche, H., Trinite, B., Martinet, B., and Cuturi, M.C. (2005). Prolongation of heart allograft survival by immature dendritic cells generated from recipient type bone marrow progenitors. Am. J. Transplant. *5*, 255–267.

Raich-Regue, D., Glancy, M., and Thomson, A.W. (2014). Regulatory dendritic cell therapy: from rodents to clinical application. Immunol. Lett. *161*, 216–221.

Rastellini, C., Lu, L., Ricordi, C., Starzl, T.E., Rao, A.S., and Thomson, A.W. (1995). Granulocyte/macrophage colony-stimulating factor-stimulated hepatic dendritic cell progenitors prolong pancreatic islet allograft survival. Transplantation *60*, 1366–1370.

Ring, G.H., Saleem, S., Dai, Z., Hassan, A.T., Konieczny, B.T., Baddoura, F.K., and Lakkis, F.G. (1999). Interferon-gamma is necessary for initiating the acute rejection of major histocompatibility complex class II-disparate skin allografts. Transplantation *67*, 1362–1365.

Roncarolo, M.G., Levings, M.K., and Traversari, C. (2001). Differentiation of T regulatory cells by immature dendritic cells. J. Exp. Med. *193*, F5–F9.

Ronchetti, S., Ricci, E., Petrillo, M.G., Cari, L., Migliorati, G., Nocentini, G., and Riccardi, C. (2015). Glucocorticoid-induced tumour necrosis factor receptor-related protein: a key marker of functional regulatory T cells. J. Immunol. Res. *2015*, 171520.

Sakaguchi, S. (2004). Naturally arising CD4+ regulatory T cells for immunologic self-tolerance and negative control of immune responses. Annu. Rev. Immunol. *22*, 531–562.

Sato, K., Yamashita, N., Baba, M., and Matsuyama, T. (2003a). Modified myeloid dendritic cells act as regulatory dendritic cells to induce anergic and regulatory T cells. Blood *101*, 3581–3589.

Sato, K., Yamashita, N., Yamashita, N., Baba, M., and Matsuyama, T. (2003b). Regulatory dendritic cells protect mice from murine

acute graft-versus-host disease and leukemia relapse. Immunity *18*, 367–379.

Schubert, D., Bode, C., Kenefeck, R., Hou, T.Z., Wing, J.B., Kennedy, A., Bulashevska, A., Petersen, B.S., Schaffer, A.A., Gruning, B.A., et al. (2014). Autosomal dominant immune dysregulation syndrome in humans with CTLA4 mutations. Nat. Med. *20*, 1410–1416.

Setiady, Y.Y., Coccia, J.A., and Park, P.U. (2010). In vivo depletion of CD4+FOXP3+ Treg cells by the PC61 anti-CD25 monoclonal antibody is mediated by FcgammaRIII+ phagocytes. Eur. J. Immunol. *40*, 780–786.

Soskic, B., Qureshi, O.S., Hou, T., and Sansom, D.M. (2014). A transendocytosis perspective on the CD28/CTLA-4 pathway. Adv. Immunol. *124*, 95–136.

Sugiyama, D., Nishikawa, H., Maeda, Y., Nishioka, M., Tanemura, A., Katayama, I., Ezoe, S., Kanakura, Y., Sato, E., Fukumori, Y., et al. (2013). Anti-CCR4 mAb selectively depletes effector-type FoxP3+CD4+ regulatory T cells, evoking antitumor immune responses in humans. Proc. Natl. Acad. Sci. USA *110*, 17945–17950.

Takahashi, K., and Yamanaka, S. (2006). Induction of pluripotent stem cells from mouse embryonic and adult fibroblast cultures by defined factors. Cell *126*, 663–676.

Takahashi, K., Tanabe, K., Ohnuki, M., Narita, M., Ichisaka, T., Tomoda, K., and Yamanaka, S. (2007). Induction of pluripotent stem cells from adult human fibroblasts by defined factors. Cell *131*, 861–872.

Taner, T., Hackstein, H., Wang, Z., Morelli, A.E., and Thomson, A.W. (2005). Rapamycin-treated, alloantigen-pulsed host dendritic cells induce Ag-specific T cell regulation and prolong graft survival. Am. J. Transplant. *5*, 228–236.

Thomson, A.W., Turnquist, H.R., Zahorchak, A.F., and Raimondi, G. (2009). Tolerogenic dendritic cell-regulatory T-cell interaction and the promotion of transplant tolerance. Transplantation *87*, S86–S90.

Tiao, M.M., Lu, L., Tao, R., Wang, L., Fung, J.J., and Qian, S. (2005). Prolongation of cardiac allograft survival by systemic administration of immature recipient dendritic cells deficient in NF-kappaB activity. Ann. Surg. *241*, 497–505.

Turnquist, H.R., Raimondi, G., Zahorchak, A.F., Fischer, R.T., Wang, Z., and Thomson, A.W. (2007). Rapamycin-conditioned dendritic cells are poor stimulators of allogeneic CD4+ T cells, but enrich for antigen-specific Foxp3+ T regulatory cells and promote organ transplant tolerance. J. Immunol. *178*, 7018–7031.

Wan, Y.Y., and Flavell, R.A. (2007). 'Yin-Yang' functions of transforming growth factor-beta and T regulatory cells in immune regulation. Immunol. Rev. *220*, 199–213.

Wing, K., Onishi, Y., Prieto-Martin, P., Yamaguchi, T., Miyara, M., Fehervari, Z., Nomura, T., and Sakaguchi, S. (2008). CTLA-4 control over Foxp3+ regulatory T cell function. Science *322*, 271–275.

Wood, K.J., Bushell, A., and Hester, J. (2012). Regulatory immune cells in transplantation. Nat. Rev. Immunol. *12*, 417–430.

Zhang, X., Li, M., Lian, D., Zheng, X., Zhang, Z.X., Ichim, T.E., Xia, X., Huang, X., Vladau, C., Suzuki, M., et al. (2008). Generation of therapeutic dendritic cells and regulatory T cells for preventing allogeneic cardiac graft rejection. Clin. Immunol. *127*, 313–321.

Zhang, N., Schroppel, B., Lal, G., Jakubzick, C., Mao, X., Chen, D., Yin, N., Jessberger, R., Ochando, J.C., Ding, Y., et al. (2009a). Regulatory T cells sequentially migrate from inflamed tissues to draining lymph nodes to suppress the alloimmune response. Immunity *30*, 458–469.

Zhang, Q., Iwami, D., Aramaki, O., Yakubo, S., Nishimura, K., Ishige, A., Watanabe, K., Matsuno, K., Shirasugi, N., and Niimi, M. (2009b). Prolonged survival of fully mismatched cardiac allografts and generation of regulatory cells by Sairei-to, a Japanese herbal medicine. Transplantation *87*, 1787–1791.

Zhang, Q., Fujino, M., Iwasaki, S., Hirano, H., Cai, S., Kitajima, Y., Xu, J., and Li, X.K. (2014). Generation and characterization of regulatory dendritic cells derived from murine induced pluripotent stem cells. Sci. Rep. *4*, 3979.

Stem Cell Reports, Volume *8*

Supplemental Information

iPSC-Derived Regulatory Dendritic Cells Inhibit Allograft Rejection by Generating Alloantigen-Specific Regulatory T Cells

Songjie Cai, Jiangang Hou, Masayuki Fujino, Qi Zhang, Naotsugu Ichimaru, Shiro Takahara, Ryoko Araki, Lina Lu, Ji-Mei Chen, Jian Zhuang, Ping Zhu, and Xiao-Kang Li

Supplementary Information

Supplemental Experimental Procedures:

DC culture, related to main-text Result 1

We induced the differentiation of iPS cells and the B6 embryonic fibroblast-derived iPS cell line (2A-4F-100, H-2^b)(Araki et al., 2013) in DCregs mainly according to the protocol based on the method by Senju et al. (Senju et al., 2009). Steps 1 and 2 were described previously (Zhang et al., 2014), while step 3 was modified in this study as follows (**fig. S1, A**). For step 3, the cells at the end of step 2 were cultured in a 24-well hydrocells plate (CellSeed Inc., Tokyo, Japan) as $5x10^5$ per well in 1ml RPMI-1640 medium (Life Technologies, Gaithersburg, MD) supplemented with antibiotics, 10% (vol./vol.) fetal calf serum (Gibco, Carlsbad, CA) and 50mper well in 1ml RPMI-1640 medium (Life Tec the complete medium). iPS-DCcons were in the presence of GM-CSF (20ng/ml), IL-4 (10ng/ml). iPS-DCregs were in the presence of GM-CSF (20ng/ml), TGF-β1 (20ng/ml), IL-10 (20ng/ml) (PeproTech, London, UK). On day 5 of step 3, the cells were stimulated with IFN-γ (10ng/ml) for 48 hours and harvested on day 7. The iPS-DCcons used in this

study contained >87.4% $CD11b^+CD11c^+$ cells, while the iPS-DCregs used in this study contained >81.4% $CD11b^+CD11c^+$ cells.

Bone marrow cells (BMCs) were collected from the femoral and tibial of B6 or BALB/c. For the generation of BM-DCcons, BMCs were cultured in a 24-well TC plate (Greiner bio-one, Tokyo, Japan) as 1×10^6 per well with the complete medium in the presence of GM-CSF (10ng/ml), IL-4 (10ng/ml). IFN-γ (10ng/ml) was added at day 5 with two additional days for maturation. For the generation of BM-DCregs, BMCs were cultured in a 24-well TC plate as 1×10^6 per well with the complete medium in the presence of GM-CSF (20ng/ml), TGF-β1 (20ng/ml) and IL-10 (10ng/ml). IFN-γ (10n g/ml) was added on day 5 with two additional days for maturation.

Heterotopic cardiac transplantation, related to figure 3, 5, 6

All transplant procedures were performed under anesthesia with isoflurane. Fully vascularized heterotopic hearts from B6 or BALB/c were transplanted into CBA recipients using a microsurgical technique (Cai et al., 2016). The beats of grafts were manipulated

daily for the first two weeks and twice weekly for three months after transplantation. Graft survival was considered complete at the time of cessation of a palpable heart beating, and confirmed visually by laparotomy.

Isolation of lymphocytes from grafts, related to figure 4

Grafts were collected and cut into 1-2mm pieces on ice. Then the tissue was disrupted mechanically in 10ml digestion solution, which include 0.5mg/ml collagenase IV (Sigma), 50U/ml DNaseI (Invitrogen) in Phosphate buffered saline (PBS) (Gibco), and incubated at 37 °C for 20 minutes. After that, 10ml iced RPMI 1640 with 5% fetal calf serum was added. The suspension was filtered through a nylon mesh (100 μm) to remove aggregates. The resulted cell suspension was centrifuged at 200 × g for 10 minutes to pellet the cells. The pellet was suspended in 5ml PBS, loaded onto 5ml Lympholyte-M (Cedarlane, Canada) and centrifuged at 1500 × g for 25 minutes at room temperature. Cells were isolated from the Lympholyte-M interface and washed twice in PBS at 300× g for 5 minutes and prepared for FCM assay.

Mixed leukocyte reactions (MLR), related to figure 1, 2

One-way MLR culture was performed in duplicate in 96-well, round-bottom plates (Greiner bio-one). Nylon wool-eluted spleen T cells (2×10^5 /well) were labeled with CellTrace Violet (Life Technologies) and used as responders. Cultures were maintained in the complete medium for 3-5 days in 5% CO_2 in air. The reaction system and other details are shown in the associated figure legends.

Popliteal lymph node (PLN) assay, related to figure 1

Splenic T cells were isolated from OT-I and OT-II mice using a nylon-wool column and labeled with Violet (OT-I) or CFSE (Life Technologies) (OT-II). Prepared OT-I or OT-II T cells were adoptively transferred to naïve B6 mice at 3×10^6 /500ml/mice at day -1. DCs were pulsed with OVA as 400 mg/ml for 24 hours and then were injected subcutaneously into the recipients' footpads at day 0. The negative control mice were

treated with PBS. The PLN were harvested at day 3. The reaction system and other details are shown in the associated figure legends.

Flow cytometry, related to all figures,

Expression of DCs surface antigens was analyzed by Attune acoustic focusing cytometer (Applied Biosystems, Carlsbad, CA). Cells were stained with CD11b-PE/Cy7 (Cat. 101216; negative control: PE/Cy7 Rat IgG2b, κ Isotype Ctrl, Cat. 400618; Biolegend, San Diego, CA), CD11c-APC (Cat. 117310; negative control: APC Armenian Hamster IgG Isotype Ctrl, Cat. 400912; Biolegend), and FITC-conjugated CD40 (Cat. 124608; negative control: FITC Rat IgG2a, κ Isotype Ctrl, Cat. 400506; Biolegend), CD80 (Cat. 104706; negative control: FITC Armenian Hamster IgG Isotype Ctrl, Cat. 400906; Biolegend), CD86 (Cat. 105110; negative control: FITC Rat IgG2b, κ Isotype Ctrl, Cat. 400606; Biolegend), IA-IE (Cat. 107606; negative control: FITC Rat IgG2b, κ Isotype Ctrl, Cat. 400605; Biolegend). Antigen uptake test of DCs was analyzed by Attune (Applied Biosystems). Cells were stained with CD11b-PE/Cy7, CD11c-APC, and FITC-conjugated

OVA (Cat. O23020, Lifetechnologies, Carlsbad, CA) or Dextran (Cat. D1844, Lifetechnologies). SPCs isolated from recipients after heart transplantation were stained with CD4-Pacific orange (Cat. MCD0430; negative control: Pacific orange Rat IgG2a Isotype Control, Cat. R2a30; Lifetechnologies), CD25-APC (Cat. 102012; negative control: APC Rat IgG1, λ Isotype Ctrl, Cat. 401904; Biolegend), CTLA4-PE (Cat. 106306; negative control: PE Armenian Hamster IgG Isotype Ctrl, Cat. 400908; Biolegend), GITR-PE (Cat. 126310; negative control: PE Rat IgG2b, κ Isotype Ctrl, Cat. 400608; Biolegend), TGF-β1-PE (Cat. 141404; negative control: PE Mouse IgG1, κ Isotype Ctrl, Cat. 400112; Biolegend), CD8α-PE/Cy7 (Cat. 100722; negative control: PE/Cy7 Rat IgG2a, κ Isotype Ctrl, Cat. 400522; Biolegend) and FOXP3-PE (Cat. 12-5773-82; negative control: PE Rat IgG2a K Isotype Ctrl, Cat. 12-4321-42; eBioscience, Santa Clara, CA). GILs were stained with CD4-FITC (Cat. 100510; negative control: FITC Rat IgG2a, κ Isotype Ctrl; Biolegend) or CD4-APC (Cat. 100516; negative control: APC Rat IgG2a, κ Isotype Ctrl, Cat. 400512, Biolegend), CD25-APC/Cy7 (Cat. 102026, Biolegend; negative control: APC/Cy7 Rat IgG1, λ Isotype Ctrl, Cat. 557663; BDBiosciences), CTLA4-APC (Cat.

106310; negative control: APC Armenian Hamster IgG Isotype Ctrl; Biolegend), GITR-PE, CD8α-PE/Cy7 and FOXP3-PE. For FOXP3 staining, Transcription Factor Fixation/Permeabilization Concentrate and Diluent (eBioscience) was used. SPCs were analyzed by Gallios flow cytometer (Beckman Coulter, Brea, CA) and GILs were analyzed by Attune. Cells from MLR were stained with CD4-APC and CD8α-PE/Cy7; and were detected by Gallios. SPCs isolated from CBA, which only accepted iPS-DCregs and anti-TGFβ1 mAb but without heart transplantation, were stained with: 1) TGF-β1-PE; 2) CD4-FITC, CD25-APC/Cy7, Ki67-PE/Cy7 (Cat. 652426; negative control: PE/Cy7 Rat IgG2a, κ Isotype Ctrl; Biolegend), CCR4-APC (Cat. 131212; negative control: APC Armenian Hamster IgG Isotype Ctrl; Biolegend) and FOXP3-PE; and 3) CD4-APC, CD25-APC/Cy7, Ki67-FITC (Cat. 652410; negative control: FITC Rat IgG2a, κ Isotype Ctrl; Biolegend), CCR7-PE/Cy7 (Cat. 120124; negative control: PE/Cy7 Rat IgG2a, κ Isotype Ctrl; Biolegend) and FOXP3-PE. This test was detected by Attune. All flow cytometric data were analyzed with FLOWJO V.10.0.8 (FLOWJO LLC, Ashland, OR).

Histopathology, related to figure 3

Grafts were harvested on POD7 and POD14 were fixed in 10% formalin solution (WAKO, Osaka, Japan) and then embedded in paraffin. Sections of 4μm were made for hematoxylin and eosin (HE) staining.

Immunohistochemistry, related to figure 3, 4

Grafts and spleens were harvested on POD7 and POD14. 5-bromo-2-deoxyuridine (BrdU) was injected to the recipients 1 hour before sampling. Cryo-blocks were immediately frozen through Hexane (WAKO) in -80 °C. Sections of 4 μm were cut for immunohistochemistry staining. Triple immunostaining was performed. The rat anti-mouse CD4 mAb (Cat. 100506, Biolegend), rat anti-mouse CD8α mAb (Cat. 100802, Biolegend) and anti-mouse FOXP3 biotin mAb (Cat. 13-5773-82, eBioscience) were used as the primary antibodies. After being rehydrated, sections were blocked with 4% block ace (Dainippon Sumitomo, Osaka, Japan) for 10 minutes. Primary antibodies were used as a 1:100 dilution, and sections were incubated in primary antibody working solution at room

temperature for 1 hour and then washed by PBS. Secondary antibody: ALP-conjugated donkey anti-rat IgG (Cat. 712-005-150 , Jackson Immuno Research, West Grove, PA) for rat anti-mouse CD4 mAb, rat anti-mouse CD8α mAb incubated samples and ALP-conjugated anti-biotin antibody (Cat. A6561, Sigma-Aldrich, St. Louis, MO) for anti-mouse FOXP3 biotin mAb incubated samples. Secondary antibodies were used as 1:100 dilution and heat-inactivated normal mouse serum were added to working solution as 1% final concentration. Sections were incubated in secondary antibody working solution at room temperature for 1 hour. Labeled proteins were visualized with Vector-Blue kit (VECTOR Laboratories, Burlingame, UK) according to the manufacturer's instructions. Thereafter, cryo-sections were incubated with rabbit-anti-mouse type IV collagen polyclonal Ab (Cat. LB1403, Cosmo Bio, Tokyo, Japan) diluted as 1:3000 for 1 hour, and then incubated with HRP-conjugated goat anti-rabbit Ig (Cat. 08674191, MP Biomedicals, Santa Ana, CA) as 1:100 and developed with DAB Peroxidase Substrate Kit (Vector Laboratories). Subsequently, the samples were incubated at 37 °C with pepsin-HCl solution for 12 minutes then treated with 4N.HCl for 30 minutes at room temperate and neutralized

with borax buffer (0.1 M; pH 8.5) for 4 minutes. BrdU was then detected with the rat anti-BrdU mAb (Cat. OBT0030CX, AbD Serotec, Raleigh, NC) followed by the ALP-conjugated donkey anti-rat Ig (Jackson Immuno Research). Sections were visualized with New Fuchsin Substrate System (Dako, Carpinteria, CA). Finally, sections were fixed in FCa solution (4% Paraformaldehyde /PBS +1%$CaCl_2$) for 10 minutes and then mounted in Aquatex (Merck, Whitehouse Station, NJ).

RNA Preparation and Quantitative Reverse Transcriptase-polymerase Chain Reaction (qRT-PCR), related to figure 3, 4, 6

Cardiac grafts were harvested on POD7 and POD14 and submerged in RNAlater® stabilization solution (Life Technologies, Carlsbad, CA) for freezing. Total RNA was extracted from frozen tissue samples using ISOGEN (NipponGene, Tokyo, Japan). DCs were directly frozen at -80 °C and total RNA was extracted by RNeasy Mini Kit (QIAGEN, Limburg, Nederland). Then, RNA was reverse transcribed to cDNA using PrimeScript® RT reagent Kit (Takara Bio, Shiga, Japan). Quantitative RT-PCR was performed using a

TaqMan system on the Applied Biosystems PRISM7700 instrument (Applied Biosystems). The normalized threshold cycle (Ct) value of each gene was obtained by subtracting the Ct value of 18S rRNA. The sequences used in our study are shown in **Table S1**.

Adoptive transfer study, related to figure 5

Splenocytes (5×10^7) isolated from the primary recipients bearing accepted B6 cardiac allografts after iPS-DCregs therapy on POD100 were adoptively transferred into naïve CBA mice (secondary recipients) on day -1. Then, the secondary recipients underwent transplantation of B6 or BALB/c (third party) cardiac grafts on day 0. The schematics of the protocol are shown in the associated figures.

Supplementary Figure:

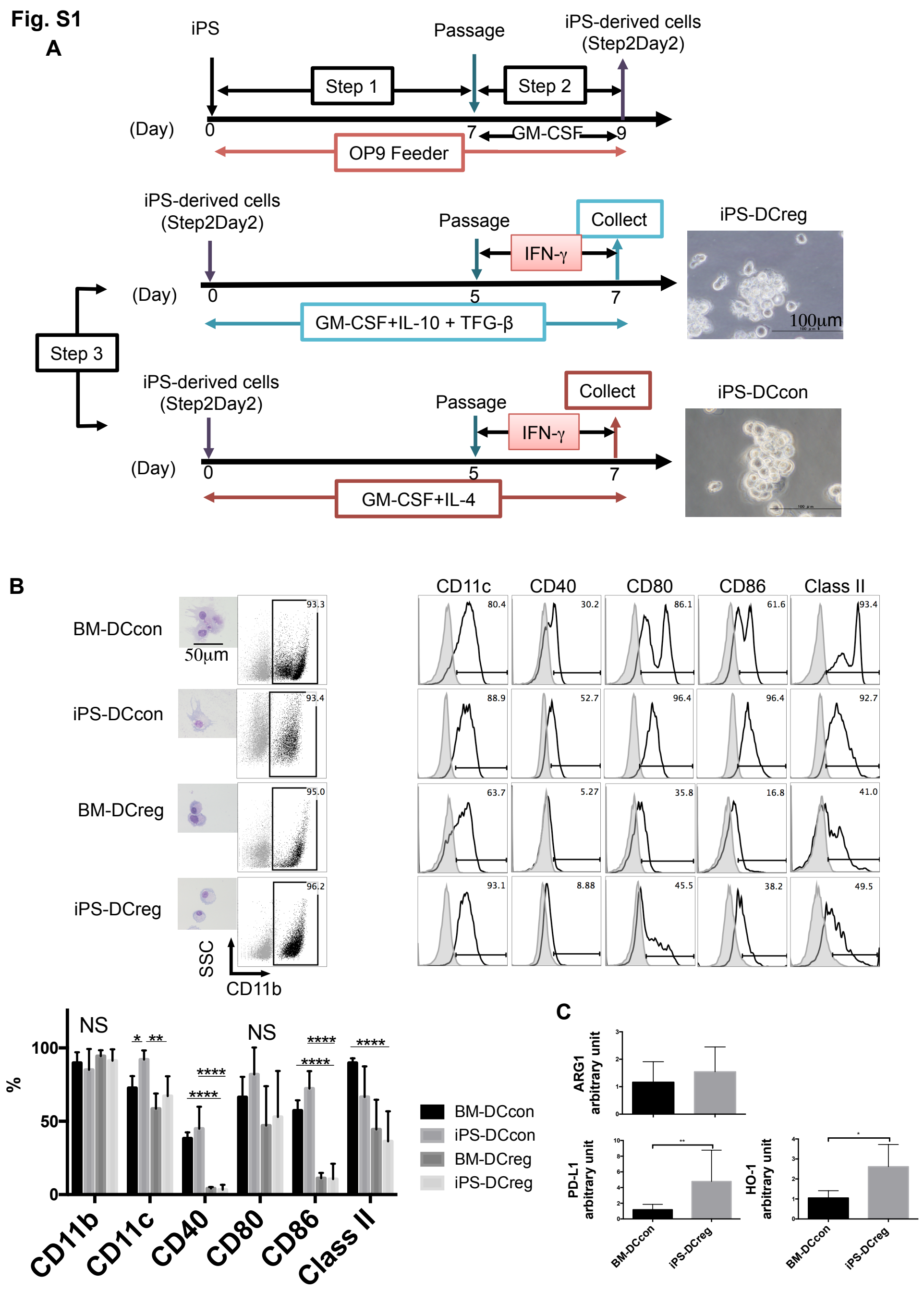

Fig. S1. *Generation protocol and phenotype of iPS-DCregs, related to main-text Experimental Procedures and Result 1.* (**A**) Generation protocol of iPS-DCs is shown in schema. (**B**) The morphology of DCs is shown as May-Grunwald and Giemsa staining (original magnification×400). The percentage of $CD11b^+$ is shown as dot plot. The expression of CD11c, co-stimulatory molecules and MHC-II antigen is displayed as histograms gated on the $CD11b^+$ cell population. Grey lines show isotype control. (n=9 in BM-DCcon, n=5 in iPS-DCcon, n=6 in BM-DCreg, n=8 in iPS-DCreg, mean ± SD). Statistical analysis was determined by one-way ANOVA and Tukey's test. *$p<0.05$, **$p<0.01$, ****$p<0.0001$. (**C**) The mRNA expression of ARG1, PD-L1, HO-1 was detected by qRT-PCR (n=5 in each group, mean ± SD). Statistical analysis was determined by Student's t test. *$p<0.05$, **$p<0.01$.

Fig. S2

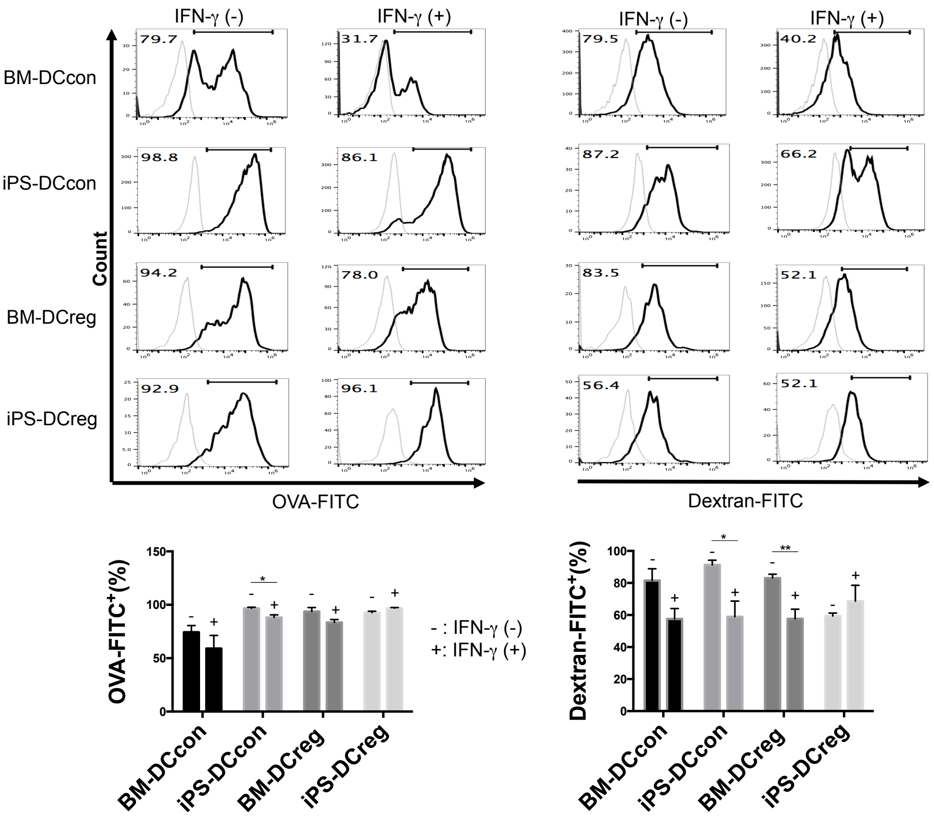

Fig. S2. *Antigen uptake ability test of iPS-DCregs before and after IFN-γ stimulation, related to main-text Result 1.* DCs (**fig. S1, A**) were harvested on day 5 (without IFN-γ stimulation) and day 7 (48-hr IFN-γ stimulation) for protein antigen (OVA-FITC) and carbohydrate antigen (Dextran-FITC) uptake analysis (n=4 in each group, mean ± SD).

Statistical analysis was determined by one-way ANOVA and Tukey's test. $^{*}p<0.05$, $^{**}p<0.01$.

Fig. S3

A

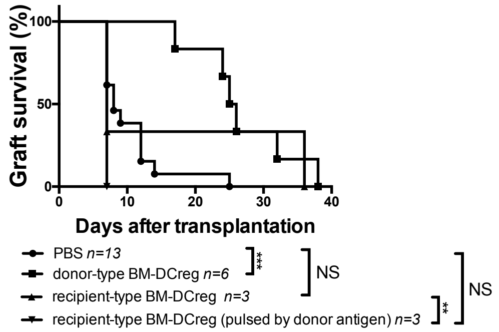

Group	*n*	Graft survival days	MST
PBS	13	7×5, 8×2, 9×1, 12×3, 14, 25	8
donor-type BM-DCreg	6	17, 24, 25, 26, 32, 38	25.5
recipient-type BM-DCreg	3	7×2, 36	7
recipient-type BM-DCreg (pulsed by donor antigen)	3	7×3	7

B

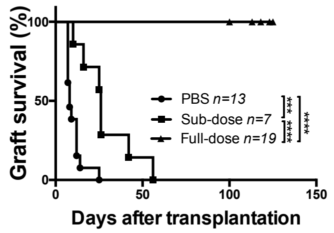

Group	*n*	Graft survival days	MST
PBS	13	7×5, 8×2, 9×1, 12×3, 14, 25	8
iPS-DCreg Sub-dose	7	7 10, 16, 25, 26×2, 42, 56	26
iPS-DCreg Full-dose	19	>100×19	>100

Fig. S3. *Different origins and different doses of DCregs lead to different allografts protection effect, related to main-text Result 4.* (**A**) 1×10^6 CBA (recipient-type) derived

BM-DCregs (with or without donor-antigen pulsed) or B6 (donor-type) derived BM-DCregs were i.v. into CBA (recipient) mice 7 days before heterotopic cardiac transplantation. A statistical evaluation of the graft survival was performed using the Kaplan-Meier curves and compared using the Log-Rank tests. ****$p<0.001$*. (**B**) 1×10^6 (full-dose) or 5×10^5 (sub-dose) B6 (donor-type) derived iPS-DCregs were i.v. into CBA (recipient) mice 7 days before heterotopic cardiac transplantation. A statistical evaluation of the graft survival was performed using the Kaplan-Meier curves and compared using the Log-Rank tests. ****$p<0.001$*, *****$p<0.0001$*.

Fig. S4

A

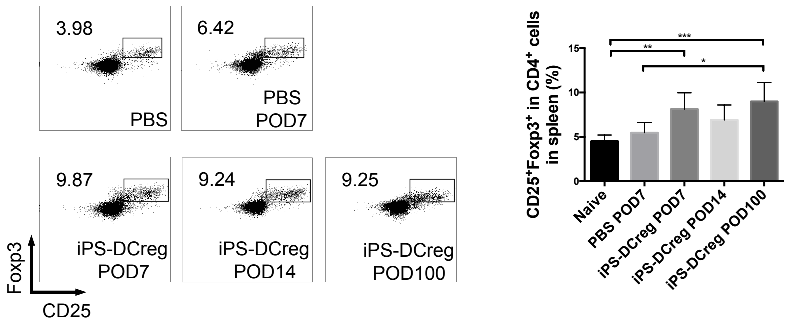

3.98
PBS
6.42
PBS
POD7
9.87
iPS-DCreg
POD7
9.24
iPS-DCreg
POD14
9.25
iPS-DCreg
POD100
Foxp3
CD25
CD25+Foxp3+ in CD4+ cells in spleen (%)
Naive
PBS POD7
iPS-DCreg POD7
iPS-DCreg POD14
iPS-DCreg POD100

B

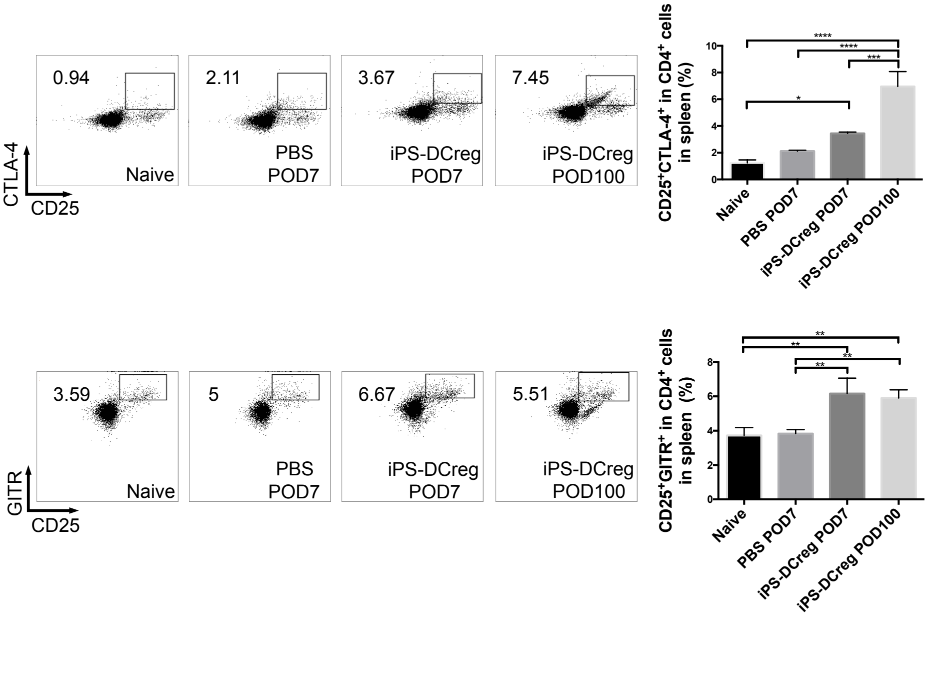

0.94
Naive
2.11
PBS
POD7
3.67
iPS-DCreg
POD7
7.45
iPS-DCreg
POD100
CTLA-4
CD25
CD25+CTLA-4+ in CD4+ cells in spleen (%)
Naive
PBS POD7
iPS-DCreg POD7
iPS-DCreg POD100
3.59
Naive
5
PBS
POD7
6.67
iPS-DCreg
POD7
5.51
iPS-DCreg
POD100
GITR
CD25
CD25+GITR+ in CD4+ cells in spleen (%)
Naive
PBS POD7
iPS-DCreg POD7
iPS-DCreg POD100

Fig. S4. *Activated Tregs expanded in spleen from iPS-DCregs immunized recipients after allo-transplantation, related to main-text Result 6.* (**A**) Spleens were harvested on POD7, 14 and 100, and were triple stained for CD4/CD25/FOXP3 ($n=7$ in naïve control, $n=5$ in PBS-POD7, $n=6$ in iPS-DCregs-POD7, $n=4$ in iPS-DCregs-POD14 and POD100, mean ± SD). Statistical analysis was determined by one-way ANOVA and Tukey's test. *$p<0.05$, **$p<0.01$, ***$p<0.001$. (**B**) Spleens were harvested on POD7 and 100, and were triple stained for CD4/CD25/CTLA-4 or CD4/CD25/GITR ($n=3$ in naïve control, PBS-POD7 and iPS-DCregs-POD7, $n=4$ in iPS-DCregs-POD100, mean ± SD). Statistical analysis was determined by one-way ANOVA and Tukey's test. *$p<0.05$, **$p<0.01$, ***$p<0.001$, ***$p<0.0001$.

Fig. S5

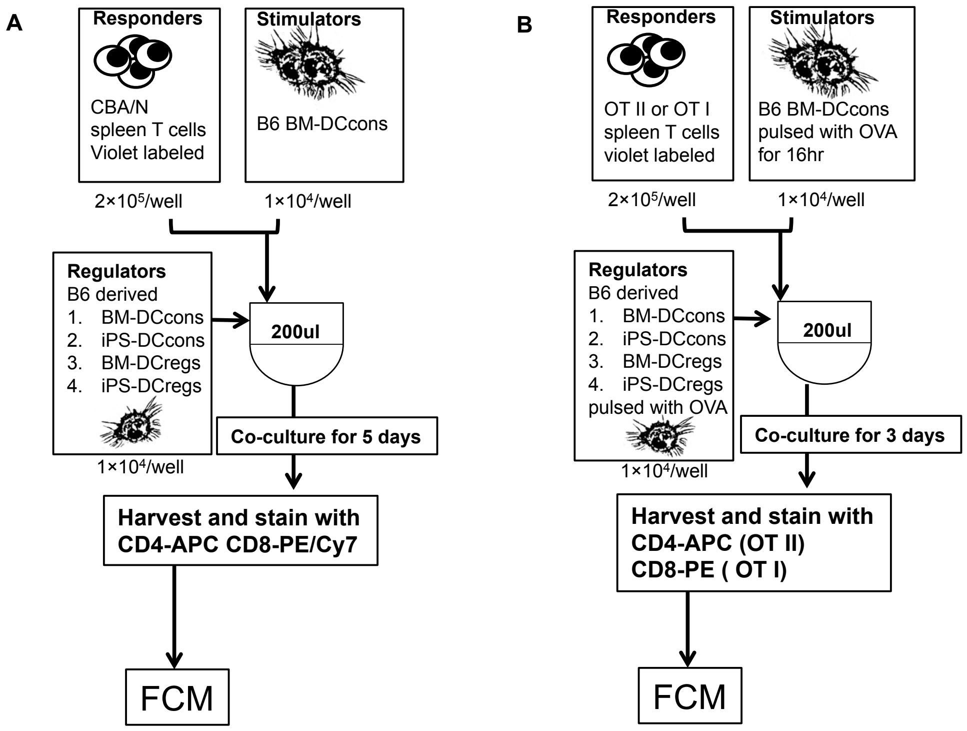

Fig. S5. *MLR reactive system used in this study, related to main-text figure 1, 2*. (**A**) Violet-labeled CBA T cells (responder) were cultured with B6 BM-DCcons (stimulator) at a ratio of 20:1 for 5 days. Four types of DCs were added at the beginning into the culture as a regulator (regulator: stimulator 1:1). Proliferation of T cells was determined by Violet dilution gated on $CD4^+$ and $CD8^+$ population. (**B**) Violet-labeled OT-II or OT-I T cells (responder) were cultured with OVA-pulsed B6 BM-DCcons (stimulator) at a ratio of 20:1

for 3 days. Four types of DCs were added into the culture as regulators (regulator: stimulator 1:1). Proliferation of T cells was determined by Violet dilution gated on $CD4^+$ (OT-II) or $CD8^+$ (OT-I) populations.

Table S1. Primer Sets Used in This Study, Related to Figure 3, 4, 6

Genes	Forward Primer	Reverse Primer	Probe
**IL-6**	5' CTgCAAgTgCATCATCgTTgT 3'	5' TgTCTATACCACTTCACAAgTCggA 3'	5' CAgAATTgCCATTgCACAACTCTTTTCTCA 3
**IL-1β**	5' TgAAAgACggCACACCCA 3'	5' gACAAACCgCTTTTCCATCTTC 3'	5' CAgCTggAgAgTgTggATCCCAAACA 3'
**Granzyme B**	5' AgAgTggggCTTgACTTCATgT 3'	5' TCCTgCTACTgCTgACCTTgTCT 3'	5' CCCgATgATCTCCCCTgCCTTTgT 3'
**Perforin**	5' AAgTCAAggTggAgTggAggTTT 3'	5' gCTgAgAAgACCTATCAggACCA 3'	5' CCAggCgAAAACTgTACATgCgACAC 3'
**HO-1**	5' CAgggTgACAgAAgAggCTAAgAC 3'	5' TTgTgTTCCTCTgTCAgCATCAC 3'	5' TCCTgCTCAACATTgAgCTgTTTgAggA 3'
**iNOS**	5' CAgTggAgAgATTTTgCATgACA 3'	5' CCCCAAgCAAgACTTggACTT 3'	5' CCACAAggCCACATCggATTTCACTT 3'
**TNF-α**	5' TgTCTACTgAACTTCggggTgAT 3'	5' AACTgATgAgAgggAggCCAT 3'	5' TCCCCAAAgggATgAgAAgTTCCCAA 3'
**Arg-1**	5' TgCTgCAgggCCTTTCTC 3'	5' gAgCTCCAAgCCAAAgTCCTT 3'	5' CCTCCTCgAggCTgTCCTTTTgAgAAAg 3'
**TGF-β1**	5' AggTCACCCgCgTgCTAA 3'	5' gCTTCCCgAATgTCTgACgTA 3'	5' TggACCgCAACAACgCCATCTATg 3'

**IFN-γ**	5' CAAggCgAAAAAggATgCA 3'	5' CggATgAgCTCATTgAATgCT 3'	5' TgCCAAgTTTgAggTCAACAACCCACA 3'
**FOXP3**	5' gCCCACCAgTACAgCTggA 3'	5' CAAgTCTCgTCTgAAggCAgAgT 3'	5' CCACTCCAgCTCCCggCAACTTC 3'
**PD-L1**	5' AgCCAgggCAAAACCACA 3'	5' AgCACCCAgTgAgTCCTgTTCT 3'	5' TgATCATCCCAgAACTgCCTgCAACA 3'
**18S**	5' ATgAgTCCACTTTAAATCCTTTAACgA 3'	5' CTTTAATATACgCTATTggAgCTggAA 3'	5' ATCCATTggAgggCAAgTCTggTgC 3'

Supplementary Reference

Araki, R., Uda, M., Hoki, Y., Sunayama, M., Nakamura, M., Ando, S., Sugiura, M., Ideno, H., Shimada, A., Nifuji, A., *et al.* (2013). Negligible immunogenicity of terminally differentiated cells derived from induced pluripotent or embryonic stem cells. Nature *494*, 100-104.

Cai, S., Ichimaru, N., Zhao, M., Fujino, M., Ito, H., Ota, U., Nakajima, M., Tanaka, T., Nonomura, N., Li, X.K., *et al.* (2016). Prolonged Mouse Cardiac Graft Cold Storage via Attenuating Ischemia-Reperfusion Injury Using a New Antioxidant-Based Preservation Solution. Transplantation *100*, 1032-1040.

Senju, S., Haruta, M., Matsunaga, Y., Fukushima, S., Ikeda, T., Takahashi, K., Okita, K., Yamanaka, S., and Nishimura, Y. (2009). Characterization of dendritic cells and macrophages generated by directed differentiation from mouse induced pluripotent stem cells. Stem cells *27*, 1021-1031.

Zhang, Q., Fujino, M., Iwasaki, S., Hirano, H., Cai, S., Kitajima, Y., Xu, J., and Li, X.K. (2014). Generation and characterization of regulatory dendritic cells derived from murine

induced pluripotent stem cells. Scientific reports *4*, 3979.
